# Role of insulinemic and inflammatory dietary patterns on gut microbial composition and circulating biomarkers of metabolic health among older American men

**DOI:** 10.1080/19490976.2025.2497400

**Published:** 2025-04-28

**Authors:** Sushma Nepal, Ni Shi, Rebecca Hoyd, Daniel J. Spakowicz, Eric Orwoll, James M. Shikany, Nicola Napoli, Fred K. Tabung

**Affiliations:** a Interdisciplinary Ph.D. Program in Nutrition, The Ohio State University, Columbus, OH, USA; b Comprehensive Cancer Center, The Ohio State University, Columbus, OH, USA; c Division of Medical Oncology, Department of Internal Medicine, The Ohio State University Wexner Medical Center, Columbus, OH, USA; d Department of Medicine, Oregon Health & Sciences University, Portland, OR, USA; e Division of General Internal Medicine and Population Science, Heersink School of Medicine, University of Alabama at Birmingham, Birmingham, AL, USA; f Unit of Endocrinology and Diabetes, Department of Medicine, Campus Bio-Medico University of Rome, Rome, Italy; g Division of Bone and Mineral Diseases, Washington University, St Louis, MO, USA; h Division of Epidemiology, College of Public Health, The Ohio State University, Columbus, OH, USA

**Keywords:** Dietary patterns, gut microbiome, biomarkers of chronic diseases

## Abstract

Chronic low-grade inflammation and hyperinsulinemia are linked with metabolic dysfunction and dysbiosis. This study investigated the role of dietary inflammatory and insulinemic potential on gut microbiome and circulating health biomarkers in older men. Data from the Osteoporotic Fractures in Men (MrOS) study were analyzed. Reversed Empirical Dietary Inflammatory Pattern (rEDIP), Empirical Dietary Index for Hyperinsulinemia (rEDIH), and Healthy Eating Index (HEI)-2020 scores were computed from food frequency questionnaire data. Stool samples were profiled using 16S rRNA sequencing. Elastic net regression identified diet-associated microbial profiles and multivariable-adjusted linear regression assessed diet-biomarker associations. Higher rEDIP, rEDIH, and HEI-2020 scores were positively associated with gut microbiota alpha diversity. Specific genera, including *Intestinibacter and Lachnospira*, associated positively, while *Dielma*, *Peptococcus*, *Feacalitalea*, and *Negativibaccilus* associated inversely with healthier dietary patterns. When evaluating changes in dietary patterns between baseline and visit 4 ( ~ 14 years), these genera tended to define rEDIP, rEDIH more than HEI-2020. In addition, higher dietary quality was linked to better biomarker profiles, including lower creatinine, sodium, triglycerides, and insulin resistance. Beneficial effects of higher dietary quality on health may be mediated by the ability of diet to regulate gut microbial composition and metabolic biomarker profiles.

## Introduction

Chronic low-grade inflammation is a hallmark of many metabolic disorders,^1^ significantly contributing to insulin resistance and other metabolic disruptions.^2,3^ This inflammatory state can further perpetuate itself, creating a vicious cycle of inflammation and metabolic dysfunction.^2^ Both inflammation and hyperinsulinemia profoundly affect the gut microbiome, influencing its composition and function.^4,5^ The imbalance in gut microbiota, known as dysbiosis, can further reinforce the cycle of inflammation, hyperinsulinemia, and metabolic dysfunction, contributing to the progression of chronic diseases.^2,6^

Diet plays a critical role in modulating inflammation, hyperinsulinemia, and gut microbiota composition.^7–9^ Although multiple studies have explored the relationships between diet and these variables, few have examined the inflammatory and insulinemic dietary patterns in relation to the gut microbial composition.^10^ The Empirical Dietary Inflammatory Pattern (EDIP)^11^ and Empirical Dietary Index for Hyperinsulinemia (EDIH)^12^ are metabolically-derived dietary patterns that have shown significant associations with multiple chronic metabolic diseases, including several cancers,^7,13,14^ diabetes,^8^ obesity,^15^ cardiovascular diseases,^16^ and related biomarkers.^17^ These dietary patterns were developed based on biomarkers of chronic metabolic diseases and assess the quality of the overall diet based on its ability to regulate chronic inflammation or insulin hypersecretion, respectively. In this study, we also utilized the Healthy Eating Index 2020 (HEI-2020), a dietary pattern score developed to assess overall dietary quality according to the Dietary Guidelines for Americans (DGA).^18^

Our objectives in the current study were to identify the gut microbial profiles of EDIP and EDIH and compare with overall dietary quality, measured via HEI-2020. In addition, we examined associations of the dietary pattern indices with circulating biomarkers of renal function, insulin response, and lipid metabolism.

## Methods

### Study population

**Figure 1.**
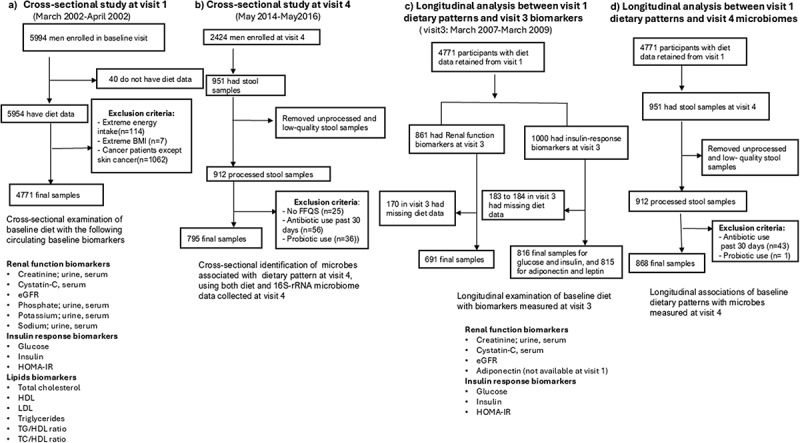
Combined flow chart of study design and participant selection: MrOS study cohort.

The Osteoporotic Fractures in Men (MrOS) study is a large prospective cohort study involving 5,994 community-dwelling older men aged ≥65 years, recruited from six clinical sites across the U.S., between 2000 and 2002 (https://mrosonline.ucsf.edu). The study’s design and recruitment methods have been previously described.^19,20^ Institutional review boards at all participating sites approved the study, and all participants provided written informed consent. Out of the 5,994 men at MrOS baseline, 40 were excluded due to missing dietary data, 114 due to too low/high energy intake levels (<600 or > 5000 kcal/day), 7 due to an extreme body mass index (BMI) (<15 and >50 kg/m^2^), and 1,062 men due to a history of cancer (excluding skin cancer), to constitute the final sample size for the biomarker analysis (Figure 1a).

For the gut microbiome component of the study, all surviving and active MrOS participants were invited to attend a follow-up visit (visit 4 at year 14 from baseline) conducted between May 2014 and May 2016. From this group, 1,329 men were invited to provide stool samples starting in 2015. Of those, 982 (73.9%) agreed to participate, and 951 stool samples were successfully collected. After removing unprocessed and low-quality samples, 912 were available for analysis. Low-quality samples were defined as those that failed the initial quality control check, which included inadequate DNA yield, sample degradation, or failure to meet collection, handling, or storage protocol standards. We further excluded 25 participants due to missing dietary data, along with 56 and 36 participants who reported recent antibiotic or probiotic use respectively, within 30 days prior to stool collection, resulting in a final sample size of 795 (Figure 1b). Additionally, the longitudinal analysis, which linked dietary data from visit 1 with biomarkers from visit 3 and microbiome data from visit 4, utilized different sample sets for each biomarker (Figure 1c). For the longitudinal microbiome analysis, a total of 868 samples were included (Figure 1d).

### Dietary assessment and computation of EDIH, EDIP, and HEI-2020 scores

The Block 98.2 brief food frequency questionnaire (FFQ)^21^ was used to assess the dietary behavior of MrOS participants. This questionnaire asked about the intake of 69 food items over the past year, along with 13 additional questions on food preparation and low-fat foods to refine nutrient calculations. Nutritional supplement use was also queried.^9^ It provided 9 frequency categories and 4 portion size options, with visual aids for standard portion sizes. A one-page supplement asked about the frequency and portion sizes of 9 common probiotic foods, such as yogurt and probiotic drinks. Nutrient composition was determined using the USDA Database for Standard References and the 1994–1996 Continuing Survey of Food Intakes by Individuals database.^9,22^ Dietary data were collected using these FFQs at visit 1 and visit 4.

Methods describing the derivation of each dietary score have been described in detail.^11,12^ These scores focus on overall dietary patterns rather than individual macronutrient and micronutrient intake. In this study, EDIP and EDIH scores were computed in a reversed manner (rEDIP and rEDIH), so that higher scores indicate a more anti-inflammatory or less hyperinsulinemic dietary pattern, aligning with the HEI-2020, where higher scores reflect higher dietary quality based on the DGA recommendations. The inversion allows for a more intuitive comparison with HEI scores, as all three indices now consistently associate higher scores with more favorable health outcomes.

### Microbiome data assessment

Stool sample collection methods were previously described.^23^ Participants collected samples at home using the OMNIgene GUT kit, which preserves microbial DNA. Samples were mailed to the MrOS Administrative Center for processing, where they were checked and stored at  − 80°C. A subset of 599 samples was sent to Baylor College of Medicine for gut microbiota profiling via 16S rRNA gene sequencing. 98.3% were collected within 30 days of visit 4. A second batch of 321 samples was sent for sequencing later. The remaining stool samples were excluded due to quality issues. Bacterial DNA was extracted and amplified targeting the 16S (v4) rDNA region, sequenced on the Illumina MiSeq platform, and processed using the bioBakery 16S workflow.

The Divisive Amplicon Denoising Algorithm 2 (DADA2) was used in R (v1.7.0) with default settings to correct amplicon sequence errors.^24^ DADA2 infers amplicon sequence variants (ASVs) directly, bypassing the need for operational taxonomic units (OTUs). Demultiplexed fastq files were filtered and trimmed to 240 bp (forward) and 200 bp (reverse) with maximum expected errors of 1 and 2.^25^ Error rates were estimated from at least 10^6^ reads, followed by dereplication, denoising, paired-read merging, and chimera removal. Taxonomy was assigned using the SILVA Database v128.^26^ The final dataset comprised 18,083,938 reads and identified 12,855 unique taxa.

### Functional analysis of predicted metagenomes

The Phylogenetic Investigation of Communities by Reconstruction of Unobserved States (PICRUSt2) pipeline (version 2.4.1)^27^ was employed to predict the functions of microbiota associated with rEDIH, rEDIP, and HEI-2020 scores. PICRUSt2 features a larger, updated database of gene families and reference genomes, supports any OTU-picking or denoising algorithm, and allows for phenotype predictions.

### Assessment of circulating biomarkers of chronic diseases

We obtained circulating biomarker data at baseline (visit 1) and visit 3 and assessed the relationship between the three dietary pattern indices and biomarkers of renal function, lipids, and insulin response (Figure 1a,c). For renal function biomarkers, fasting blood was collected. Cystatin C was determined nephelometrically in serum or plasma using Siemens ProSpec nephelometer. Serum creatinine, phosphate, sodium, total cholesterol, high-density lipoprotein cholesterol (HDL-C), low-density lipoprotein cholesterol (LDL-C), total cholesterol, and triglycerides were measured using a Roche COBAS Integra 800 automated analyzer (Roche Diagnostics Corps, Indianapolis, IN). Analyses of fasting glucose were performed enzymatically on a Hitachi 917 Autoanalyzer. Determination of insulin concentrations was performed by a 2-site immuno-enzymometric assay on a Tosoh 600 II auto-analyzer. Homeostatic Model Assessment of Insulin Resistance (HOMA-IR) was calculated from fasting insulin and fasting glucose data using the standard formula [HOMA-IR= insulin (µIU/ml) × glucose (mmol/L)/22.5].^28^

### Statistical analysis

**Figure 2.**
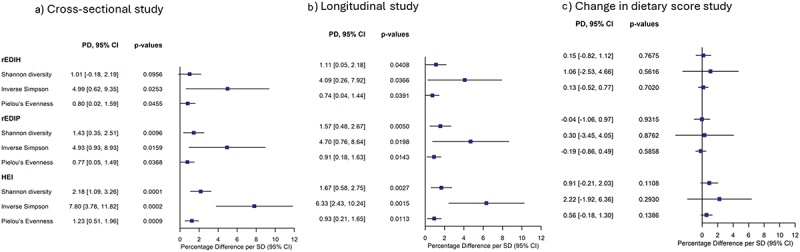
Cross-sectional, longitudinal, and change-in-dietary-score analyses of dietary patterns and gut microbiome alpha diversity measures. The absolute values, predicted min, max, for Shannon, inverse Simpson, and Pielou’s evenness in each study type are presented in supplementary table 2. PD, percentage difference; SD, standard deviation; CI, confidence interval.

Participants’ characteristics were summarized as frequencies (%) for categorical variables and means ± standard deviations (SD) for continuous variables across quintiles of the dietary indices (rEDIH, rEDIP, and HEI-2020) at visit 4 (microbiome analysis) and visit 1 (biomarker analysis). We designed three studies for the microbiome analysis – I) a cross-sectional study relating dietary data at visit 4 to microbiome data at visit 4 as the primary microbiome analysis, II) a longitudinal study relating diet at baseline to the gut microbiome at visit 4, and III) a change in dietary pattern study, where the change score was computed by subtracting visit 1 scores from visit 4 scores, such that higher change scores reflected healthier changes in the dietary patterns between baseline and visit 4. In all studies, the Shannon index, inverse Simpson index, and Pielou’s evenness index were calculated to determine the microbiome alpha diversity. We used multivariable linear regression to estimate the percentage change in alpha diversity for each 1-SD increment in the dietary indices, adjusting for the relevant covariates, in both analyses (Figure 2).

For the dietary pattern-related microbiome profile analysis, we aggregated the data at the genus level and performed a centered log-ratio transformation. To avoid issues with zero counts (since the log of zero is undefined), we added a pseudo counts of 1 before performing the transformation to genera present in more than 90% of participants. This ensured that all zero values are replaced by a minimal non-zero value. To identify microbiome profiles linked to each dietary pattern, we employed elastic-net regression. Elastic net regression is a regularized regression method that combines both Lasso and Ridge regression that is useful when dealing with highly correlated predictors. The dataset was randomly split, with 70% allocated for training and 30% for testing. Using ten-fold cross-validation in the training dataset to determine the optimal balance between model complexity and goodness of fit, and an alpha = 0.5, we regressed rEDIH, rEDIP, and HEI-2020 on the genera from the 16S rRNA data. The trained model was then applied to the testing set to calculate microbiome profile scores, which were derived as the weighted sum of selected genera, with weights determined by elastic-net regression coefficients. Lastly, we applied the tested model to the entire dataset to compute the final microbiome scores. We repeated this analysis for the cross-sectional study, longitudinal study, and the dietary score change study.

For the functional analyses, we used PICRUSt2 to predict functional pathways of the microbiome selected from the elastic net regression for each of the dietary patterns. Then we examined the association of these pathways with each of the dietary patterns using multivariable adjusted linear regression. In this analysis, we excluded pathways with more than 90% zeros and then conducted a probit transformation before multivariable-adjusted linear regression analyses were used as described above. A cutoff at 0.1 for false discovery rate (FDR)-adjusted *p* value was used to screen significant pathways associated with rEDIH, rEDIP, and HEI-2020.^10^

Additionally, we performed similar multivariable-adjusted linear regression analyses to evaluate the cross-sectional and longitudinal associations between dietary indices and biomarkers measured at visits 1 and 3, respectively (Figure 1a,c). Logistic regression was used to assess the relationship between dietary indices and estimated glomerular filtration rate (eGFR). The eGFR was calculated using CKD-EPI 2021 race-free eGFR equation as recommended by the National Kidney Foundation and the American Society of Nephrology’s Task Force on Reassessing the Inclusion of Race in Diagnosing Kidney Disease.^29^ Subgroup analyses were conducted based on BMI categories: normal weight (18.5 to <25 kg/m^2^) and overweight/obese (25 to 50 kg/m^2^).

The following covariates were included in all multivariable-adjusted models: total energy intake (kcal/day, continuous); age at FFQ time points (years, continuous); self-reported race (White, other); smoking status (never, former/current); number of nutrient supplements used (continuous); educational attainment (high school or less, college or higher); non-steroidal anti-inflammatory drug (NSAID) use (yes/no), aspirin use (yes/no); physical activity (continuous); marital status (married, others), numbers of comorbidities (continuous); and alcohol intake (continuous). We conducted two models: one without BMI and one additionally adjusting for BMI (calculated as weight (kg)/height (m^2^), continuous). Covariate data were collected through self-administered questionnaires on demographics, medical history, and lifestyle at baseline and follow-up Multivariable-adjusted linear and logistic regression analyses on biomarkers, as well as functional pathway analyses, were conducted using SAS® version 9.4 (SAS Institute, Cary, NC) whereas elastic net regression and other microbiome-related analyses were performed in R @version 4.4.0 using Phyloseq and vegan statistical software.

## Results

### Participants’ characteristics

Participants’ characteristics across quintiles of the rEDIH, rEDIP, and HEI-2020 dietary patterns at visit 4 are represented in Table 1. Most participants were white (90%), with an average ( ± SD) age of 84 ± 4 years. Those in the lowest quintile (representing the most hyperinsulinemic, pro-inflammatory, or lowest-quality diets) reported lower intake of magnesium, potassium, folate, vitamin D, and vitamin C, along with higher sodium intake compared with those in the highest quintile of rEDIH or rEDIP, or HEI-2020. Similarly, for macronutrient intake, carbohydrate and fiber consumption were highest, while fat intake was lowest in quintile five compared with quintile 1. Similar trends were observed at the baseline visit (Supplementary Table S1).

**Table 1.** Distribution of participants characteristics in quintiles of the dietary indices at visit 4 (microbiome sub study).

	Reversed Empirical Dietary Index for Hyperinsulinemia (rEDIH)^a,b^					Reversed Empirical Dietary Inflammatory Index (rEDIP)^a,b^					Healthy Eating Index 2020 (HEI-2020)^a,b^				
Characteristic	Quintile 1	Quintile 2	Quintile 3	Quintile 4	Quintile 5	Quintile 1	Quintile 2	Quintile 3	Quintile 4	Quintile 5	Quintile 1	Quintile 2	Quintile 3	Quintile 4	Quintile 5
	( − 3.80, − 0.80)	( − 0.80, − 0.19)	( − 0.18, 0.30)	(0.30, 0.86)	(0.86, 3.67)	( − 5.19, − 0.74)	( − 0.74, − 0.29)	( − 0.29, 0.12)	(0.12, 0.70)	(0.7, 7.35)	( − 3.38,-0.86)	( − 0.86,-0.23)	( − 0.23, 0.31)	(0.31, 0.88)	(0.88, 2.33)
n = 795	159	159	159	159	159	159	159	159	159	159	159	159	159	159	159
Median dietary score^c^	− 1.29	− 0.47	0.04	0.56	1.22	− 1.13	− 0.48	− 0.06	0.37	1.19	59.27	67.58	74.06	79.76	87.67
Age, years, mean ± SD	84 ± 4	84 ± 4	84 ± 4	84 ± 4	85 ± 4	84 ± 4	84 ± 4	85 ± 4	84 ± 4	84 ± 4	84 ± 4	84 ± 4	84 ± 4	84 ± 4	84 ± 4
Race, %															
White, *n* = 711)	87.4	91.2	84.9	95.0	88.7	86.2	88.1	89.9	89.9	93.1	89.9	89.9	89.3	89.9	88.1
Others (*n* = 84)	12.6	8.8	15.1	5.0	11.3	13.8	11.9	10.1	10.1	6.9	10.1	10.1	10.7	10.1	11.9
BMI, kg/m2, %															
Normal weight (18.5≤BMI <25); (*n* = 248)	28.9	27.7	32.1	30.8	36.5	30.2	25.2	34.6	31.4	34.6	25.8	18.2	35.2	34.0	42.8
Overweight (25≤BMI <30); (*n* = 405)	47.2	57.2	52.8	50.9	46.5	48.4	54.1	50.9	52.8	48.4	52.2	56.0	45.9	54.1	46.5
Obese (BMI ≥30); (*n* = 142)	23.9	15.1	15.1	18.2	17.0	21.4	20.8	14.5	15.7	17.0	22.0	25.8	18.9	11.9	10.7
Smoking status; %															
Never smoker (*n* = 409)	44.7	52.2	50.9	54.7	54.7	48.4	45.3	57.2	53.5	52.8	48.4	54.1	52.2	51.6	50.9
Former/Current smoker (*n* = 386)	55.3	47.8	49.1	45.3	45.3	51.6	54.7	42.8	46.5	47.2	51.6	45.9	47.8	48.4	49.1
Marital Status, %															
Married *n* = 562)	71.7	73.0	70.4	69.2	69.2	70.4	67.3	67.9	74.8	73.0	66.0	69.8	67.9	76.1	73.6
others (*n* = 233)	28.3	27.0	29.6	30.8	30.8	29.6	32.7	32.1	25.2	27.0	34.0	30.2	32.1	23.9	26.4
Aspirin use, %															
No (*n* = 336)	39.6	38.4	45.9	39.6	47.8	40.9	36.5	47.8	48.4	37.7	50.3	37.1	40.9	41.5	41.5
Yes (*n* = 459)	60.4	61.6	54.1	60.4	52.2	59.1	63.5	52.2	51.6	62.3	49.7	62.9	59.1	58.5	58.5
NSAIDs use, %															
No (*n* = 688)	86.2	85.5	86.2	87.4	87.4	84.9	84.9	86.2	89.3	87.4	79.2	84.9	88.7	89.3	90.6
Yes (*n* = 107)	13.8	14.5	13.8	12.6	12.6	15.1	15.1	13.8	10.7	12.6	20.8	15.1	11.3	10.7	9.4
Educational level, %															
High school or less (*n* = 326)	35.2	40.3	37.1	39.6	52.8	43.4	37.7	37.7	47.2	39.0	50.9	41.5	44.0	33.3	35.2
college and higher (*n* = 469)	64.8	59.7	62.9	60.4	47.2	56.6	62.3	62.3	52.8	61.0	49.1	58.5	56.0	66.7	64.8
PASE score(means ± SD)	120 ± 67	127 ± 68	123 ± 62	131 ± 64	117 ± 70	132 ± 70	122 ± 66	118 ± 61	119 ± 66	128 ± 66	115 ± 60	121 ± 68	126 ± 68	124 ± 68	132 ± 66
No. of supplements (means ± SD)	9 ± 7	9 ± 7	10 ± 7	9 ± 7	10 ± 7	8 ± 7	9 ± 7	9 ± 7	10 ± 7	10 ± 7	8 ± 7	9 ± 7	8 ± 7	10 ± 7	11 ± 7
No. of comorbidities (means ± SD)	2 ± 1	2 ± 2	2 ± 1	2 ± 1	2 ± 1	2 ± 2	2 ± 1	2 ± 2	2 ± 1	2 ± 1	2 ± 1	2 ± 1	2 ± 2	2 ± 2	2 ± 1
Total macronutrients (mean ± SD), % kcal/d															
Carbohydrates	42 ± 7	45 ± 7	46 ± 7	47 ± 7	49 ± 8	44 ± 7	45 ± 7	47 ± 7	47 ± 7	47 ± 9	43 ± 7	44 ± 7	44 ± 6	47 ± 7	50 ± 7
Proteins	18 ± 3	17 ± 3	16 ± 3	16 ± 3	15 ± 2	17 ± 3	16 ± 3	16 ± 3	16 ± 3	16 ± 3	15 ± 3	16 ± 3	17 ± 3	17 ± 3	17 ± 3
Fats	43 ± 7	41 ± 6	40 ± 7	40 ± 7	40 ± 8	41 ± 7	41 ± 6	40 ± 7	41 ± 7	41 ± 8	44 ± 6	43 ± 6	42 ± 6	39 ± 7	36 ± 7
Fiber (g/d)	10 ± 3	10 ± 3	11 ± 3	11 ± 4	11 ± 4	10 ± 3	10 ± 3	11 ± 3	11 ± 3	13 ± 4	8 ± 2	9 ± 3	11 ± 3	12 ± 3	14 ± 3
Micronutrients (diet only), mean ± SD; units per 1,000 kcal/d															
Calcium, mg	438 ± 144	473 ± 168	488 ± 165	528 ± 236	562 ± 241	422 ± 139	466 ± 155	518 ± 235	526 ± 206	558 ± 216	412 ± 168	448 ± 167	487 ± 185	544 ± 220	598 ± 197
Magnesium, mg	174 ± 35	181 ± 41	189 ± 44	192 ± 45	200 ± 52	163 ± 39	176 ± 36	187 ± 39	194 ± 40	216 ± 50	153 ± 36	168 ± 32	186 ± 35	201 ± 38	229 ± 39
Potassium, mg	1713 ± 291	1793 ± 356	1803 ± 382	1831 ± 425	1826 ± 468	1580 ± 309	1695 ± 05	1799 ± 382	1840 ± 345	2052 ± 433	1505 ± 363	1654 ± 308	1792 ± 326	1936 ± 345	2078 ± 330
Sodium, mg	1530 ± 259	1431 ± 240	1370 ± 272	1434 ± 261	1275 ± 254	1447 ± 240	1422 ± 270	1416 ± 263	1359 ± 253	1396 ± 313	1439 ± 300	1453 ± 293	1448 ± 283	1409 ± 249	1291 ± 172
Phosphorous, mg	704 ± 109	714 ± 125	715 ± 116	738 ± 180	736 ± 160	681 ± 118	707 ± 114	737 ± 178	720 ± 131	761 ± 141	632 ± 120	671 ± 110	730 ± 134	750 ± 126	823 ± 131
Iron, mg	8 ± 3	8 ± 2	7 ± 2	8 ± 2	7 ± 2	7 ± 3	7 ± 2	8 ± 2	8 ± 2	8 ± 2	7 ± 2	7 ± 2	8 ± 2	8 ± 3	8 ± 2
Folate, mg	216 ± 65	220 ± 54	232 ± 61	232 ± 60	240 ± 82	209 ± 68	214 ± 53	223 ± 56	231 ± 54	262 ± 78	185 ± 60	209 ± 49	219 ± 58	251 ± 57	276 ± 60
Vitamin A, RE	825 ± 411	826 ± 386	823 ± 428	897 ± 537	924 ± 639	743 ± 398	709 ± 293	817 ± 381	854 ± 443	1171 ± 697	613 ± 279	755 ± 350	855 ± 413	936 ± 503	1135 ± 655
Vitamin C, mg	59 ± 31	67 ± 37	71 ± 42	68 ± 39	68 ± 42	53 ± 28	61 ± 33	62 ± 34	72 ± 43	85 ± 44	47 ± 28	58 ± 32	61 ± 31	83 ± 45	83 ± 39
Vitamin D, IU	95 ± 56	107 ± 63	108 ± 69	120 ± 97	127 ± 93	104 ± 76	107 ± 66	124 ± 87	112 ± 82	110 ± 78	92 ± 84	102 ± 71	110 ± 77	120 ± 75	133 ± 78
Vitamin E, mg	6 ± 3	6 ± 2	6 ± 2	6 ± 2	6 ± 2	5 ± 3	5 ± 2	6 ± 2	6 ± 2	7 ± 2	6 ± 3	6 ± 2	6 ± 2	6 ± 2	6 ± 2

^a^rEDIH, rEDIP, and HEI2020 scores were adjusted for total energy intake using the residual method. Lower rEDIH indicates more hyperinsulinemic diets and higher rEDIH scores indicates low insulinemic diets. Lower rEDIP indicates proinflammatory diets while higher rEDIP indicates more anti-inflammatory diets. HEI-2020 assessing adherence to the Dietary Guidelines for Americans – higher HEI-2020 scores are indicative of greater adherence and higher dietary quality.

^b^The rEDIH component foods (serving/d) in MrOS were: Processed meat (cold cuts and sausage, lean cold cuts and sausage, cured pork, lean cured pork); high-caloric sugary beverages (sweetened soft drink, sweetened fruit drinks); low-caloric sugary beverages (unsweetened soft drink, artificially sweetened soft drinks, artificially sweetened fruit drinks); cream soup(cream soup); red meat(beef, lean beef, lamb, veal, lean lamb, pork, lean pork); total butter (regular and reduced butter from animals); total margarine(regular and reduced fat margarine); non-dark fish(Lean fish, fried fish); poultry(regular and lean poultry, fried chicken); French fries(fried potato); tomato; low-fat dairy (Low-fat cream, low-fat yogurt, sweetened low fat yogurt, artificially sweetened low-fat yogurt, fat-free yogurt, artificially sweetened fat-free yogurt, low-fat cheese, low fat milk, ready to drink flavored reduced fat milk, artificially sweetened nonfat milk, sweetened and artificially sweetened dry milk); eggs; wine; coffee(sweetened, unsweetened, and artificially sweetened coffee); whole fruit(citrus fruit, fruit excluding citrus fruit, fried fruit, avocado and similar); full-fat dairy (full-fat cream, full-fat cheese, full-fat yogurt, sweetened and artificially sweetened full-fat milk, flavored full-fat milk, dairy dessert, frozen dessert, pudding and others); green-leafy vegetables(dark-green vegetables, vegetables juice). The rEDIP component foods (serving/d) in MrOS were: Processed meat (cold cuts and sausage, lean cold cuts and sausage, cured pork, lean cured pork); red meat(beef, lean beef, lamb, veal, lean lamb, pork, lean pork); organ meats, non-dark fish(Lean fish-fresh and smoked, fried fish -commercial entree and fast food), other vegetables(other starchy vegetables, other vegetables, fried vegetables); refined grains(refined grains, flour, and dry mixes, loaf-type bread and plain rolls, quick breads, corn muffins, tortillas, pasta, presweetened and not presweetened ready-to-eat cereal, cakes, cookies, pies, pastries, Danish, doughnuts, cobblers, snack bars, baby food grain mixtures, snacks chips, crackers); high-caloric sugary beverages (sweetened soft drink, sweetened fruit drinks); low-caloric sugary beverages (unsweetened soft drink, artificially sweetened soft drinks, artificially sweetened fruit drinks), tomato, beer; wine; tea(sweetened tea, artificially sweetened tea, unsweetened tea); coffee(sweetened coffee, artificially sweetened coffee, unsweetened coffee); deep-yellow vegetables; green leafy vegetables (dark-green vegetables, vegetable soup); snacks(vegetable-based savory snacks, fruit-based savory snack, whole-grain snack chip, some whole-grain snack chips, refined-grain snack chips, popcorn, flavored popcorn, whole-grain crackers, some whole-grain cracker, refined grain cracker); fruit juice (citrus juice, fruit juice excluding citrus juice); pizza The HEI2020 component foods (servings/d) in the MrOS were: total fruits, whole fruits, total vegetables, greens and beans, whole grains, dairy, total protein foods, sea food and plant proteins, fatty acids, refined grains, sodium, added sugars, saturated fats.

^c^Median score for HEI_2020 is raw median scores not adjusted for total energy.

Note: Values are presented as mean ± SD for continuous variables and percentage for categorical variables.

### Dietary indices in relation to microbiota alpha diversity

In cross-sectional analyses at visit 4, higher rEDIH and rEDIP, reflecting lower insulinemic or more anti-inflammatory dietary patterns, were associated with higher microbiota alpha diversity, as the inverse Simpson index increased by 4.9% for both rEDIH and rEDIP and by 7.8% for HEI-2020 per 1-SD increase in dietary scores (Figure 2). Similarly, Pielou’s Evenness index decreased by 0.8% and 1.2% for the same comparisons. For both rEDIP and rEDIH, the associations were slightly attenuated after additional adjustment for BMI. No significant associations were observed after the dietary scores were categorized into quintiles. Similar results were observed for longitudinal assessments of alpha diversity with rEDIH, rEDIP, and HEI-2020 scores at visit 1, with Shannon diversity also becoming significant in this analysis (Supplementary Table S2). However, no significant association was observed between changes in dietary scores and alpha diversity (Figure 2, Supplementary Table S2). In sensitivity analyses, we further adjusted models for proton pump inhibitor and antidiabetic medication use and the results remained consistent with the primary analyses (data not shown).

### Dietary patterns-related microbiome profile scores

**Figure 3.**
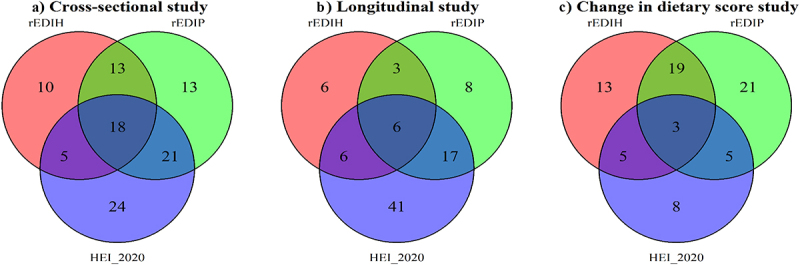
Venn diagrams showing the number of microbial genera identified from elastic net regression analyses, across the three dietary patterns in a) cross-sectional, b) longitudinal, and c) change in dietary score analyses. reversed empirical dietary index for hyperinsulinemia (rEDIH), reversed empirical dietary index for inflammation (rEDIP), and the healthy eating index-2020 (HEI_2020).

**Figure 4.**
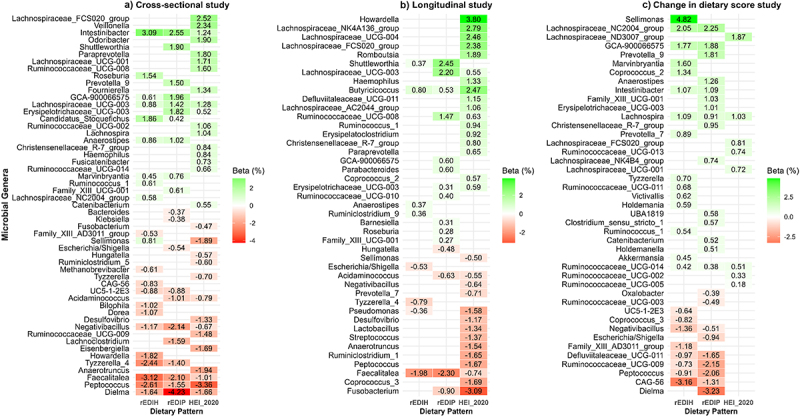
Microbial genera selected by elastic net regression in a) cross-sectional, b) longitudinal, and c) change-in-dietary-score analyses, aggregated at the genus level for each of the three dietary patterns (rEDIH, rEDIP, and HEI-2020). Each number within the heatmap represents the beta coefficient (expressed as a percentage), indicating the strength of the association between a specific microbial genus and the dietary pattern. Green shades indicate genera associated with healthier diets, while red/brown shades represent genera linked to more hyperinsulinemic, pro-inflammatory, and lower-quality diets. In addition, the genera presented in the figures represent the top “hits” representing 90% of the variance in the total number of genera associated with the dietary pattern. The cross-sectional analysis represents the primary analysis.

Dietary index-related microbiome scores at the genus level were developed using data from visit 4 (cross-sectional), visit 1 (longitudinal), and the change in dietary scores (difference between visits 4 and 1 scores). For the cross-sectional analysis, elastic net regression retained 46, 65, and 68 genera to compute the rEDIH, rEDIP, and HEI-2020-related microbiome scores, respectively (Figures 3, Supplementary Table S3). For rEDIH, the genera *Faecalitalea* (β = − 0.031), *Peptococcus* (β = − 0.026), and *Tyzzerella*_4 (β = − 0.024) showed the strongest inverse associations (indicating a proinsulinemic profile), while *Intestinibacter* (β = 0.031), *Candidatus_Stoquefichus* (β = 0.018), and *Roseburia* (β = 0.015) had the largest positive associations (indicating a low insulinemic profile) (Figures 4, Supplementary Table S3). For rEDIP, *Dielma* (β = − 0.042), *Negativibacillus* (β = − 0.021), and *Faecalitalea* (β = − 0.021) showed the most significant inverse associations (indicating a proinflammatory profile), while *Intestinibacter* (β = 0.025), *Shuttleworthia* (β = 0.019), and *Erysipelotrichaceae*_*UCG-003* (β = 0.018) had the largest positive associations (Figure 4, Supplementary Table S3). For HEI-2020, *Peptococcus* (β = − 0.033), *Anaerotruncus* (β = − 0.019), and *Sellimonas* (β = − 0.019) exhibited the strongest inverse associations (suggesting a low-quality diet), while *Lachnospiraceae*_*FCS020*_*group* (β = 0.025), *Veillonella* (β = 0.023), and *Odoribacter* (β = 0.019) showed the strongest positive associations (indicating a high-quality diet). Interestingly, a few genera were consistently associated with all three dietary indices (Figure 4, Supplementary Table S3)). Eight genera, including *Negativibacillus*, *Dielma*, *Dorea*, *Bilophila*, *UC5–1-2E3*, *Tyzzerella_4*, *Peptococcus*, and *Faecalitalea*, were inversely associated across the three dietary indices, while five genera, including *Intestinibacter*, *Lachnospiraceae*_UCG-003, *Lachnospira*, *Ruminiclostridium_9*, and *Haemophilus*, showed positive associations. Findings in the cross-sectional analyses were similar in the longitudinal analyses (Supplementary Table S4). However, in the analyses evaluating the changes in the dietary patterns between baseline and visit 4, these common genera tended to define rEDIP and rEDIH (Supplementary Tables S5).

### Microbiome functional analysis

In the predicted pathways analysis, cross-sectional multivariable-adjusted linear regression identified 15 pathways inversely associated with rEDIH, (FDR p-value <0.1). These associations were slightly attenuated after additional adjustment for BMI (Supplementary Table S6). The NAD salvage pathway I, was the most strongly inversely associated with rEDIH. Similarly, for rEDIP, 22 pathways were inversely associated. The pathway related to glucose and mannose degradation, and the NAD salvage pathway I were the major pathways inversely associated with rEDIP. For HEI-2020, 48 pathways were statistically significant. A higher HEI score was associated with the downregulation of pathways, including superpathway of N-acetylglucosamine, NAD salvage pathway I, and glycogen degradation I, a pathway involved in breaking down glucose into pyruvate (Supplementary Table S6). Longitudinal analysis similarly identified 19 pathways for rEDIH, 13 for rEDIP, and 21 for HEI-2020. However, no pathways remained significant after FDR adjustment in the analysis of changes in dietary scores.

### Dietary index scores and circulating biomarker concentrations

The association of eGFR with the dietary patterns is shown in Table 2. Individuals with higher rEDIH and rEDIP scores had lower odds of reduced eGFR compared with those with the lowest scores. For a 1-SD increase in rEDIH, the odds ratios (OR) were 0.88 (95% confidence interval [CI]: 0.80, 0.97) in the multivariable (MV) model and 0.90 (95% CI: 0.82, 1.00) in the MV + BMI model, indicating a 12% and 10% reduction in the odds of reduced eGFR, respectively. In the longitudinal analysis, higher dietary quality, reflected by higher rEDIH and rEDIP scores, was similarly associated with a reduced risk of poor kidney function (eGFR <60) (Table 2). Specifically, those in the highest rEDIH quintile had a 60% lower odds of reduced kidney function (OR = 0.40) in the MV model and 58% lower odds (OR = 0.42) in the MV + BMI model, compared with those in the lowest quintile. Although the HEI-2020 score also suggested a protective effect, its association was weaker and did not reach statistical significance in the cross-sectional study but was significant in the longitudinal study.

**Table 2.** Odds ratios for the cross-sectional associations of dietary pattern scores with estimated glomerular filtration rate^a^.

Dietary score	Statistical model	Lowest dietary quality		Dietary score Quintiles (Q)		Highest dietary quality		OR per 1 sd increment in dietary score	P-value
		Quintile 1	Quintile 2	Quintile 3	Quintile 4	Quintile 5	P-trend^d^		
rEDIH^e^	n(case/control)							496/3925	
	MV model^b^	1(reference)	1.15(0.85,1.57)	0.98(0.72,1.34)	0.89(0.65,1.23)	0.72(0.51,0.99)	0.0158	0.88(0.80,0.97)	0.0127
	MV + BMI^c^	1(reference)	1.20(0.88,0.63)	1.03(0.75,1.41)	0.94(0.68,1.30)	0.77(0.55,1.08)	0.0601	0.90(0.82,1.00)	0.0452
rEDIP^e^	n(case/control)							496/3925	
	MV model^b^	1(reference)	1.22(0.91,1.64)	0.81(0.59,1.12)	0.95(0.61,1.32)	0.91(0.65,1.26)	0.1923	0.88(0.79,0.98)	0.0296
	MV + BMI^c^	1(reference)	1.24(0.92,1.66)	0.84(0.60,1.15)	0.97 (0.70,1.35)	0.93(0.67,1.20)	0.1294	0.90(0.81,1.00)	0.0538
HEI-2020^e^	n(case/control)							496/3925	
	MV model^b^	1(reference)	0.90(0.66,1.23)	0.95(0.70,1.30)	0.82(0.60,1.13)	0.89(0.65,1.23)	0.4903	0.95(0.85,1.05)	0.3043
	MV + BMI^c^	1(reference)	0.89(0.65,1.21)	0.94(0.68,1.29)	0.83(0.60,1.14)	0.94(0.67,1.28)	0.3318	0.96(0.86,1.06)	0.4256
**Odds ratios for the longitudinal associations of dietary pattern scores with estimated glomerular filtration rate**									
rEDIH^e^	n(case/control)							131/560	
	MV model^b^	1(reference)	0.85(0.47,1.58)	0.63(0.33,1.20)	0.61(0.32,1.16)	0.40(0.20,0.81)	0.0053	0.74(0.60,0.90)	0.0039
	MV + BMI^c^	1(reference)	0.87(0.47,1.60)	0.65(0.34,1.23)	0.62(0.33,1.19)	0.42(0.21, 0.84)	0.0092	0.75(0.61,0.92)	0.0062
rEDIP^e^	n(case/control)							131/560	
	MV model^b^	1(reference)	0.61(0.31,1.19)	0.93(0.50,1.76)	0.80(0.41,1.52)	0.39(0.19,0.82)	0.0360	0.77(0.61,0.96)	0.0190
	MV + BMI^c^	1(reference)	0.61(0.32, 1.18)	0.95(0.51,1.80)	0.82(0.43,1.58)	0.40(0.19,0.85)	0.0482	0.76(0.62,0.97)	0.0253
HEI-2020^e^	n(case/control)							131/560	
	MV model^b^	1(reference)	1.00(0.53,1.89)	0.95(0.50,1.81)	0.79(0.41,1.51)	0.52(0.25,1.05)	0.0521	0.78(0.63,0.97)	0.0250
	MV + BMI^c^	1(reference)	0.99(0.52,1.87)	0.94(0.50,1.79)	0.78(0.41,1.51)	0.53(0.26,1.08)	0.0666	0.79(0.64,0.98)	0.0309

A case was defined as eGFR <60 mL/min/1.73 m^2^. The eGFR was calculated using CKD-EPI 2021 race-free eGFR equation.

^a^Values presented are odd ratio (OR) and 95% confidence intervals (95% CI).

^b^ORs were derived from MV (multivariable) adjusted, ^c^multivariable + BMI (body mass index) adjusted logistic regression models.

Multivariable (MVs) logistic regression models adjusted for total energy intake, race/ethnicity (White, African American, Asian, Hispanic, others), marital status (married, single, divorced/separated, widowed), education (less than high school, high school, college and higher), physical activity (continuous), smoking status (never, past, current), pack-years of smoking (continuous), number of nutritional supplements used (continuous), total alcohol intake (continuous, servings/day), aspirin/other NSAID use (yes vs no), baseline comorbidity score conditions (continuous).

^d^The *p* value for linear trend was estimated in the same models by assigning the quintile-specific median value of each dietary pattern to all participants in the quintile and modeling as an ordinal variable.

^e^rEDIH, reversed Empirical Dietary Index for Hyperinsulinemia; rEDIP, reversed Empirical Dietary Inflammatory Pattern, HEI-2020, Healthy Eating Index-2020.

**Figure 5.**
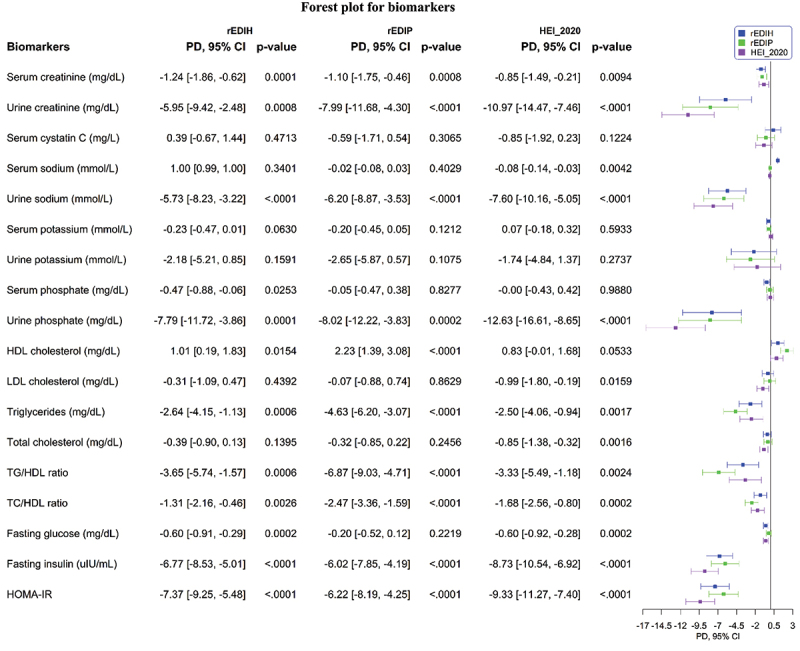
Associations of dietary patterns (per 1 standard deviation increment) with biomarkers of renal functions, lipids, and insulin response. Values are absolute back-transformed biomarker concentrations (beta coefficients). Beta coefficients were derived from MV (multivariable) adjusted linear regression models. MV models were adjusted for total energy intake, BMI-continuous, age, physical activity, smoking status, number of supplement used, sex, race/ethnic groups, education levels, use of NSAIDS, marital status, alcohol drinking status, family history of cancer, and diabetes status. HDL, high-density lipoprotein; LDL, low-density lipoprotein; TC, total cholesterol; HOMA-IR, homeostatic model assessment of insulin resistance.

In the analysis of associations between chronic disease biomarkers and each dietary pattern, we observed that higher rEDIH, rEDIP, and HEI-2020 scores were significantly associated with lower concentrations of serum creatinine, urine creatinine, serum sodium, urine phosphate, serum LDL-C, triglycerides, and total cholesterol in cross-sectional analyses (Figure 5, Supplementary Tables S7-S9). In longitudinal analyses, serum creatinine and serum cystatin were significantly associated with rEDIH and rEDIP, but not with HEI-2020. Additionally, in the longitudinal study, the associations with insulin response biomarkers for all dietary patterns were attenuated. (Supplementary Table S10).

### Biomarker analysis in subgroups defined by BMI status

Subgroup analysis by BMI status showed that individuals with normal weight experienced a greater reduction in serum creatinine, serum potassium, LDL-C, and total cholesterol with each 1-SD increase in the rEDIH dietary score, compared with those with higher BMI (overweight/obese). However, the associations for rEDIP and HEI-2020 did not differ significantly based on BMI status (Supplementary Table S11).

## Discussion

This study provides novel insights into the relationship between inflammatory and insulinemic dietary patterns, and overall dietary quality, gut microbiota composition, and chronic disease biomarkers in older American men. Findings showed that higher adherence to low-insulinemic (rEDIH) and anti-inflammatory (rEDIP) dietary patterns, is significantly associated with favorable profiles of gut microbiota diversity and lower levels of biomarkers linked to impaired renal function, dyslipidemia, and insulin resistance. Given that our dietary patterns specifically focus on inflammation and insulin secretion – both key factors in chronic disease risk – this study also explored microbiome functional analysis. We found that the microbes associated with these dietary patterns were linked to key metabolic pathways, such as cellular energy metabolism, highlighting potential microbiome-mediated mechanisms through which diet influences chronic disease processes. These findings reinforce the importance of dietary strategies that reduce inflammation and insulin hypersecretion, supporting current dietary recommendations for chronic disease prevention. By identifying microbiome-related pathways influenced by diet, our study also provides a foundation for future research aimed at developing targeted nutritional interventions to improve metabolic health via enhanced gut microbiome composition and function.

The significant positive associations between rEDIH and rEDIP with gut microbiota alpha diversity, underscore the potential role of these dietary patterns in fostering a healthy gut microbiome. Higher diversity of gut microbiota has been consistently linked to better metabolic health, enhancement of immune function, and reduced risk of chronic diseases, including inflammatory bowel disease, diabetes, and cardiovascular disease.^30,31^ Current study findings, along with findings from the Shi *et al* study on the same metabolically-derived dietary patterns conducted in the TwinsUK cohort,^10^ align with previous research suggesting that diets rich in anti-inflammatory and insulin-sensitizing foods may foster microbial diversity, which may help mitigate the risk of chronic diseases. Both studies observed inverse associations between rEDIH and genera such as *Faecalitalea*, *Negativibacillus*, *Turicibacter*, UC5–1-2E3, *Parasutterella*, and *Lactobacillus*, while *Marvinbryantia* and *Fournierella* were positively associated. Similarly, for rEDIP, *Escherichia*/*Shigella* and *Negativibacillus* showed inverse associations, while *Ruminococcaceae_UCG-014* and *Ruminococcaceae_UCG-008* were positively associated, reflecting a consistent microbiota profile linked to these dietary patterns in different study populations and geographic regions. Previous studies have found that changes in microbial composition are related to shifts in metabolites that influence hypoglycemic effects, immune function, and inflammatory activity.^32,33^ In line with this finding, the identification of specific genera such as *Intestinibacter* and *Lachnospira*, positively associated with rEDIH and rEDIP, highlights the potential for targeted dietary interventions to influence gut microbial composition and improved metabolic health. In contrast, genera such as *Tyzzerella*, *Negativibacillus*, and *Coprococcus* were enriched in individuals at higher risk of cardiovascular disease, while *Ruminococcus* was more prevalent in those at lower risk,^34^ aligning with our results showing inverse associations between these microbes and higher quality diets. In addition, previous studies showed that a higher pro-inflammatory dietary pattern was associated with greater risk of colorectal tumors enriched with *Fusobacterium nucleatum*,^35^ consistent with current study findings. These findings shed light on a potential mechanism for how diet-related inflammation may influence colorectal tumor development and progression. A previous study using the same dataset of 517 men found no association between alpha diversity and dietary patterns, specifically the Western and Prudent patterns, which contrasts with the findings of the current study.^9^ The differences in results may stem from methodological and analytical advancements in our study. We used a priori dietary scores, offering a standardized approach, while the previous study relied on population-specific data-driven patterns. Our use of Inverse Simpson diversity, which highlights dominant taxa, and the SILVA v128 database with ASVs (vs. OTUs with Greengenes) improved taxonomic resolution. Additionally, robust statistical models and advanced pipelines, including DADA2 error correction, likely increased sensitivity to detect associations.

The microbiome functional analysis using PICRUSt2 identified several pathways inversely associated with rEDIH and rEDIP related microbial profiles, such as NAD salvage pathway I, glycogen degradation, and glucose and mannose degradation pathways. These pathways are essential for cellular energy metabolism and nucleotide biosynthesis, suggesting that rEDIP and rEDIH may help reduce metabolic stress by modulating these processes. Although microbiota varies between individuals, studies have shown that functional gene repertoires exhibit great similarity among adults.^6^ These findings highlight the role of diet-induced changes in microbiome functionality, particularly in key pathways like glycolysis, which underscores its central importance in energy metabolism.

The observed associations between rEDIH and rEDIP scores and lower concentrations of chronic disease biomarkers highlight the potential of these dietary patterns to improve metabolic health, including reduced risk for type 2 diabetes and cardiovascular diseases.^16,36,37^ Also, these findings are consistent with previous studies linking anti-inflammatory and insulin-sensitizing diets to improve renal function and lipid profiles.^17,38^ The lack of long-term associations with insulin response biomarkers may reflect the complexity of insulin regulation and the multifactorial influences that extend beyond diet, such as genetic factors, physical activity, and hormonal changes associated with aging. Notably, our subgroup analysis by BMI showed that the beneficial effects of these dietary patterns were more pronounced in individuals with normal weight compared with those with higher BMI, suggesting that the effectiveness of such diets may be influenced by baseline BMI. In overweight or obese individuals, insulin resistance and chronic inflammation may blunt the beneficial effects of diet on biomarker levels, necessitating more comprehensive lifestyle interventions. However, the underlying mechanisms behind these benefits may be mediated, in part, through interactions with the gut microbiome. Anti-inflammatory and insulin-sensitizing diets, like those represented by rEDIP and rEDIH, foster the growth of several members of *Lachnospiraceae*, including *Blautia* and *Roseburia* which were identified in our study. These bacteria produce short-chain fatty acids (SCFAs), such as butyrate, acetate, and propionate, which play key roles in maintaining gut health.^39^ These SCFAs strengthen the gut barrier, reduce systemic inflammation, enhance insulin sensitivity, and support lipid metabolism, thereby protecting kidney function, preventing dysbiosis, and improving lipid profiles.^4,39^ Additionally, a study showed that *Lachnospiraceae* were most strongly associated with response to immunotherapy.^40^ These findings emphasize the role of dietary interventions in the prevention and management of chronic diseases and highlight the gut microbiome as a key mediator of these effects. To further validate these results, additional studies and clinical trials are necessary to confirm and expand upon these findings.

This study has several strengths, including its large, well-characterized study sample, comprehensive dietary assessments, and the incorporation of both cross-sectional and longitudinal analyses. The inclusion of gut microbiome functional analysis and biomarkers adds novelty to our understanding of how diet influences metabolic health through microbial pathways. However, there are limitations to consider. One specific limitation is the use of 16S sequencing with older reference data (SILVA v128), as taxonomic names and phylogeny of the gut microbiome are continually evolving. While SILVA remains a widely used reference, this may limit future comparisons with studies adopting newer versions or alternative databases. Residual confounding cannot be ruled out despite adjustment for many potential confounding variables. In particular, data on fecal consistency – such as Bristol Stool Chart scores, which are known to influence gut microbiota composition, were not available in the MrOS microbiome dataset. The use of self-reported dietary data through FFQs may introduce reporting bias and lack nuance in capturing specific macronutrient details (e.g., saturated vs. unsaturated fats). Further, because our study population included mainly older White men, the generalizability of our findings to women, younger adults, or other racial/ethnic groups is limited. Future intervention studies are needed to confirm these associations and to further explore the long-term effects of dietary patterns on gut microbiota and chronic disease outcomes.

## Conclusion

In conclusion, our study highlights the significant associations between inflammatory and insulinemic dietary patterns and gut microbiota diversity, functional pathways, and biomarkers of chronic diseases in older American men. These findings underscore the high potential for future dietary interventions targeting inflammation and insulin regulation to improve metabolic health and reduce chronic disease risk. In addition, future research should investigate more precise mechanisms underlying these associations, with particular attention to the long-term effects of dietary patterns on health outcomes in diverse populations.

## Supplementary Material

Supplementary_table_word_3_25_2025.docx

## Data Availability

The data used in this study are publicly available from the MrOS (Osteoporotic Fractures in Men) Study website - https://mrosonline.ucsf.edu. The website includes detailed information about the study, its datasets, guidance on proper use, and publication guidelines to ensure the integrity and reproducibility of research using this data.
