# Supplementary material for: Role of insulinemic and inflammatory dietary patterns on gut microbial composition and circulating biomarkers of metabolic health among older American men
Source: Gut Microbes. 2025 Apr 28;17(1):2497400. doi: 10.1080/19490976.2025.2497400 (PMC12045561; doi:10.1080/19490976.2025.2497400)
Supplement: Supplementary_table_word_3_25_2025.docx [file KGMI_A_2497400_SM8166.docx]

| **Supplementary Table 1. Distribution of participants baseline characteristics in quintiles of the dietary indices.** | | | | | | | | | | | | | | |  |
| --- | --- | --- | --- | --- | --- | --- | --- | --- | --- | --- | --- | --- | --- | --- | --- |
|  | **Reversed Empirical Dietary Index for  Hyperinsulinemia (rEDIH)^1,2^** | | | | | **Reversed Empirical Dietary Inflammatory Index   (rEDIP)^1,2^** | | | | | **Healthy Eating Index 2020 (HEI-2020) ^1,2^** | | | | |
| **Characteristic** | Q1 | Q 2 | Q3 | Q4 | Q5 | Q1 | Q 2 | Q3 | Q4 | Q5 | Q1 | Q 2 | Q3 | Q4 | Q5 |
|  | (-8.66,-0.65) | (-0.65,-0.12) | (-0.12, 0.27) | (0.27, 0.73) | (0.73, 4.44) | (-5.45,-0.73) | (-0.73, -0.28) | (-0.28, 0.15) | (0.15, 0.69) | (0.69, 7.30) | (-3.7,  -0.88) | (-0.88,  -0.21) | (-0.21,  0.34) | (0.34,  0.92) | (0.92,  2.35) |
| n = 4771 | 954 | 954 | 955 | 954 | 954 | 954 | 954 | 955 | 954 | 954 | 954 | 954 | 955 | 954 | 954 |
| Median dietary score^3^ | -1.16 | -0.36 | 0.07 | 0.47 | 1.11 | -1.1 | -0.48 | -0.07 | 0.40 | 1.19 | 49.89 | 57.86 | 63.91 | 70.02 | 77.37 |
| Age, years, means ± SD | 72±5 | 73±6 | 74±6 | 74±6 | 74±6 | 73±6 | 73±6 | 73±6 | 73±5 | 73±6 | 73±6 | 73±6 | 73±6 | 73±6 | 74±6 |
| Race, % |  |  |  |  |  |  |  |  |  |  |  |  |  |  |  |
| White (n=4295) | 90.7 | 88.4 | 88.3 | 91.0 | 91.8 | 83.4 | 88.0 | 91.5 | 93.0 | 94.2 | 91.0 | 90.7 | 89.2 | 89.5 | 89.7 |
| African American (n=180) | 3.8 | 5.0 | 4.9 | 2.7 | 2.4 | 6.9 | 5.6 | 2.9 | 1.5 | 2.0 | 4.2 | 4.6 | 3.6 | 3.8 | 2.7 |
| Asian (n=149) | 2.4 | 3.0 | 4.0 | 3.6 | 2.6 | 5.5 | 3.7 | 3.4 | 1.9 | 1.3 | 1.4 | 2.2 | 3.8 | 3.3 | 5.0 |
| Others  (n=147) | 3.1 | 3.6 | 2.8 | 2.7 | 3.1 | 4.2 | 2.8 | 2.2 | 3.7 | 2.5 | 3.5 | 2.5 | 3.5 | 3.5 | 2.5 |
| BMI, kg/m2, mean ± SD | 29±4 | 28±4 | 27±4 | 27±4 | 26±3 | 28±4 | 28±4 | 27±4 | 27±4 | 27±4 | 28±4 | 28±4 | 28±4 | 27±4 | 26±3 |
| Normal weight   (18.5≤BMI< 25); % (n=1315) | 18.6 | 23.8 | 28.0 | 30.4 | 37.1 | 24.0 | 24.8 | 28.5 | 29.8 | 30.7 | 23.4 | 23.5 | 23.7 | 29.3 | 38.1 |
| Overweight  (25≤BMI < 30); % (n=2449) | 48.5 | 51.4 | 53.9 | 52.3 | 50.5 | 48.9 | 52.9 | 50.8 | 52.8 | 51.3 | 49.8 | 52.0 | 53.4 | 52.9 | 48.5 |
| Obese (BMI ≥30); % (n=1003) | 32.9 | 24.8 | 17.9 | 17.2 | 12.3 | 27.2 | 22.1 | 20.7 | 17.3 | 17.8 | 26.7 | 24.5 | 22.9 | 17.7 | 13.2 |
| Pack years of smoking^4^  means ± SD | 23±28 | 18±23 | 17±24 | 16±22 | 17±24 | 22±28 | 16±23 | 16±23 | 17±24 | 19±24 | 26±30 | 19±25 | 18±24 | 15±21 | 12±19 |
| Smoking status; % |  |  |  |  |  |  |  |  |  |  |  |  |  |  |  |
| Never smoker (n=1783) | 33.3 | 35.1 | 39.0 | 39.7 | 39.7 | 35.5 | 40.2 | 39.3 | 37.8 | 34.1 | 30.8 | 35.7 | 34.4 | 39.4 | 46.5 |
| Former/Current smoker (n=2988) | 66.7 | 64.9 | 61.1 | 60.3 | 60.3 | 64.5 | 59.9 | 60.7 | 62.2 | 65.9 | 69.2 | 64.3 | 65.7 | 60.6 | 53.5 |
| Marital Status, % |  |  |  |  |  |  |  |  |  |  |  |  |  |  |  |
| Married  (n=3952) | 82.6 | 82.7 | 85.6 | 82.9 | 80.4 | 79.8 | 82.5 | 85.1 | 84.8 | 82.0 | 80.7 | 81.9 | 84.7 | 84.6 | 82.3 |
| others (n=819) | 17.4 | 17.3 | 14.5 | 17.1 | 19.6 | 20.2 | 17.5 | 14.9 | 15.2 | 18.0 | 19.3 | 18.1 | 15.3 | 15.4 | 17.7 |
| Aspirin use, % |  |  |  |  |  |  |  |  |  |  |  |  |  |  |  |
| No (n=3730) | 78.5 | 79.1 | 78.5 | 77.9 | 76.8 | 77.0 | 76.4 | 78.0 | 79.4 | 80.1 | 81.0 | 80.0 | 78.1 | 76.9 | 74.8 |
| Yes (n=1041) | 21.5 | 20.9 | 21.5 | 22.1 | 23.2 | 23.0 | 23.6 | 22.0 | 20.7 | 19.9 | 19.0 | 20.0 | 21.9 | 23.1 | 25.2 |
| NSAIDs use, % |  |  |  |  |  |  |  |  |  |  |  |  |  |  |  |
| No  (n=4409) | 92.1 | 92.1 | 92.3 | 93.1 | 92.5 | 92.7 | 91.8 | 92.4 | 93.3 | 91.9 | 90.8 | 92.1 | 91.1 | 93.0 | 95.1 |
| Yes  (n=362) | 7.9 | 7.9 | 7.8 | 6.9 | 7.6 | 7.3 | 8.2 | 7.6 | 6.7 | 8.1 | 9.2 | 7.9 | 8.9 | 7.0 | 4.9 |
| Educational level, % | |  |  |  |  |  |  |  |  |  |  |  |  |  |  |
| High school or less (n= 2240) | 55.6 | 51.1 | 45.2 | 42.2 | 40.7 | 60.3 | 49.2 | 46.0 | 39.4 | 39.9 | 61.0 | 51.8 | 45.8 | 41.2 | 35.0 |
| college and higher (n=2531) | 44.4 | 49.0 | 54.8 | 57.8 | 59.3 | 39.7 | 50.8 | 54.0 | 60.6 | 60.1 | 39.0 | 48.2 | 54.2 | 58.8 | 65.0 |
| PASE score (means ±SD) | 148±66 | 145±68 | 144±67 | 150±69 | 152±72 | 143±69 | 148±69 | 147±64 | 149±67 | 153±73 | 142±69 | 146±66 | 147±70 | 152±71 | 151±67 |
| No. of supplements (means ± SD) | 10±8 | 10±8 | 10±8 | 11±8 | 11±7 | 9±8 | 10±8 | 10±8 | 11±8 | 11±7 | 9±8 | 10±8 | 11±8 | 10±8 | 12±7 |
| No. of comorbidities (means ± SD) | 2±2 | 2±1 | 2±2 | 2±1 | 2±1 | 2±2 | 2±2 | 2±2 | 2±1 | 2±1 | 2±1 | 2±2 | 2±1 | 2±2 | 2±1 |
| Total macronutrients (mean ±SD), % kcal/d | | | |  |  |  |  |  |  |  |  |  |  |  |  |
| Carbohydrates | 45±7 | 49±7 | 51±8 | 53±8 | 53±9 | 49±9 | 50±8 | 50±8 | 51±9 | 51±9 | 44±8 | 48±8 | 50±8 | 52±7 | 56±7 |
| Proteins | 18±3 | 17±3 | 16±2 | 15±2 | 15±2 | 17±3 | 16±3 | 16±3 | 16±3 | 16±3 | 15±3 | 16±3 | 16±3 | 16±3 | 17±3 |
| Fats | 39±7 | 37±7 | 36±8 | 35±8 | 36±9 | 37±8 | 37±8 | 36±8 | 36±8 | 36±9 | 42±7 | 39±7 | 37±7 | 35±7 | 31±7 |
| Fiber (g/d) | 17±7 | 16±7 | 16±7 | 18±8 | 24±10 | 17±8 | 16±7 | 17±8 | 18±8 | 23±10 | 13±6 | 16±7 | 18±8 | 20±8 | 23±10 |
| Micronutrients (diet only), mean ± SD; units per 1,000 kcal/d | | | |  |  |  |  |  |  |  |  |  |  |  |  |
| Calcium, mg | 426±150 | 489±188 | 527±204 | 557±228 | 546±212 | 429±151 | 502±200 | 525±204 | 541±219 | 548±216 | 428±178 | 467±180 | 497±192 | 541±199 | 612±217 |
| Magnesium, mg | 159±32 | 175±36 | 183±38 | 197±42 | 207±52 | 157±37 | 174±38 | 186±37 | 195±39 | 211±48 | 149±35 | 169±33 | 182±34 | 197±36 | 225±41 |
| Potassium, mg | 1680±342 | 1785±368 | 1842±395 | 1927±422 | 1958±496 | 1563±343 | 1730±349 | 1842±351 | 1953±386 | 2103±446 | 1534±364 | 1722±351 | 1819±365 | 1953±360 | 2165±370 |
| Sodium, mg | 1449±260 | 1433±246 | 1428±257 | 1370±230 | 1339±258 | 1412±258 | 1388±214 | 1396±228 | 1392±248 | 1432±308 | 1464±308 | 1431±250 | 1407±254 | 1378±229 | 1340±199 |
| Phosphorous, mg | 692±124 | 711±139 | 723±147 | 750±162 | 735±152 | 681±125 | 720±153 | 727±147 | 736±149 | 746±149 | 642±130 | 680±126 | 717±131 | 749±137 | 823±140 |
| Iron, mg | 8±2 | 8±3 | 8±3 | 8±3 | 8±3 | 8±3 | 8±3 | 8±3 | 8±3 | 9±3 | 7±3 | 8±3 | 8±3 | 8±3 | 9±3 |
| Folate, mg | 206±59 | 231±65 | 247±81 | 255±79 | 266±91 | 211±73 | 227±66 | 243±78 | 250±74 | 274±86 | 194±57 | 223±65 | 236±71 | 259±77 | 292±82 |
| Vitamin A, RE | 757±350 | 825±392 | 851±391 | 905±522 | 999±685 | 687±346 | 770±340 | 829±385 | 896±429 | 1154±714 | 646±303 | 766±363 | 849±428 | 955±505 | 1120±641 |
| Vitamin C, mg | 63±32 | 74±38 | 83±43 | 84±43 | 89±49 | 57±32 | 69±37 | 78±38 | 88±44 | 101±46 | 51±31 | 71±38 | 78±40 | 91±43 | 102±41 |
| Vitamin D, IU | 90±53 | 104±71 | 107±73 | 120±84 | 112±79 | 99±63 | 113±78 | 109±79 | 107±70 | 105±75 | 93±70 | 102±73 | 99±68 | 110±72 | 129±78 |
| Vitamin E, mg | 5±3 | 6±3 | 6±4 | 6±3 | 7±3 | 5±3 | 5±3 | 6±4 | 6±3 | 7±3 | 5±2 | 6±3 | 6±3 | 6±4 | 6±4 |

| 1. rEDIH, rEDIP, and HEI2020 scores were adjusted for total energy intake using the residual method. Lower rEDIH indicates more hyperinsulinemic diets and higher rEDIH scores indicates low insulinemic diets. Lower rEDIP indicates proinflammatory diets while higher rEDIP indicates more anti-inflammatory diets. HEI-2020 assessing adherence to the Dietary Guidelines for Americans - higher HEI-2020 scores are indicative of greater adherence and higher dietary quality.  2. The rEDIH component foods (serving/d) in MrOS were: Processed meat (cold cuts and sausage, lean cold cuts and sausage, cured pork, lean cured pork); high-caloric sugary beverages (sweetened soft drink, sweetened fruit drinks); low-caloric sugary beverages (unsweetened soft drink, artificially sweetened soft drinks, artificially sweetened fruit drinks); cream soup(cream soup); red meat(beef, lean beef, lamb, veal, lean lamb, pork, lean pork); total butter ( regular and reduced butter from animals); total margarine(regular and reduced fat margarine); non-dark fish(Lean fish, fried fish); poultry(regular and lean poultry, fried chicken); French fries(fried potato); tomato; low-fat dairy (Low-fat cream, low-fat yogurt, sweetened low fat yogurt, artificially sweetened low-fat yogurt, fat-free yogurt, artificially sweetened fat-free yogurt, low-fat cheese, low fat milk, ready to drink flavored reduced fat milk, artificially sweetened non-fat milk, sweetened and artificially sweetened dry milk); eggs; wine; coffee(sweetened, unsweetened, and artificially sweetened coffee); whole fruit(citrus fruit, fruit excluding citrus fruit, fried fruit, avocado and similar); full-fat dairy (full-fat cream, full-fat cheese, full-fat yogurt, sweetened and artificially sweetened full-fat milk, flavored full-fat milk, dairy dessert, frozen dessert, pudding and others ); green-leafy vegetables(dark-green vegetables, vegetables juice).   The rEDIP component foods (serving/d) in MrOS were: Processed meat (cold cuts and sausage, lean cold cuts and sausage, cured pork, lean cured pork); red meat(beef, lean beef, lamb, veal, lean lamb, pork, lean pork); organ meats, non-dark fish(Lean fish-fresh and smoked, fried fish -commercial entree and fast food), other vegetables(other starchy vegetables, other vegetables’, fried vegetables); refined grains(refined grains, flour, and dry mixes, loaf-type bread and plain rolls, quick breads, corn muffins, tortillas, pasta, presweetened and not presweetened ready-to-eat cereal, cakes, cookies, pies, pastries, Danish, doughnuts, cobblers, snack bars, baby food grain mixtures, snacks chips, crackers); high-caloric sugary beverages (sweetened soft drink, sweetened fruit drinks); low-caloric sugary beverages (unsweetened soft drink, artificially sweetened soft drinks, artificially sweetened fruit drinks), tomato, beer; wine; tea(sweetened tea, artificially sweetened tea, unsweetened tea); coffee(sweetened coffee, artificially sweetened coffee, unsweetened coffee); deep-yellow vegetables; green leafy vegetables (dark-green vegetables, vegetable soup); snacks(vegetable-based savory snacks, fruit-based savory snack, whole-grain snack chip, some whole-grain snack chips, refined-grain snack chips, popcorn, flavored popcorn, whole-grain crackers, some whole-grain cracker, refined grain cracker); fruit juice ( citrus juice, fruit juice excluding citrus juice); pizza  The HEI-2020 component foods (servings/d) in the MrOS were: total fruits, whole fruits, total vegetables, greens and beans, whole grains, dairy, total protein foods, sea food and plant proteins, fatty acids, refined grains, sodium, added sugars, saturated fats.  3. Median score for HEI is raw median scores not adjusted for total energy. 4. Calculation of pack-years was restricted among smokers. Values are presented as mean ± SD for continuous variables and percentage for categorical variables. |
| --- |

| **Supplementary Table 2. Multivariable-adjusted absolute values (95% CI) of α-diversity in quintiles of the dietary indices: A cross-sectional analysis at visit 4** | | | | | | | | | | |
| --- | --- | --- | --- | --- | --- | --- | --- | --- | --- | --- |
|  |  | Quintile 1 | Quintile 2 | Quintile 3 | Quintile 4 | Quintile 5 | Percentage difference:  Q5-Q1 | Percentage Difference per SD | p-value | Predicted value (min,max) |
| **Reversed Empirical Dietary Index for Hyperinsulinemia (rEDIH)** | | | | | | | | | | |
| Shannon diversity (n=795) | ^a^MV | 3.437 (3.324, 3.550) | 3.495 (3.384, 3.607) | 3.446 (3.337, 3.554) | 3.556 (3.441, 3.671) | 3.529 (3.417, 3.641) | 2.674 (-2.256, 7.289) | 1.006 (-0.176, 2.188) | 0.0956 | 3.14, 3.97 |
|  | ^b^MV+BMI | 3.441 (3.328, 3.554) | 3.494 (3.382, 3.606) | 3.442 (3.333, 3.550) | 3.553 (3.438, 3.668) | 3.521 (3.409, 3.634) | 2.320 (-2.638, 6.963) | 0.899 (-0.289, 2.087) | 0.1383 | 3.07, 3.97 |
| Inverse Simpson (n=795) | ^a^MV | 16.967 (14.588, 19.347) | 18.825 (16.476, 21.175) | 18.412 (16.140, 20.683) | 19.404 (16.987, 21.822) | 19.887 (17.528, 22.246) | 17.208 (-4.024, 33.217) | 4.987 (0.625, 9.350) | 0.0253 | 12.68, 27.39 |
|  | ^b^MV+BMI | 17.097 (14.723, 19.471) | 18.788 (16.446, 21.130) | 18.288 (16.022, 20.554) | 19.314 (16.904, 21.724) | 19.640 (17.281, 22.000) | 14.875 (-6.597, 31.112) | 4.386 (0.011, 8.761) | 0.0498 | 11.29, 27.63 |
| Pielou’s Evenness (n=795) | ^a^MV | 0.717 (0.702, 0.732) | 0.722 (0.707, 0.737) | 0.710 (0.696, 0.725) | 0.727 (0.711, 0.742) | 0.733 (0.718, 0.748) | 2.223 (-0.931, 5.246) | 0.804 (0.017, 1.590) | 0.0455 | 0.67, 0.79 |
|  | ^b^MV+BMI | 0.717 (0.702, 0.733) | 0.722 (0.707, 0.737) | 0.710 (0.695, 0.724) | 0.727 (0.711, 0.742) | 0.733 (0.718, 0.748) | 2.144 (-1.030, 5.186) | 0.782 (-0.010, 1.574) | 0.0532 | 0.67, 0.79 |
| **Reversed Empirical Dietary Inflammatory Pattern (rEDIP)** | | | | | | | | | | |
|  |  |  |  |  |  |  |  |  |  |  |
| Shannon diversity (n=795) | ^a^MV | 3.393 (3.286, 3.501) | 3.401 (3.293, 3.509) | 3.574 (3.464, 3.684) | 3.547 (3.436, 3.658) | 3.578 (3.464, 3.693) | 5.459 (0.927, 9.713) | 1.432 (0.350, 2.514) | 0.0096 | 3.08, 3.96 |
|  | ^b^MV+BMI | 3.394 (3.286, 3.501) | 3.402 (3.294, 3.510) | 3.569 (3.459, 3.679) | 3.544 (3.433, 3.655) | 3.572 (3.458, 3.687) | 5.269 (0.721, 9.537) | 1.348 (0.262, 2.434) | 0.0152 | 3.00, 3.96 |
| Inverse Simpson (n=795) | ^a^MV | 16.854 (14.593, 19.116) | 17.006 (14.736, 19.277) | 20.990 (18.683, 23.297) | 18.905 (16.567, 21.244) | 20.434 (18.035, 22.832) | 21.238 (2.229, 35.749) | 4.931 (0.930, 8.931) | 0.0159 | 12.31, 27.74 |
|  | ^b^MV+BMI | 16.869 (14.614, 19.124) | 17.052 (14.787, 19.317) | 20.831 (18.527, 23.136) | 18.807 (16.474, 21.140) | 20.224 (17.826, 22.622) | 19.888 (0.692, 34.556) | 4.434 (0.427, 8.441) | 0.0304 | 9.98, 28.20 |
| Pielou’s Evenness (n=795) | ^a^MV | 0.711 (0.697, 0.726) | 0.712 (0.697, 0.726) | 0.729 (0.714, 0.743) | 0.729 (0.714, 0.744) | 0.732 (0.717, 0.748) | 2.987 (0.039, 5.817) | 0.770 (0.049, 1.491) | 0.0368 | 0.67, 0.79 |
|  | ^b^MV+BMI | 0.711 (0.697, 0.726) | 0.712 (0.697, 0.726) | 0.728 (0.714, 0.743) | 0.729 (0.714, 0.744) | 0.732 (0.717, 0.748) | 2.953 (-0.005, 5.793) | 0.751 (0.026, 1.477) | 0.0426 | 0.67, 0.79 |
| **Healthy Eating Index (HEI-2020)** | | | | | | | | | | |
| Shannon diversity (n=795) | ^a^MV | 3.404 (3.298, 3.510) | 3.407 (3.299, 3.516) | 3.525 (3.415, 3.635) | 3.550 (3.438, 3.663) | 3.608 (3.495, 3.720) | 5.994 (1.495, 10.220) | 2.176 (1.089, 3.262) | 0.0001 | 3.08, 3.99 |
|  | ^b^MV+BMI | 3.405 (3.299, 3.511) | 3.410 (3.301, 3.519) | 3.521 (3.411, 3.632) | 3.548 (3.435, 3.661) | 3.600 (3.487, 3.713) | 5.727 (1.190, 9.989) | 2.080 (0.985, 3.175) | 0.0002 | 3.05, 4.00 |
| Inverse Simpson (n=795) | ^a^MV | 16.943 (14.707, 19.179) | 16.973 (14.688, 19.258) | 19.593 (17.276, 21.910) | 19.979 (17.608, 22.351) | 20.861 (18.496, 23.226) | 23.123 (4.514, 37.394) | 7.799 (3.782, 11.817) | 0.0002 | 11.46, 28.00 |
|  | ^b^MV+BMI | 16.978 (14.747, 19.209) | 17.071 (14.789, 19.353) | 19.460 (17.146, 21.774) | 19.884 (17.517, 22.252) | 20.558 (18.183, 22.933) | 21.087 (2.112, 35.654) | 7.196 (3.153, 11.238) | 0.0005 | 10.80, 29.24 |
| Pielou’s Evenness (n=795) | ^a^MV | 0.710 (0.696, 0.724) | 0.715 (0.700, 0.730) | 0.726 (0.711, 0.741) | 0.726 (0.710, 0.741) | 0.736 (0.721, 0.751) | 3.641 (0.704, 6.460) | 1.234 (0.509, 1.959) | 0.0009 | 0.66, 0.79 |
|  | ^b^MV+BMI | 0.710 (0.696, 0.724) | 0.715 (0.700, 0.730) | 0.726 (0.711, 0.741) | 0.726 (0.710, 0.741) | 0.736 (0.720, 0.751) | 3.608 (0.649, 6.450) | 1.220 (0.488, 1.952) | 0.0011 | 0.66, 0.79 |
|  |  |  |  |  |  |  |  |  |  |  |
| **Multivariable-adjusted absolute values (95% CI) of α-diversity in quintiles of the dietary indices: A longitudinal association at visit 1 and visit 4** | | | | | | | | | | |
| **Reversed Empirical Dietary Index for Hyperinsulinemia (rEDIH)** | | | | | | | | | | |
| Shannon diversity (n=868) | ^a^MV | 3.491 (3.316, 3.666) | 3.497 (3.317, 3.676) | 3.525 (3.349, 3.701) | 3.555 (3.381, 3.730) | 3.566 (3.384, 3.748) | 2.135 (-2.318, 6.163) | 1.113 (0.048, 2.177) | 0.0408 | 3.11, 40.9 |
|  | ^b^MV+BMI | 3.501 (3.326, 3.677) | 3.495 (3.316, 3.674) | 3.521 (3.346, 3.697) | 3.552 (3.378, 3.727) | 3.555 (3.373, 3.738) | 1.548 (-3.077, 5.730) | 0.967 (-0.131, 2.065) | 0.0847 | 3.14, 4.11 |
| Inverse Simpson (n=868) | ^a^MV | 18.084 (14.529, 21.640) | 18.572 (14.926, 22.218) | 19.273 (15.700, 22.846) | 19.132 (15.583, 22.681) | 19.917 (16.219, 23.616) | 10.136 (-8.567, 22.694) | 4.092 (0.262, 7.922) | 0.0366 | 10.82, 29.63 |
|  | ^b^MV+BMI | 18.351 (14.785, 21.918) | 18.523 (14.880, 22.166) | 19.182 (15.611, 22.754) | 19.054 (15.507, 22.601) | 19.650 (15.941, 23.359) | 7.075 (-12.722, 20.430) | 3.357 (-0.590, 7.305) | 0.0959 | 10.72, 30.33 |
| Pielou’s Evenness (n=868) | ^a^MV | 0.719 (0.695, 0.742) | 0.722 (0.698, 0.746) | 0.723 (0.699, 0.746) | 0.727 (0.704, 0.751) | 0.727 (0.703, 0.752) | 1.213 (-1.671, 3.913) | 0.741 (0.038, 1.444) | 0.0391 | 0.66, 0.81 |
|  | ^b^MV+BMI | 0.720 (0.696, 0.743) | 0.721 (0.697, 0.746) | 0.722 (0.699, 0.746) | 0.727 (0.703, 0.750) | 0.727 (0.702, 0.751) | 0.973 (-2.015, 3.772) | 0.691 (-0.034, 1.416) | 0.0622 | 0.66, 0.81 |
| **Reversed Empirical Dietary Inflammatory Pattern (rEDIP)** | | | | | | | | | | |
| Shannon diversity (n=868) | ^a^MV | 3.456 (3.286, 3.625) | 3.503 (3.324, 3.681) | 3.581 (3.403, 3.760) | 3.522 (3.342, 3.702) | 3.647 (3.467, 3.827) | 5.545 (1.052, 9.618) | 1.571 (0.476, 2.666) | 0.005 | 3.08, 4.03 |
|  | ^b^MV+BMI | 3.459 (3.290, 3.629) | 3.501 (3.323, 3.680) | 3.581 (3.402, 3.760) | 3.518 (3.338, 3.698) | 3.643 (3.463, 3.823) | 5.309 (0.788, 9.406) | 1.486 (0.383, 2.590) | 0.0084 | 3.10, 4.07 |
| Inverse Simpson (n=868) | ^a^MV | 17.700 (14.239, 21.161) | 18.694 (15.056, 22.333) | 19.890 (16.246, 23.533) | 18.998 (15.333, 22.663) | 20.719 (17.055, 24.383) | 17.058 (-1.281, 29.398) | 4.700 (0.755, 8.645) | 0.0198 | 11.36, 28.27 |
|  | ^b^MV+BMI | 17.803 (14.344, 21.261) | 18.656 (15.022, 22.290) | 19.880 (16.241, 23.519) | 18.891 (15.229, 22.554) | 20.601 (16.939, 24.263) | 15.719 (-2.884, 28.270) | 4.271 (0.299, 8.243) | 0.0354 | 1160, 29.35 |
| Pielou’s Evenness (n=868) | ^a^MV | 0.717 (0.694, 0.740) | 0.719 (0.695, 0.743) | 0.729 (0.705, 0.753) | 0.720 (0.696, 0.745) | 0.737 (0.713, 0.761) | 2.770 (-0.181, 5.539) | 0.906 (0.183, 1.630) | 0.0143 | 0.66, 0.80 |
|  | ^b^MV+BMI | 0.717 (0.695, 0.740) | 0.719 (0.695, 0.743) | 0.729 (0.705, 0.753) | 0.720 (0.696, 0.744) | 0.737 (0.712, 0.761) | 2.667 (-0.301, 5.450) | 0.872 (0.142, 1.601) | 0.0194 | 0.66, 0.80 |
| **Healthy Eating Index (HEI-2020)** | | | | | | | | | | |
| Shannon diversity (n=868) | ^a^MV | 3.489 (3.318, 3.661) | 3.479 (3.304, 3.654) | 3.533 (3.355, 3.711) | 3.599 (3.420, 3.779) | 3.620 (3.439, 3.802) | 3.751 (-0.696, 7.782) | 1.668 (0.583, 2.753) | 0.0027 | 3.10, 3.99 |
|  | ^b^MV+BMI | 3.489 (3.318, 3.661) | 3.484 (3.309, 3.659) | 3.532 (3.354, 3.710) | 3.599 (3.420, 3.779) | 3.611 (3.428, 3.793) | 3.483 (-1.021, 7.564) | 1.570 (0.470, 2.670) | 0.0053 | 3.12, 4.01 |
| Inverse Simpson (n=868) | ^a^MV | 18.276 (14.798, 21.754) | 17.514 (13.963, 21.066) | 19.103 (15.495, 22.711) | 20.775 (17.132, 24.418) | 21.073 (17.388, 24.759) | 15.306 (-2.205, 27.218) | 6.334 (2.432, 10.236) | 0.0015 | 10.26, 28.26 |
|  | ^b^MV+BMI | 18.272 (14.796, 21.748) | 17.637 (14.084, 21.191) | 19.080 (15.474, 22.686) | 20.778 (17.137, 24.419) | 20.820 (17.121, 24.520) | 13.946 (-4.070, 26.203) | 5.845 (1.890, 9.800) | 0.0039 | 10.74, 28.80 |
| Pielou’s Evenness (n=868) | ^a^MV | 0.718 (0.695, 0.741) | 0.718 (0.694, 0.741) | 0.727 (0.704, 0.751) | 0.730 (0.706, 0.754) | 0.735 (0.710, 0.759) | 2.303 (-0.597, 5.024) | 0.929 (0.212, 1.647) | 0.0113 | 0.66, 0.80 |
|  | ^b^MV+BMI | 0.718 (0.695, 0.741) | 0.718 (0.695, 0.742) | 0.727 (0.704, 0.751) | 0.730 (0.706, 0.754) | 0.734 (0.709, 0.758) | 2.201 (-0.732, 4.951) | 0.890 (0.162, 1.618) | 0.0168 | 0.66, 0.80 |
|  |  |  |  |  |  |  |  |  |  |  |
| **Multivariable-adjusted absolute values (95% CI) of α-diversity in quintiles of the dietary indices: Associations with dietary score changes at visits 1 and 4** | | | | | | | | | | |
| **Reversed Empirical Dietary Index for Hyperinsulinemia (rEDIH)** | | | | | | | | | | |
| Shannon diversity (n=758) | ^a^MV | 3.514 (3.328, 3.700) | 3.550 (3.357, 3.742) | 3.544 (3.351, 3.738) | 3.567 (3.375, 3.759) | 3.540 (3.355, 3.725) | 0.743 (-4.266, 5.248) | 0.146 (-0.824, 1.117) | 0.7675 | 3.11, 4.11 |
|  | ^b^MV+BMI | 3.506 (3.320, 3.692) | 3.544 (3.352, 3.736) | 3.540 (3.347, 3.734) | 3.566 (3.375, 3.758) | 3.551 (3.365, 3.736) | 1.274 (-3.776, 5.815) | 0.302 (-0.684, 1.288) | 0.5481 | 3.08, 4.17 |
| Inverse Simpson (n=758) | ^a^MV | 18.164 (14.243, 22.086) | 19.617 (15.562, 23.671) | 19.207 (15.126, 23.289) | 18.587 (14.538, 22.637) | 19.575 (15.669, 23.480) | 7.765 (-14.993, 22.442) | 1.064 (-2.527, 4.655) | 0.5616 | 11.46, 30.06 |
|  | ^b^MV+BMI | 17.934 (14.019, 21.850) | 19.452 (15.406, 23.497) | 19.098 (15.028, 23.169) | 18.578 (14.541, 22.616) | 19.877 (15.974, 23.780) | 10.831 (-11.689, 25.281) | 1.836 (-1.804, 5.477) | 0.3232 | 10.51, 31.90 |
| Pielou’s Evenness (n=758) | ^a^MV | 0.721 (0.696, 0.746) | 0.724 (0.698, 0.750) | 0.721 (0.695, 0.747) | 0.728 (0.702, 0.754) | 0.726 (0.701, 0.751) | 0.658 (-2.567, 3.666) | 0.126 (-0.519, 0.771) | 0.7020 | 0.65, 0.81 |
|  | ^b^MV+BMI | 0.720 (0.695, 0.745) | 0.723 (0.697, 0.749) | 0.721 (0.695, 0.747) | 0.728 (0.702, 0.754) | 0.727 (0.702, 0.752) | 0.855 (-2.407, 3.899) | 0.182 (-0.473, 0.838) | 0.5863 | 0.65, 0.81 |
| **Reversed Empirical Dietary Inflammatory Pattern (rEDIP)** | | | | | | | | | | |
| Shannon diversity (n=758) | ^a^MV | 3.546 (3.360, 3.733) | 3.469 (3.279, 3.659) | 3.594 (3.406, 3.781) | 3.559 (3.370, 3.749) | 3.499 (3.311, 3.687) | -1.329 (-6.342, 3.184) | -0.044 (-1.059, 0.970) | 0.9315 | 3.11, 4.11 |
|  | ^b^MV+BMI | 3.545 (3.358, 3.731) | 3.467 (3.278, 3.657) | 3.590 (3.403, 3.777) | 3.562 (3.372, 3.751) | 3.501 (3.314, 3.689) | -1.224 (-6.231, 3.283) | -0.009 (-1.022, 1.005) | 0.9867 | 3.09, 4.16 |
| Inverse Simpson (n=758) | ^a^MV | 18.736 (14.791, 22.680) | 18.244 (14.227, 22.262) | 19.553 (15.590, 23.516) | 19.848 (15.834, 23.863) | 18.383 (14.415, 22.351) | -1.884 (-26.114, 13.919) | 0.298 (-3.453, 4.050) | 0.8762 | 11.64, 29.88 |
|  | ^b^MV+BMI | 18.697 (14.762, 22.632) | 18.211 (14.203, 22.219) | 19.443 (15.489, 23.398) | 19.914 (15.909, 23.919) | 18.452 (14.493, 22.410) | -1.313 (-25.394, 14.395) | 0.474 (-3.271, 4.220) | 0.8041 | 10.88, 31.47 |
| Pielou’s Evenness (n=758) | ^a^MV | 0.728 (0.703, 0.753) | 0.713 (0.687, 0.738) | 0.730 (0.705, 0.755) | 0.728 (0.703, 0.754) | 0.717 (0.692, 0.742) | -1.496 (-4.724, 1.516) | -0.187 (-0.861, 0.486) | 0.5858 | 0.65, 0.81 |
|  | ^b^MV+BMI | 0.728 (0.702, 0.753) | 0.713 (0.687, 0.738) | 0.730 (0.705, 0.755) | 0.728 (0.703, 0.754) | 0.717 (0.692, 0.742) | -1.462 (-4.691, 1.552) | -0.175 (-0.849, 0.499) | 0.6105 | 0.65, 0.81 |
| **Healthy Eating Index (HEI-2020)** | | | | | | | | | | |
| Shannon diversity (n=758) | ^a^MV | 3.483 (3.291, 3.676) | 3.494 (3.306, 3.682) | 3.554 (3.371, 3.737) | 3.573 (3.385, 3.761) | 3.545 (3.356, 3.735) | 1.779 (-2.917, 5.984) | 0.910 (-0.207, 2.027) | 0.1108 | 3.07, 4.10 |
|  | ^b^MV+BMI | 3.487 (3.295, 3.679) | 3.489 (3.302, 3.677) | 3.552 (3.369, 3.735) | 3.574 (3.386, 3.761) | 3.548 (3.359, 3.738) | 1.755 (-2.931, 5.952) | 0.917 (-0.199, 2.034) | 0.1076 | 3.04, 4.15 |
| Inverse Simpson (n=758) | ^a^MV | 18.302 (14.239, 22.365) | 17.942 (13.974, 21.910) | 19.450 (15.580, 23.320) | 19.435 (15.463, 23.407) | 18.985 (14.980, 22.990) | 3.730 (-18.641, 17.973) | 2.221 (-1.916, 6.359) | 0.2930 | 11.06, 29.78 |
|  | ^b^MV+BMI | 18.409 (14.355, 22.462) | 17.822 (13.863, 21.781) | 19.398 (15.537, 23.258) | 19.454 (15.492, 23.416) | 19.070 (15.074, 23.065) | 3.590 (-18.584, 17.760) | 2.258 (-1.870, 6.385) | 0.2841 | 10.27, 31.41 |
| Pielou’s Evenness (n=758) | ^a^MV | 0.720 (0.694, 0.746) | 0.713 (0.688, 0.738) | 0.727 (0.703, 0.752) | 0.729 (0.704, 0.755) | 0.726 (0.700, 0.751) | 0.736 (-2.293, 3.555) | 0.561 (-0.181, 1.304) | 0.1386 | 0.65, 0.81 |
|  | ^b^MV+BMI | 0.721 (0.695, 0.746) | 0.713 (0.687, 0.738) | 0.727 (0.703, 0.752) | 0.730 (0.704, 0.755) | 0.726 (0.700, 0.751) | 0.728 (-2.301, 3.546) | 0.564 (-0.178, 1.306) | 0.1369 | 0.65, 0.81 |
| Values are measure of alpha-diversity, p-value of < 0.05 represent statistically significant findings. Beta coefficients were derived from ^a^MV (multivariable) adjusted, and ^b^multivariable + BMI (body mass index) adjusted linear regression models. Multivariable linear regression models were adjusted for total energy intake, race/ethnicity (White, others), marital status (married, others), education (less than high school, high school and higher), physical activity (continuous), smoking status (never, past/ current), pack-years of smoking (continuous), number of nutritional supplements used (continuous), total alcohol intake (continuous, servings/day), aspirin/other NSAID use (yes vs no), baseline comorbidity score conditions (continuous). | | | | | | | | | | |

| **Supplementary Table 3. Microbial genera selected by elastic net regression in cross-sectional study across three dietary patterns and their Beta coefficients** | | | | | |
| --- | --- | --- | --- | --- | --- |
| **Genus_rEDIH** | **Beta** | **Genus_rEDIP** | **Beta** | **Genus_HEI-2020** | **Beta** |
| g__Anaerostipes | 0.0086 | g__Acidaminococcus | -0.0101 | g__Acidaminococcus | -0.0079 |
| g__Bilophila | -0.0102 | g__Alistipes | -0.0032 | g__Alistipes | -0.0038 |
| g__Blautia | 0.0042 | g__Anaerofilum | 0.0002 | g__Anaerofilum | -0.0006 |
| g__CAG-56 | -0.0083 | g__Anaerostipes | 0.0102 | g__Anaerotruncus | -0.0194 |
| g__Candidatus_Stoquefichus | 0.0186 | g__Angelakisella | -0.0008 | g__Barnesiella | 0.0015 |
| g__Caproiciproducens | -0.0005 | g__Bacteroides | -0.0037 | g__Butyricicoccus | 0.0010 |
| g__Coprococcus_2 | -0.0002 | g__Bifidobacterium | -0.0004 | g__Butyrivibrio | 0.0000 |
| g__Dielma | -0.0164 | g__Bilophila | 0.0000 | g__Caproiciproducens | -0.0008 |
| g__Dorea | -0.0107 | g__Blautia | 0.0023 | g__Catenibacterium | 0.0055 |
| g__Escherichia/Shigella | -0.0035 | g__Butyricicoccus | 0.0001 | g__Christensenellaceae_R-7_group | 0.0084 |
| g__Faecalitalea | -0.0312 | g__CAG-352 | 0.0001 | g__Clostridium_sensu_stricto_1 | 0.0004 |
| g__Family_XIII_AD3011_group | -0.0053 | g__Candidatus_Stoquefichus | 0.0042 | g__Coprobacter | 0.0007 |
| g__Fournierella | 0.0002 | g__Caproiciproducens | -0.0004 | g__Coprococcus_1 | 0.0005 |
| g__GCA-900066575 | 0.0061 | g__Christensenellaceae_R-7_group | 0.0006 | g__Coprococcus_2 | 0.0008 |
| g__Haemophilus | 0.0004 | g__Clostridium_sensu_stricto_1 | 0.0014 | g__Desulfovibrio | -0.0133 |
| g__Holdemanella | -0.0019 | g__Coprococcus_2 | 0.0003 | g__Dielma | -0.0166 |
| g__Holdemania | 0.0002 | g__Dielma | -0.0423 | g__Dorea | 0.0009 |
| g__Howardella | -0.0182 | g__Dorea | 0.0002 | g__Eisenbergiella | -0.0169 |
| g__Intestinibacter | 0.0309 | g__DTU089 | -0.0001 | g__Erysipelatoclostridium | -0.0008 |
| g__Lachnoclostridium | 0.0004 | g__Erysipelatoclostridium | 0.0025 | g__Erysipelotrichaceae_UCG-003 | 0.0052 |
| g__Lachnospira | 0.0036 | g__Erysipelotrichaceae_UCG-003 | 0.0182 | g__Escherichia/Shigella | -0.0042 |
| g__Lachnospiraceae_NC2004_group | 0.0058 | g__Escherichia/Shigella | -0.0054 | g__Faecalibacterium | 0.0021 |
| g__Lachnospiraceae_UCG-003 | 0.0088 | g__Faecalitalea | -0.0210 | g__Faecalitalea | -0.0101 |
| g__Lactobacillus | -0.0010 | g__Family_XIII_UCG-001 | 0.0061 | g__Flavonifractor | -0.0011 |
| g__Marvinbryantia | 0.0045 | g__Flavonifractor | -0.0001 | g__Fournierella | 0.0134 |
| g__Methanobrevibacter | -0.0061 | g__Fournierella | 0.0001 | g__Fusicatenibacter | 0.0073 |
| g__Mogibacterium | -0.0001 | g__Fusobacterium | -0.0005 | g__Fusobacterium | -0.0047 |
| g__Negativibacillus | -0.0117 | g__GCA-900066575 | 0.0196 | g__Haemophilus | 0.0084 |
| g__Oxalobacter | -0.0006 | g__Haemophilus | 0.0001 | g__Hungatella | -0.0057 |
| g__Parasutterella | -0.0013 | g__Holdemania | -0.0006 | g__Intestinibacter | 0.0124 |
| g__Peptococcus | -0.0261 | g__Intestinibacter | 0.0255 | g__Intestinimonas | -0.0004 |
| g__Phascolarctobacterium | 0.0039 | g__Klebsiella | -0.0038 | g__Lachnospira | 0.0104 |
| g__Prevotella_9 | -0.0002 | g__Lachnoclostridium | -0.0159 | g__Lachnospiraceae_AC2044_group | 0.0035 |
| g__Pseudomonas | 0.0037 | g__Lachnospira | 0.0008 | g__Lachnospiraceae_FCS020_group | 0.0252 |
| g__Roseburia | 0.0154 | g__Lachnospiraceae_UCG-001 | 0.0011 | g__Lachnospiraceae_ND3007_group | 0.0038 |
| g__Ruminiclostridium_1 | 0.0004 | g__Lachnospiraceae_UCG-003 | 0.0142 | g__Lachnospiraceae_UCG-001 | 0.0171 |
| g__Ruminiclostridium_5 | -0.0007 | g__Lachnospiraceae_UCG-004 | 0.0004 | g__Lachnospiraceae_UCG-003 | 0.0128 |
| g__Ruminiclostridium_9 | 0.0009 | g__Lachnospiraceae_UCG-010 | -0.0006 | g__Lachnospiraceae_UCG-004 | 0.0018 |
| g__Ruminococcaceae_UCG-009 | -0.0036 | g__Marvinbryantia | 0.0076 | g__Lactobacillus | -0.0011 |
| g__Ruminococcus_1 | 0.0061 | g__Methanomassiliicoccus | 0.0000 | g__Methanomassiliicoccus | -0.0011 |
| g__Sellimonas | 0.0081 | g__Negativibacillus | -0.0214 | g__Mogibacterium | -0.0009 |
| g__Subdoligranulum | 0.0000 | g__Odoribacter | -0.0004 | g__Negativibacillus | -0.0067 |
| g__Terrisporobacter | 0.0010 | g__Oscillibacter | -0.0005 | g__Odoribacter | 0.0190 |
| g__Turicibacter | 0.0007 | g__Parabacteroides | -0.0001 | g__Paraprevotella | 0.0180 |
| g__Tyzzerella_4 | -0.0244 | g__Paraprevotella | 0.0001 | g__Peptococcus | -0.0336 |
| g__UC5-1-2E3 | -0.0088 | g__Parasutterella | -0.0034 | g__Romboutsia | 0.0003 |
|  |  | g__Peptococcus | -0.0155 | g__Ruminiclostridium_5 | -0.0060 |
|  |  | g__Prevotella_9 | 0.0150 | g__Ruminiclostridium_9 | -0.0009 |
|  |  | g__Romboutsia | 0.0005 | g__Ruminococcaceae_NK4A214_group | 0.0039 |
|  |  | g__Roseburia | 0.0037 | g__Ruminococcaceae_UCG-002 | 0.0106 |
|  |  | g__Ruminiclostridium_9 | -0.0008 | g__Ruminococcaceae_UCG-003 | 0.0006 |
|  |  | g__Ruminococcaceae_UCG-003 | 0.0005 | g__Ruminococcaceae_UCG-005 | 0.0006 |
|  |  | g__Ruminococcaceae_UCG-008 | 0.0020 | g__Ruminococcaceae_UCG-008 | 0.0160 |
|  |  | g__Ruminococcaceae_UCG-009 | 0.0000 | g__Ruminococcaceae_UCG-009 | -0.0148 |
|  |  | g__Ruminococcaceae_UCG-014 | 0.0002 | g__Ruminococcaceae_UCG-010 | 0.0016 |
|  |  | g__Shuttleworthia | 0.0190 | g__Ruminococcaceae_UCG-013 | 0.0003 |
|  |  | g__Subdoligranulum | 0.0033 | g__Ruminococcaceae_UCG-014 | 0.0066 |
|  |  | g__Sutterella | 0.0003 | g__Ruminococcus_1 | 0.0002 |
|  |  | g__Terrisporobacter | 0.0005 | g__Ruminococcus_2 | -0.0007 |
|  |  | g__Turicibacter | 0.0005 | g__Sellimonas | -0.0189 |
|  |  | g__Tyzzerella | 0.0000 | g__Streptococcus | 0.0002 |
|  |  | g__Tyzzerella_4 | -0.0140 | g__Subdoligranulum | 0.0018 |
|  |  | g__UBA1819 | -0.0001 | g__Tyzzerella | -0.0070 |
|  |  | g__UC5-1-2E3 | -0.0088 | g__Tyzzerella_3 | 0.0027 |
|  |  | g__Victivallis | -0.0001 | g__Tyzzerella_4 | -0.0002 |
|  |  |  |  | g__UBA1819 | -0.0005 |
|  |  |  |  | g__UC5-1-2E3 | -0.0001 |
|  |  |  |  | g__Veillonella | 0.0234 |

| **Supplementary Table 4. Microbial genera selected by elastic net regression in the longitudinal study across the three dietary patterns and their Beta coefficients** | | | |
| --- | --- | --- | --- |
| **Genus_rEDIH** | **Beta** | **Genus_HEI-2020** | **Beta** |
| g__Anaerostipes | 0.0037 | g__Acidaminococcus | -0.0055 |
| g__Bilophila | -0.0017 | g__Agathobacter | 0.0004 |
| g__Butyricicoccus | 0.0080 | g__Akkermansia | 0.0043 |
| g__Butyricimonas | 0.0008 | g__Anaerofilum | -0.0008 |
| g__CAG-352 | -0.0032 | g__Anaerotruncus | -0.0154 |
| g__Escherichia/Shigella | -0.0053 | g__Angelakisella | -0.0008 |
| g__Faecalitalea | -0.0198 | g__Bifidobacterium | -0.0025 |
| g__Lachnospira | 0.0004 | g__Butyricicoccus | 0.0247 |
| g__Lachnospiraceae_ND3007_group | 0.0001 | g__Butyricimonas | 0.0000 |
| g__Lachnospiraceae_UCG-001 | 0.0001 | g__CAG-56 | 0.0016 |
| g__Lachnospiraceae_UCG-004 | 0.0001 | g__Christensenellaceae_R-7_group | 0.0080 |
| g__Oscillospira | 0.0003 | g__Clostridium_sensu_stricto_1 | 0.0046 |
| g__Prevotella_7 | 0.0000 | g__Collinsella | 0.0000 |
| g__Pseudomonas | -0.0036 | g__Coprobacter | 0.0038 |
| g__Roseburia | 0.0001 | g__Coprococcus_2 | 0.0057 |
| g__Ruminiclostridium_6 | 0.0006 | g__Coprococcus_3 | -0.0169 |
| g__Ruminiclostridium_9 | 0.0036 | g__Defluviitaleaceae_UCG-011 | 0.0115 |
| g__Ruminococcaceae_UCG-008 | 0.0007 | g__Desulfovibrio | -0.0117 |
| g__Ruminococcaceae_UCG-013 | 0.0001 | g__Dialister | 0.0003 |
| g__Shuttleworthia | 0.0037 | g__Erysipelatoclostridium | 0.0092 |
| g__Tyzzerella_4 | -0.0079 | g__Erysipelotrichaceae_UCG-003 | 0.0059 |
|  |  | g__Escherichia/Shigella | -0.0002 |
| **Genus_rEDIP** | **Beta** | g__Faecalibacterium | 0.0008 |
| g__Acidaminococcus | -0.0063 | g__Faecalitalea | -0.0074 |
| g__Barnesiella | 0.0031 | g__Family_XIII_AD3011_group | 0.0004 |
| g__Butyricicoccus | 0.0053 | g__Flavonifractor | 0.0027 |
| g__Christensenellaceae_R-7_group | 0.0016 | g__Fusobacterium | -0.0309 |
| g__Coprococcus_1 | 0.0000 | g__GCA-900066225 | 0.0003 |
| g__Defluviitaleaceae_UCG-011 | 0.0000 | g__Haemophilus | 0.0133 |
| g__Erysipelotrichaceae_UCG-003 | 0.0031 | g__Holdemanella | 0.0001 |
| g__Faecalitalea | -0.0230 | g__Howardella | 0.0380 |
| g__Family_XIII_UCG-001 | 0.0027 | g__Intestinibacter | 0.0007 |
| g__Fusobacterium | -0.0090 | g__Intestinimonas | 0.0001 |
| g__GCA-900066575 | 0.0060 | g__Klebsiella | 0.0015 |
| g__Haemophilus | -0.0018 | g__Lachnospira | 0.0010 |
| g__Hungatella | -0.0048 | g__Lachnospiraceae_AC2044_group | 0.0106 |
| g__Klebsiella | 0.0000 | g__Lachnospiraceae_FCS020_group | 0.0238 |
| g__Lachnospira | 0.0001 | g__Lachnospiraceae_NK4A136_group | 0.0279 |
| g__Lachnospiraceae_AC2044_group | 0.0016 | g__Lachnospiraceae_UCG-001 | 0.0015 |
| g__Lachnospiraceae_FCS020_group | 0.0022 | g__Lachnospiraceae_UCG-003 | 0.0055 |
| g__Lachnospiraceae_UCG-001 | 0.0004 | g__Lachnospiraceae_UCG-004 | 0.0246 |
| g__Lachnospiraceae_UCG-003 | 0.0220 | g__Lactobacillus | -0.0134 |
| g__Oscillospira | 0.0026 | g__Methanomassiliicoccus | -0.0013 |
| g__Parabacteroides | 0.0060 | g__Negativibacillus | -0.0064 |
| g__Paraprevotella | 0.0002 | g__Parabacteroides | -0.0002 |
| g__Roseburia | 0.0028 | g__Paraprevotella | 0.0065 |
| g__Ruminococcaceae_NK4A214_group | 0.0005 | g__Parasutterella | 0.0003 |
| g__Ruminococcaceae_UCG-002 | 0.0006 | g__Peptococcus | -0.0167 |
| g__Ruminococcaceae_UCG-003 | 0.0003 | g__Phascolarctobacterium | -0.0034 |
| g__Ruminococcaceae_UCG-005 | 0.0001 | g__Prevotella_7 | -0.0071 |
| g__Ruminococcaceae_UCG-008 | 0.0147 | g__Prevotella_9 | 0.0042 |
| g__Ruminococcaceae_UCG-009 | 0.0004 | g__Pseudomonas | -0.0158 |
| g__Ruminococcaceae_UCG-010 | 0.0040 | g__Romboutsia | 0.0189 |
| g__Ruminococcaceae_UCG-011 | 0.0004 | g__Roseburia | 0.0024 |
| g__Shuttleworthia | 0.0245 | g__Ruminiclostridium_1 | -0.0165 |
| g__Tyzzerella_4 | -0.0003 | g__Ruminococcaceae_NK4A214_group | 0.0021 |
| g__UBA1819 | -0.0025 | g__Ruminococcaceae_UCG-003 | 0.0000 |
|  |  | g__Ruminococcaceae_UCG-008 | 0.0063 |
|  |  | g__Ruminococcaceae_UCG-009 | 0.0003 |
|  |  | g__Ruminococcaceae_UCG-010 | 0.0004 |
|  |  | g__Ruminococcaceae_UCG-011 | 0.0004 |
|  |  | g__Ruminococcaceae_UCG-013 | 0.0004 |
|  |  | g__Ruminococcaceae_UCG-014 | 0.0015 |
|  |  | g__Ruminococcus_1 | 0.0094 |
|  |  | g__Sellimonas | -0.0050 |
|  |  | g__Streptococcus | -0.0137 |
|  |  | g__Subdoligranulum | 0.0004 |
|  |  | g__Terrisporobacter | 0.0000 |
|  |  | g__Turicibacter | 0.0007 |
|  |  | g__Tyzzerella | -0.0028 |

| **Supplementary Table 5. Microbial genera selected by elastic net regression in change in dietary score study across three dietary patterns and their Beta coefficients** | | | | | |
| --- | --- | --- | --- | --- | --- |
| **Genus_rEDIH** | **Beta** | **Genus_rEDIP** | **Beta** | **Genus_HEI-2020** | **Beta** |
| g__Akkermansia | 0.0045 | g__Acidaminococcus | -0.0024 | g__Agathobacter | 0.0008 |
| g__Bacteroides | -0.0009 | g__Akkermansia | -0.0002 | g__Christensenellaceae_R-7_group | 0.0006 |
| g__CAG-56 | -0.0316 | g__Anaerostipes | 0.0126 | g__Coprococcus_1 | 0.0005 |
| g__Coprococcus_2 | 0.0134 | g__Anaerotruncus | -0.0009 | g__Coprococcus_2 | 0.0001 |
| g__Coprococcus_3 | -0.0082 | g__Bacteroides | -0.0031 | g__Dorea | 0.0005 |
| g__Defluviitaleaceae_UCG-011 | -0.0097 | g__Bifidobacterium | 0.0002 | g__Fusicatenibacter | 0.0000 |
| g__Erysipelotrichaceae_UCG-003 | 0.0019 | g__Bilophila | -0.0024 | g__Lachnospira | 0.0103 |
| g__Escherichia/Shigella | -0.0042 | g__Butyrivibrio | 0.0022 | g__Lachnospiraceae_FCS020_group | 0.0081 |
| g__Family_XIII_AD3011_group | -0.0118 | g__CAG-56 | -0.0131 | g__Lachnospiraceae_ND3007_group | 0.0187 |
| g__Family_XIII_UCG-001 | 0.0029 | g__Candidatus_Stoquefichus | 0.0033 | g__Lachnospiraceae_UCG-001 | 0.0072 |
| g__Fusobacterium | 0.0033 | g__Catenibacterium | 0.0052 | g__Ruminiclostridium_6 | 0.0006 |
| g__GCA-900066575 | 0.0177 | g__Christensenellaceae_R-7_group | 0.0095 | g__Ruminococcaceae_NK4A214_group | 0.0002 |
| g__Holdemania | 0.0059 | g__Clostridium_sensu_stricto_1 | 0.0057 | g__Ruminococcaceae_UCG-002 | 0.0033 |
| g__Hungatella | -0.0002 | g__Coprococcus_1 | 0.0004 | g__Ruminococcaceae_UCG-003 | 0.0016 |
| g__Intestinibacter | 0.0107 | g__Coprococcus_2 | 0.0018 | g__Ruminococcaceae_UCG-005 | 0.0018 |
| g__Lachnoclostridium | 0.0011 | g__Defluviitaleaceae_UCG-011 | -0.0165 | g__Ruminococcaceae_UCG-010 | 0.0013 |
| g__Lachnospira | 0.0109 | g__Dielma | -0.0323 | g__Ruminococcaceae_UCG-013 | 0.0074 |
| g__Lachnospiraceae_FCS020_group | 0.0008 | g__Dorea | 0.0001 | g__Ruminococcaceae_UCG-014 | 0.0051 |
| g__Lachnospiraceae_NC2004_group | 0.0205 | g__Erysipelatoclostridium | 0.0013 | g__Ruminococcus_1 | 0.0003 |
| g__Lachnospiraceae_UCG-003 | 0.0005 | g__Erysipelotrichaceae_UCG-003 | 0.0101 | g__Subdoligranulum | 0.0005 |
| g__Marvinbryantia | 0.0160 | g__Escherichia/Shigella | -0.0094 | g__Tyzzerella | -0.0002 |
| g__Methanomassiliicoccus | 0.0016 | g__Family_XIII_UCG-001 | 0.0103 |  |  |
| g__Negativibacillus | -0.0136 | g__GCA-900066575 | 0.0188 |  |  |
| g__Peptococcus | -0.0091 | g__Holdemanella | 0.0051 |  |  |
| g__Prevotella_7 | 0.0089 | g__Holdemania | -0.0001 |  |  |
| g__Ruminiclostridium | 0.0004 | g__Intestinibacter | 0.0109 |  |  |
| g__Ruminiclostridium_1 | 0.0021 | g__Klebsiella | -0.0024 |  |  |
| g__Ruminococcaceae_UCG-008 | 0.0029 | g__Lachnoclostridium | -0.0031 |  |  |
| g__Ruminococcaceae_UCG-009 | -0.0073 | g__Lachnospira | 0.0091 |  |  |
| g__Ruminococcaceae_UCG-011 | 0.0068 | g__Lachnospiraceae_NC2004_group | 0.0225 |  |  |
| g__Ruminococcaceae_UCG-013 | 0.0042 | g__Lachnospiraceae_ND3007_group | 0.0026 |  |  |
| g__Ruminococcaceae_UCG-014 | 0.0042 | g__Lachnospiraceae_NK4B4_group | 0.0074 |  |  |
| g__Ruminococcus_1 | 0.0054 | g__Negativibacillus | -0.0051 |  |  |
| g__Sellimonas | 0.0482 | g__Odoribacter | 0.0006 |  |  |
| g__Subdoligranulum | -0.0016 | g__Oxalobacter | -0.0039 |  |  |
| g__Tyzzerella | 0.0070 | g__Peptococcus | -0.0206 |  |  |
| g__Tyzzerella_3 | 0.0021 | g__Prevotella_9 | 0.0181 |  |  |
| g__Tyzzerella_4 | -0.0033 | g__Romboutsia | 0.0017 |  |  |
| g__UC5-1-2E3 | -0.0064 | g__Roseburia | -0.0004 |  |  |
| g__Victivallis | 0.0062 | g__Ruminiclostridium | 0.0003 |  |  |
|  |  | g__Ruminococcaceae_UCG-003 | -0.0049 |  |  |
|  |  | g__Ruminococcaceae_UCG-008 | 0.0012 |  |  |
|  |  | g__Ruminococcaceae_UCG-009 | -0.0215 |  |  |
|  |  | g__Ruminococcaceae_UCG-014 | 0.0038 |  |  |
|  |  | g__UBA1819 | 0.0058 |  |  |
|  |  | g__UC5-1-2E3 | -0.0022 |  |  |
|  |  | g__Veillonella | -0.0003 |  |  |
|  |  | g__Victivallis | 0.0014 |  |  |

| **Supplementary Table 6. Pathway analysis - associations of the dietary pattern scores**  **with named microbial pathways derived from PiCRUST** | | | | |
| --- | --- | --- | --- | --- |
| **Cross-sectional analysis** |  |  |  |  |
| **Reversed Empirical Dietary Index for Hyperinsulinemia (rEDIH)** | |  |  |  |
| Pathways | PD per SD | p value | FDR p value |  |
| Superpathway of aromatic amino acid biosynthesis | -0.09 (-0.17, -0.02) | 0.0160 | 0.0981 |  |
| Superpathway of aromatic amino acid biosynthesis | -0.09 (-0.17, -0.02) | 0.0162 | 0.0981 |  |
| superpathway of N-acetylglucosamine | -0.09 (-0.16, -0.02) | 0.0096 | 0.0866 |  |
| glycogen degradation I | -0.09 (-0.16, -0.02) | 0.0152 | 0.0981 |  |
| glycogen biosynthesis I (from ADP-D-Glucose) | -0.09 (-0.17, -0.02) | 0.0126 | 0.0948 |  |
| superpathway of phospholipid biosynthesis III | -0.09 (-0.17, -0.02) | 0.0138 | 0.0977 |  |
| CDP-diacylglycerol biosynthesis II | -0.09 (-0.17, -0.02) | 0.0160 | 0.0981 |  |
| nitrate reduction VI (assimilatory) | -0.08 (-0.13, -0.02) | 0.0092 | 0.0866 |  |
| CDP-diacylglycerol biosynthesis I | -0.09 (-0.17, -0.02) | 0.0160 | 0.0981 |  |
| Escherichia coli K-12 substrate | -0.09 (-0.16, -0.02) | 0.0105 | 0.0894 |  |
| sucrose degradation III (sucrose invertase) | -0.10 (-0.17, -0.02) | 0.0094 | 0.0866 |  |
| D-galactose degradation I (Leloir pathway) | -0.09 (-0.16, -0.02) | 0.0150 | 0.0981 |  |
| GDP-D-glycero-α-D-manno-heptose biosynthesis | -0.09 (-0.16, -0.02) | 0.0128 | 0.0948 |  |
| starch degradation V | -0.10 (-0.17, -0.02) | 0.0099 | 0.0866 |  |
| NAD salvage pathway I (PNC VI cycle) | -0.09 (-0.16, -0.02) | 0.0082 | 0.0797 |  |
|  |  |  |  |  |
| **Reversed Empirical Dietary Inflammatory Index (rEDIP)** |  |  |  |  |
| Pathways | PD per SD | p value | FDR p value |  |
| Escherichia coli K-12 substr | -0.09 (-0.16, -0.03) | 0.0048 | 0.0746 |  |
| dTDP-L-rhamnose biosynthesis | -0.08 (-0.15, -0.02) | 0.0114 | 0.0934 |  |
| D-galacturonate degradation I | -0.08 (-0.14, -0.02) | 0.0116 | 0.0934 |  |
| Superpathway of hexuronide and hexuronate degradation | -0.08 (-0.14, -0.02) | 0.0124 | 0.0948 |  |
| Superpathway of N-acetylglucosamine | -0.12 (-0.19, -0.06) | 0.0001 | 0.0378 |  |
| Glycogen degradation I | -0.10 (-0.16, -0.03) | 0.0038 | 0.0746 |  |
| NAD de novo biosynthesis II (from tryptophan) | -0.08 (-0.14, -0.02) | 0.0063 | 0.0754 |  |
| Acetylene degradation (anaerobic) | -0.10 (-0.17, -0.04) | 0.0019 | 0.0704 |  |
| Purine nucleobases degradation I | -0.09 (-0.15, -0.03) | 0.0043 | 0.0746 |  |
| Escherichia coli K-12 substr. MG1655 pentose phosphate pathway | -0.08 (-0.14, -0.02) | 0.0111 | 0.0924 |  |
| Purine ribonucleosides degradation | -0.09 (-0.16, -0.02) | 0.0072 | 0.0773 |  |
| Halomonas sp. TD01 Halomonas Bluephagenesis substr. TD01 ... | -0.10 (-0.17, -0.04) | 0.0022 | 0.0733 |  |
| Mycobacterium tuberculosis H37Rv superpathway of pyrimidine ... | -0.10 (-0.17, -0.04) | 0.0018 | 0.0704 |  |
| Formaldehyde assimilation II (assimilatory RuMP Cycle) | -0.09 (-0.15, -0.03) | 0.0044 | 0.0746 |  |
| Escherichia coli K-12 substr. MG1655 S-adenosyl-L ... | -0.10 (-0.17, -0.04) | 0.0019 | 0.0704 |  |
| Sucrose degradation III (sucrose invertase) | -0.10 (-0.17, -0.03) | 0.0033 | 0.0746 |  |
| D-galactose degradation I (Leloir pathway) | -0.09 (-0.16, -0.02) | 0.0076 | 0.0773 |  |
| GDP-D-glycero-α-D-manno-heptose biosynthesis | -0.11 (-0.18, -0.05) | 0.0004 | 0.0379 |  |
| Starch degradation V | -0.09 (-0.15, -0.02) | 0.0136 | 0.0974 |  |
| D-fructuronate degradation | -0.08 (-0.14, -0.01) | 0.0151 | 0.0981 |  |
| NAD salvage pathway I (PNC VI cycle) | -0.12 (-0.18, -0.06) | 0.0002 | 0.0378 |  |
| Formaldehyde assimilation II (assimilatory RuMP Cycle) | -0.08 (-0.14, -0.02) | 0.0064 | 0.0754 |  |
|  |  |  |  |  |
| **Healthy Eating Index (HEI-2020)** |  |  |  |  |
| Pathways | PD per SD | p value | FDR p value |  |
| Homolactic fermentation | -0.08 (-0.14, -0.02) | 0.0128 | 0.0948 |  |
| Glycolysis III (from glucose) | -0.10 (-0.17, -0.03) | 0.0031 | 0.0746 |  |
| superpathway of branched chain amino acid biosynthesis | -0.09 (-0.16, -0.03) | 0.0074 | 0.0773 |  |
| Calvin-Benson-Bassham cycle | -0.10 (-0.16, -0.03) | 0.005 | 0.0754 |  |
| Coenzyme A biosynthesis I (bacteria) | -0.08 (-0.15, -0.02) | 0.0167 | 0.0997 |  |
| Escherichia coli K-12 substr. MG1655 superpathway of ... | -0.09 (-0.15, -0.02) | 0.0154 | 0.0981 |  |
| Escherichia coli K-12 substr. MG1655 L-lysine biosynthesis I | -0.10 (-0.16, -0.03) | 0.0034 | 0.0746 |  |
| dTDP-L-rhamnose biosynthesis | -0.10 (-0.16, -0.03) | 0.0045 | 0.0746 |  |
| Superpathway of hexuronide and hexuronate degradation | -0.10 (-0.16, -0.03) | 0.0025 | 0.0746 |  |
| D-galacturonate degradation I | -0.09 (-0.15, -0.03) | 0.0035 | 0.0746 |  |
| Superpathway of N-acetylglucosamine | -0.12 (-0.18, -0.06) | 0.0003 | 0.0378 |  |
| Gluconeogenesis I | -0.08 (-0.14, -0.01) | 0.0157 | 0.0981 |  |
| superpathway of beta-D-glucuronosides degradation | -0.08 (-0.15, -0.02) | 0.0083 | 0.0797 |  |
| Free Form Advanced Query Page | -0.11 (-0.18, -0.05) | 0.0007 | 0.0445 |  |
| Escherichia coli K-12 substr. MG1655 glycolysis I (from glucose 6 ... | -0.08 (-0.14, -0.01) | 0.0167 | 0.0997 |  |
| Escherichia coli K-12 substr. MG1655 L-isoleucine biosynthesis I ... | -0.09 (-0.16, -0.02) | 0.0098 | 0.0866 |  |
| NAD de novo biosynthesis II (from tryptophan) | -0.09 (-0.15, -0.03) | 0.0041 | 0.0746 |  |
| pentose phosphate pathway (non-oxidative branch) I | -0.09 (-0.16, -0.02) | 0.008 | 0.0797 |  |
| acetylene degradation (anaerobic) | -0.10 (-0.17, -0.04) | 0.0022 | 0.0733 |  |
| purine nucleobases degradation I | -0.09 (-0.15, -0.03) | 0.0038 | 0.0746 |  |
| Escherichia coli K-12 substr. MG1655 pentose phosphate pathway | -0.09 (-0.15, -0.02) | 0.0063 | 0.0754 |  |
| formaldehyde assimilation II (assimilatory RuMP Cycle) | -0.10 (-0.17, -0.04) | 0.0027 | 0.0746 |  |
| Arabidopsis thaliana col L-lysine biosynthesis VI - Shigella flexneri | -0.10 (-0.16, -0.03) | 0.0031 | 0.0746 |  |
| pyruvate fermentation to acetate and lactate II | -0.10 (-0.17, -0.03) | 0.0028 | 0.0746 |  |
| L-isoleucine biosynthesis II | -0.08 (-0.13, -0.03) | 0.0027 | 0.0746 |  |
| GDP-d-glycero-α-d-manno-heptose | -0.09 (-0.16, -0.03) | 0.007 | 0.0756 |  |
| L-isoleucine biosynthesis IV | -0.09 (-0.16, -0.03) | 0.007 | 0.0756 |  |
| L-arginine biosynthesis III (via N-acetyl-L-citrulline) | -0.09 (-0.15, -0.03) | 0.0031 | 0.0746 |  |
| [XML] https://biocyc.org/META/pathway-biopax?type=3&object=PWY-5384 | -0.08 (-0.15, -0.02) | 0.016 | 0.0981 |  |
| Escherichia coli K-12 substr. MG1655 S-adenosyl-L ... | -0.08 (-0.15, -0.02) | 0.0162 | 0.0981 |  |
| sucrose degradation III (sucrose invertase) | -0.10 (-0.16, -0.03) | 0.006 | 0.0754 |  |
| D-galactose degradation I (Leloir pathway) | -0.09 (-0.16, -0.03) | 0.0074 | 0.0773 |  |
| GDP-D-glycero-α-D-manno-heptose biosynthesis | -0.10 (-0.16, -0.03) | 0.0058 | 0.0754 |  |
| adenine and adenosine salvage III | -0.08 (-0.14, -0.02) | 0.0148 | 0.0981 |  |
| starch degradation V | -0.09 (-0.15, -0.03) | 0.0062 | 0.0754 |  |
| Escherichia coli isolate Escherichia coli str. TO124 genome ... | -0.11 (-0.18, -0.05) | 0.0007 | 0.0445 |  |
| pyruvate fermentation to isobutanol (engineered) | -0.12 (-0.18, -0.05) | 0.0008 | 0.0445 |  |
| D-fructuronate degradation | -0.12 (-0.18, -0.05) | 0.0008 | 0.0445 |  |
| purine ribonucleosides degradation | -0.13 (-0.19, -0.06) | 0.0001 | 0.0378 |  |
| Halomonas sp. TD01 Halomonas Bluephagenesis substr. TD01 ... | -0.10 (-0.16, -0.03) | 0.0065 | 0.0754 |  |
| Mycobacterium tuberculosis H37Rv superpathway of pyrimidine ... | -0.11 (-0.18, -0.04) | 0.0018 | 0.0704 |  |
| nitrate reduction VI (assimilatory) | -0.09 (-0.16, -0.03) | 0.0061 | 0.0754 |  |
| phosphatidylglycerol biosynthesis I | -0.09 (-0.15, -0.02) | 0.0153 | 0.0981 |  |
| Enterococcus faecalis ATCC 29200 phosphatidylglycerol ... | -0.09 (-0.15, -0.03) | 0.0061 | 0.0754 |  |
| NAD salvage pathway I (PNC VI cycle) | -0.12 (-0.18, -0.05) | 0.0003 | 0.0379 |  |
| formaldehyde assimilation II (assimilatory RuMP Cycle) | -0.09 (-0.15, -0.03) | 0.0048 | 0.0746 |  |
| tRNA charging | -0.12 (-0.18, -0.06) | 0.0003 | 0.0378 |  |
| L-tryptophan biosynthesis | -0.09 (-0.16, -0.02) | 0.0097 | 0.0866 |  |
| **Longitudinal analysis** |  |  |  |  |
| **Reversed Empirical Dietary Index for Hyperinsulinemia (rEDIH)** | |  |  |  |
| **Pathway** | **PD per SD** | **p value** | **FDR p value** |  |
| L-arginine biosynthesis II (acetyl cycle) | -0.09 (-0.15, -0.02) | 0.0091 | 0.0973 |  |
| Calvin-Benson-Bassham cycle | -0.09 (-0.15, -0.02) | 0.0090 | 0.0973 |  |
| glycogen biosynthesis I (from ADP-D-Glucose) | -0.09 (-0.15, -0.02) | 0.0082 | 0.0973 |  |
| L-methionine biosynthesis I | -0.06 (-0.11, -0.02) | 0.0082 | 0.0973 |  |
| superpathway of S-adenosyl-L-methionine biosynthesis | -0.06 (-0.10, -0.01) | 0.0090 | 0.0973 |  |
| nitrate reduction VI (assimilatory) | -0.08 (-0.13, -0.03) | 0.0030 | 0.0779 |  |
| superpathway of L-methionine biosynthesis | -0.06 (-0.10, -0.01) | 0.0094 | 0.0973 |  |
| L-glutamate and L-glutamine biosynthesis | -0.09 (-0.15, -0.03) | 0.0054 | 0.0939 |  |
| adenosylcobalamin biosynthesis from ... | -0.09 (-0.15, -0.03) | 0.0034 | 0.0779 |  |
| Escherichia coli K-12 substr | -0.08 (-0.15, -0.02) | 0.0078 | 0.0973 |  |
| Superpathway of adenosylcobalamin salvage from cobinamide II | -0.09 (-0.15, -0.03) | 0.0034 | 0.0779 |  |
| GDP-D-glycero-α-D-manno-heptose biosynthesis | -0.09 (-0.15, -0.03) | 0.0041 | 0.0803 |  |
| Superpathway of L-phenylalanine biosynthesis | -0.10 (-0.16, -0.04) | 0.0011 | 0.0694 |  |
| Escherichia coli K-12 substr. MG1655 superpathway of L-tyrosine ... | -0.10 (-0.16, -0.04) | 0.0010 | 0.0694 |  |
| Escherichia coli isolate Escherichia coli str. TO124 genome ... | -0.09 (-0.16, -0.03) | 0.0038 | 0.0784 |  |
| taxadiene biosynthesis (engineered) | -0.09 (-0.15, -0.03) | 0.0046 | 0.0822 |  |
| NAD salvage pathway I (PNC VI cycle) | -0.09 (-0.15, -0.02) | 0.0068 | 0.0965 |  |
| flavin biosynthesis I (bacteria and plants) | -0.08 (-0.14, -0.02) | 0.0062 | 0.0965 |  |
| L-tryptophan biosynthesis | -0.09 (-0.16, -0.03) | 0.0034 | 0.0779 |  |
|  |  |  |  |  |
| **Reversed Empirical Dietary Inflammatory Index (rEDIP)** |  |  |  |  |
| **Pathways** | **PD per SD** | **p value** | **FDR p value** |  |
| fatty acid elongation -- saturated | 0.07 (0.03, 0.12) | 0.0023 | 0.0779 |  |
| superpathway of N-acetylglucosamine | -0.10 (-0.16, -0.04) | 0.0017 | 0.0767 |  |
| superpathway of (Kdo)2-lipid A biosynthesis | 0.06 (0.02, 0.10) | 0.0067 | 0.0965 |  |
| acetylene degradation (anaerobic) | -0.09 (-0.15, -0.02) | 0.0089 | 0.0973 |  |
| Escherichia coli K-12 substr. MG1655 6-hydroxymethyl ... | 0.07 (0.02, 0.12) | 0.0068 | 0.0965 |  |
| Escherichia coli K-12 substr. MG1655 S-adenosyl-L ... | -0.10 (-0.16, -0.03) | 0.0032 | 0.0779 |  |
| Kdo transfer to lipid IVA (Chlamydia) | 0.06 (0.02, 0.10) | 0.0068 | 0.0965 |  |
| GDP-D-glycero-α-D-manno-heptose biosynthesis | -0.11 (-0.17, -0.05) | 0.0006 | 0.0694 |  |
| Stenotrophomonas maltophilia K279a pyrimidine ... | 0.09 (0.04, 0.14) | 0.0007 | 0.0694 |  |
| 6-hydroxymethyl-dihydropterin diphosphate biosynthesis III (Chlamydia) | 0.07 (0.03, 0.12) | 0.0024 | 0.0779 |  |
| Pathway Summary from MetaCyc | -0.09 (-0.16, -0.03) | 0.0045 | 0.0822 |  |
| nitrate reduction VI (assimilatory) | -0.08 (-0.14, -0.03) | 0.0017 | 0.0767 |  |
| NAD salvage pathway I (PNC VI cycle) | -0.09 (-0.16, -0.03) | 0.0036 | 0.0784 |  |
|  |  |  |  |  |
| **Healthy Eating Index (HEI-2020)** |  |  |  |  |
| **Pathways** | **PD per SD** | **p value** | **FDR p value** |  |
| glycolysis III (from glucose) | -0.09 (-0.16, -0.03) | 0.0055 | 0.0939 |  |
| Calvin-Benson-Bassham cycle | -0.09 (-0.15, -0.02) | 0.0081 | 0.0973 |  |
| Escherichia coli K-12 substr. MG1655 L-lysine biosynthesis I | -0.09 (-0.16, -0.03) | 0.0037 | 0.0784 |  |
| dTDP-L-rhamnose biosynthesis | -0.10 (-0.17, -0.04) | 0.0021 | 0.0779 |  |
| superpathway of N-acetylglucosamine | -0.11 (-0.17, -0.05) | 0.0005 | 0.0694 |  |
| Glycogen degradation II | -0.11 (-0.17, -0.05) | 0.0005 | 0.0694 |  |
| acetylene degradation (anaerobic) | -0.09 (-0.16, -0.03) | 0.0039 | 0.0785 |  |
| Escherichia coli K-12 substr. MG1655 S-adenosyl-L ... | -0.12 (-0.18, -0.05) | 0.0003 | 0.0694 |  |
| sucrose degradation III (sucrose invertase) | -0.10 (-0.16, -0.03) | 0.0029 | 0.0779 |  |
| D-galactose degradation I (Leloir pathway) | -0.11 (-0.17, -0.04) | 0.0016 | 0.0767 |  |
| GDP-D-glycero-α-D-manno-heptose biosynthesis | -0.11 (-0.17, -0.05) | 0.0004 | 0.0694 |  |
| adenine and adenosine salvage III | -0.09 (-0.16, -0.02) | 0.0086 | 0.0973 |  |
| starch degradation V | -0.10 (-0.16, -0.03) | 0.0044 | 0.0822 |  |
| Escherichia coli K-12 substr. MG1655 thiamine diphosphate salvage II | -0.09 (-0.16, -0.03) | 0.0043 | 0.0822 |  |
| purine ribonucleosides degradation | -0.10 (-0.16, -0.03) | 0.0037 | 0.0784 |  |
| Halomonas sp. TD01 Halomonas Bluephagenesis substr. TD01 ... | -0.09 (-0.16, -0.03) | 0.0033 | 0.0779 |  |
| Mycobacterium tuberculosis H37Rv superpathway of pyrimidine ... | -0.10 (-0.16, -0.03) | 0.0030 | 0.0779 |  |
| nitrate reduction VI (assimilatory) | -0.07 (-0.12, -0.02) | 0.0055 | 0.0939 |  |
| phosphatidylglycerol biosynthesis I | -0.09 (-0.16, -0.03) | 0.0064 | 0.0965 |  |
| Enterococcus faecalis ATCC 29200 phosphatidylglycerol ... | -0.09 (-0.16, -0.03) | 0.0064 | 0.0965 |  |
| NAD salvage pathway I (PNC VI cycle) | -0.11 (-0.18, -0.05) | 0.0004 | 0.0694 |  |

| **Supplementary Table 7. Associations of the Reversed Empirical Dietary Index for Hyperinsulinemia (rEDIH) score with circulating biomarkers of renal function, insulin response and lipids** | | | | | | | | |  |
| --- | --- | --- | --- | --- | --- | --- | --- | --- | --- |
|  |  | Highest insulinemic potential of the diet | rEDIH Quintiles (Q) |  |  | Lowest insulinemic potential of the diet | |  |  |
| Biomarkers | Statistical model | Quintile 1 | Quintile 2 | Quintile 3 | Quintile 4 | Quintile 5 | Percentage difference:  Q5-Q1 | Percent difference per 1 SD increment in dietary score | P-value |
| **Renal function biomarkers** |  |  |  |  |  |  |  |  |  |
| Serum creatinine (mg/dL) | ^b^MV model | 0.99(0.96,1.01) | 0.99(0.97,1.02) | 0.98(0.96,1.01) | 0.98(0.95,1) | 0.96(0.93,0.98) | -3.13(-5.56, -0.7) | -1.24 (-1.86, -0.62) | 0.0001 |
| n = 4421 | ^c^MV+ BMI | 0.99(0.96,1.01) | 0.99(0.97,1.02) | 0.99(0.96,1.01) | 0.98(0.95,1.01) | 0.96(0.93,0.99) | -2.59(-5.05, -0.12) | -1.08 (-1.71, -0.45) | 0.0008 |
|  |  |  |  |  |  |  |  |  |  |
| Urine Creatinine (mg/dL) | ^b^MV model | 114.79(98.54,133.72) | 114.88(98.86,133.49) | 111.29(95.33,129.91) | 105.79(90.66,123.46) | 96.28(82.46,112.42) | -17.58(-31.14, -4.03) | -5.95 (-9.42, -2.48) | 0.0008 |
| n = 1276 | ^c^MV+ BMI | 115.36(99.07,134.33) | 115.42(99.36,134.08) | 112.68(96.53,131.52) | 107.43(92.06,125.37) | 98.62(84.41,115.22) | -15.68(-29.31, -2.05) | -5.30 (-8.79, -1.81) | 0.0029 |
|  |  |  |  |  |  |  |  |  |  |
| Serum cystatin C (mg/L) | ^b^MV model | 1.03(0.99,1.08) | 1.05(1,1.1) | 1.09(1.04,1.14) | 1.07(1.02,1.12) | 1.05(1,1.1) | 1.66(-2.44,5.77) | 0.39 (-0.67, 1.44) | 0.4713 |
| n = 1293 | ^c^MV+ BMI | 1.04(0.99,1.08) | 1.05(1.01,1.1) | 1.09(1.04,1.15) | 1.08(1.03,1.13) | 1.06(1.01,1.11) | 2.51(-1.61,6.63) | 0.66 (-0.40, 1.71) | 0.2219 |
|  |  |  |  |  |  |  |  |  |  |
| Serum sodium (mmol/L) | ^b^MV model | 140.9(140.55,141.24) | 140.97(140.62,141.32) | 141.02(140.67,141.37) | 141(140.65,141.35) | 141.05(140.7,141.39) | 0.11(-0.11,0.32) | 1.00(0.99, 1.00) | 0.3401 |
| n = 4417 | ^c^MV+ BMI | 140.89(140.54,141.23) | 140.98(140.63,141.32) | 141.03(140.69,141.39) | 141.02(140.67,141.37) | 141.08(140.73,141.43) | 0.14(-0.08,0.36) | 1.00(0.99, 1.00) | 0.1893 |
|  |  |  |  |  |  |  |  |  |  |
| Urine sodium (mmol/L) | ^b^MV model | 112.4(101.12,123.69) | 114.72(103.57,125.88) | 104.84(93.42,116.25) | 102.97(91.57,114.37) | 95.06(83.6,106.53) | -15.43(-26.99, -5.97) | -5.73(-8.23, -3.22) | <.0001 |
| n = 1271 | ^c^MV+ BMI | 112.87(101.64,124.09) | 115.18(104.08,126.27) | 106.05(94.68,117.41) | 104.54(93.17,115.9) | 97.51(86.04,108.98) | -13.61(-24.92, -4.34) | -5.04(-7.56, -2.53) | <.0001 |
|  |  |  |  |  |  |  |  |  |  |
| Serum potassium (mmol/L) | ^b^MV model | 4.19(4.15,4.24) | 4.2(4.16,4.25) | 4.18(4.13,4.23) | 4.18(4.13,4.22) | 4.17(4.13,4.22) | -0.47(-1.42,0.47) | -0.23 (-0.47, 0.01) | 0.063 |
| n = (4417) | ^c^MV+ BMI | 4.19(4.15,4.24) | 4.2(4.16,4.25) | 4.18(4.13,4.22) | 4.18(4.13,4.22) | 4.17(4.12,4.22) | -0.54(-1.5,0.42) | -0.25 (-0.50, -0.01) | 0.0451 |
|  |  |  |  |  |  |  |  |  |  |
| Urine potassium (mmol/L) | ^b^MV model | 48.75(42.76,55.59) | 46.57(40.9,53.04) | 45.6(39.88,52.15) | 43.15(37.74,49.32) | 45.84(40.1,52.39) | -6.17(-18.02,5.69) | -2.18 (-5.21, 0.85) | 0.1591 |
| n = 1161 | ^c^MV+ BMI | 48.9(42.89,55.75) | 46.72(41.03,53.19) | 46(40.23,52.6) | 43.59(38.13,49.84) | 46.6(40.75,53.3) | -4.82(-16.76,7.13) | -1.74 (-4.79, 1.32) | 0.2651 |
|  |  |  |  |  |  |  |  |  |  |
| Serum phosphate (mg/dL) | ^b^MV model | 3.13(3.07,3.19) | 3.09(3.03,3.14) | 3.11(3.06,3.17) | 3.08(3.02,3.14) | 3.1(3.04,3.16) | -0.95(-2.56,0.66) | -0.47 (-0.88, -0.06) | 0.0253 |
| n = 4421 | ^c^MV+ BMI | 3.13(3.07,3.18) | 3.09(3.03,3.14) | 3.12(3.06,3.18) | 3.08(3.02,3.14) | 3.1(3.05,3.16) | -0.7(-2.33,0.93) | -0.39 (-0.81, 0.02) | 0.0636 |
|  |  |  |  |  |  |  |  |  |  |
| Urine phosphate (mg/dL) | ^b^MV model | 51.64(43.4,61.45) | 51.32(43.25,60.91) | 49.62(41.6,59.17) | 45.76(38.34,54.61) | 41.66(34.94,49.68) | -21.48(-36.84, -6.12) | -7.79 (-11.72, -3.86) | 0.0001 |
| n = 1269 | ^c^MV+ BMI | 52.22(43.96,62.04) | 51.85(43.76,61.44) | 50.96(42.79,60.69) | 47.33(39.71,56.42) | 43.76(36.72,52.16) | -17.67(-33, -2.34) | -6.51 (-10.44, -2.59) | 0.0012 |
| **Lipid biomarkers** |  |  |  |  |  |  |  |  |  |
| High density lipoprotein (mg/dL) | ^b^MV model | 45.38(43.73,47.08) | 46.24(44.57,47.98) | 46.26(44.57,48.02) | 46.74(45.04,48.52) | 47.43(45.7,49.21) | 4.42(1.21,7.62) | 1.01 (0.19, 1.83) | 0.0154 |
| n= (4421) | ^c^MV+ BMI | 45.75(44.15,47.42) | 45.94(44.33,47.6) | 45.62(44.00,47.29) | 45.97(44.34,47.66) | 46.01(44.39,47.70) | 0.57(-2.58,3.71) | -0.21 (-1.01, 0.59) | 0.6104 |
|  |  |  |  |  |  |  |  |  |  |
| Low density lipoprotein (mg/dl) | ^b^MV model | 116.28(112.25,120.31) | 117.43(113.41,121.46) | 118.3(114.24,122.37) | 115.01(110.94,119.07) | 116.58(112.54,120.62) | 0.26(-2.85,3.16) | -0.31(-1.09,0.47) | 0.4392 |
| n = (4420) | ^c^MV+ BMI | 116.37(112.34,120.4) | 117.36(113.34,121.38) | 118.15(114.08,122.21) | 114.82(110.75,118.89) | 116.25(112.19,120.30) | -0.10(-3.27,2.85) | -0.43(-1.23,0.36) | 0.2840 |
|  |  |  |  |  |  |  |  |  |  |
| Triglycerides (mg/dl) | ^b^MV model | 136(127.03,145.59) | 132.84(124.1,142.19) | 131.96(123.20,141.34) | 131.39(122.66,140.73) | 122.85(114.74,131.54) | -10.17(-16.08, -4.25) | -2.64 (-4.15, -1.13) | 0.0006 |
| n = (4421) | ^c^MV+ BMI | 134.25(125.6,143.5) | 134.22(125.59,143.45) | 134.88(126.12,144.26) | 134.87(126.11,144.25) | 128.80(120.44,137.73) | -4.15(-10.01,1.72) | -0.73 (-2.23, 0.76) | 0.3365 |
|  |  |  |  |  |  |  |  |  |  |
| Total Cholesterol - calculated  (mg/dL) | ^b^MV model | 196.57(192.11,201.03) | 197.32(192.87,201.77) | 198.55(194.06,203.04) | 195.96(191.46,200.45) | 195.32(190.85,199.79) | -0.63(-2.66,1.31) | -0.39(-0.9,0.13) | 0.1395 |
| n = (4420) | ^c^MV+ BMI | 196.69(192.23,201.15) | 197.23(192.78,201.68) | 198.35(193.86,202.85) | 195.72(191.22,200.22) | 194.89(190.41,199.38) | -0.91(-2.98,1.06) | -0.48(-1,0.04) | 0.0702 |
|  |  |  |  |  |  |  |  |  |  |
| TG/HDL^d^ ratio | ^b^MV model | 3(2.73,3.29) | 2.87(2.62,3.16) | 2.85(2.59,3.14) | 2.81(2.56,3.09) | 2.59(2.36,2.85) | -14.58(-22.75, -6.41) | -3.65 (-5.74, -1.57) | 0.0006 |
| n= (4421) | ^c^MV+ BMI | 2.93(2.68,3.21) | 2.92(2.67,3.2) | 2.96(2.7,3.24) | 2.93(2.68,3.22) | 2.8(2.55,3.07) | -4.71(-12.73,3.3) | -0.53 (-2.57, 1.52) | 0.6146 |
|  |  |  |  |  |  |  |  |  |  |
| TC/HDL^d^ ratio | ^b^MV model | 4.25(4.09,4.42) | 4.19(4.03,4.35) | 4.22(4.06,4.38) | 4.12(3.96,4.28) | 4.05(3.9,4.21) | -4.78(-8.11, -1.45) | -1.31 (-2.16, -0.46) | 0.0026 |
| n = (4420) | ^c^MV+ BMI | 4.22(4.06,4.38) | 4.22(4.06,4.38) | 4.27(4.11,4.44) | 4.18(4.03,4.34) | 4.17(4.01,4.33) | -1.18(-4.48,2.11) | -0.17 (-1.02, 0.67) | 0.6838 |
| **Insulin response biomarkers** |  |  |  |  |  |  |  |  |  |
| Fasting glucose(mg/dL) | ^b^MV model | 99.66(98.25,101.09) | 98.09(96.7,99.5) | 97.97(96.57,99.4) | 98.62(97.2,100.06) | 97.76(96.36,99.18) | -1.93(-3.14, -0.71) | -0.60 (-0.91, -0.29) | 0.0002 |
| n = 3701 | ^c^MV+ BMI | 99.32(97.96,100.7) | 98.28(96.93,99.65) | 98.39(97.03,99.77) | 99.08(97.7,100.48) | 98.74(97.36,100.13) | -0.59(-1.78,0.61) | -0.18 (-0.49, 0.12) | 0.2456 |
|  |  |  |  |  |  |  |  |  |  |
| Fasting insulin(uIU/mL) | ^b^MV model | 7.79(7.19,8.45) | 7.38(6.80,8.00) | 6.98(6.43,7.57) | 6.79(6.26,7.37) | 6.21(5.73,6.74) | -22.68(-29.54, -15.81) | -6.77 (-8.53, -5.01) | <.0001 |
| n = 3701 | ^c^MV+ BMI | 7.50(6.99,8.05) | 7.54(7.03,8.09) | 7.32(6.82,7.86) | 7.15(6.66,7.68) | 6.94(6.47,7.45) | -7.73(-13.78, -1.68) | -2.08 (-3.63, -0.54) | 0.0083 |
|  |  |  |  |  |  |  |  |  |  |
| HOMA-IR^d^ | ^b^MV model | 1.92(1.76,2.09) | 1.79(1.64,1.95) | 1.69(1.55,1.84) | 1.65(1.52,1.81) | 1.50(1.37,1.64) | -24.6(-31.97, -17.24) | -7.37 (-9.25, -5.48) | <.0001 |
| n = 3701 | ^c^MV+ BMI | 1.84(1.71,1.98) | 1.83(1.70,1.97) | 1.78(1.65,1.92) | 1.75(1.62,1.89) | 1.69(1.57,1.83) | -8.31(-14.77, -1.86) | -2.26 (-3.91, -0.61) | 0.0072 |
| ^a^Values are absolute back-transformed biomarker concentrations (beta coefficients) since values were naturally log-transformed prior to analysis, and the p-value of < 0.05 represent statistically significant findings.  Beta coefficients were derived from ^b^MV (multivariable) adjusted, and ^c^multivariable + BMI (body mass index) adjusted linear regression models.  Multivariable linear regression models were adjusted for total energy intake, race/ethnicity (White, African American, Asian, Hispanic, others), marital status (married, single, divorced/separated, widowed), education (less than high school, high school, college and higher), physical activity (continuous), smoking status (never, past, current), pack-years of smoking (continuous), number of nutritional supplements used (continuous), total alcohol intake (continuous, servings/day), aspirin/other NSAID use (yes vs no), baseline comorbidity score conditions (continuous).  ^d^TG, triglycerides; HDL, high-density lipoprotein; TC, total cholesterol; HOMA-IR, homeostatic model assessment of insulin resistance. | | | | | | | | | |

| **Supplementary Table 8. Associations of the Reversed Empirical Dietary Inflammatory Pattern (rEDIP) score with circulating biomarkers of renal function, insulin response and lipids** | | | | | | | | | |
| --- | --- | --- | --- | --- | --- | --- | --- | --- | --- |
|  |  | Highest inflammatory potential of the diet | rEDIP Quintiles (Q) |  |  | Lowest inflammatory potential of the diet | |  |  |
| Biomarkers | Statistical model | Quintile 1 | Quintile 2 | Quintile 3 | Quintile 4 | Quintile 5 | Percentage difference: Q5-Q1 | Percent difference per 1 SD increment in dietary score | P-value |
| **Renal function biomarkers** |  |  |  |  |  |  |  |  |  |
| Serum creatinine (mg/dL) | ^b^MV model | 0.98(0.96,1.01) | 0.99(0.96,1.02) | 0.98(0.95,1.00) | 0.98(0.95,1.01) | 0.96(0.94,0.99) | -2.14(-4.67,0.38) | -1.10 (-1.75, -0.46) | 0.0008 |
| n = 4421 | ^c^MV+ BMI | 0.98(0.96,1.01) | 0.99(0.96,1.02) | 0.98(0.95,1.01) | 0.98(0.95,1.01) | 0.97(0.94,0.99) | -1.89(-4.42,0.63) | -1.00 (-1.65, -0.35) | 0.0024 |
|  |  |  |  |  |  |  |  |  |  |
| Urine Creatinine (mg/dL) | ^b^MV model | 119.84(103.43,138.84) | 110.54(94.92,128.75) | 103.15(88.24,120.58) | 98.99(84.38,116.13) | 95.45(81.51,111.78) | -22.75(-37.07, -8.44) | -7.99 (-11.68, -4.30) | <.0001 |
| n = 1276 | ^c^MV+ BMI | 120.86(104.36,139.98) | 111.59(95.85,129.93) | 104.77(89.63,122.46) | 100.61(85.77,118.02) | 97.17(82.98,113.79) | -21.82(-36.11, -7.52) | -7.61 (-11.30, -3.93) | <.0001 |
|  |  |  |  |  |  |  |  |  |  |
| Serum cystatin C (mg/L) | ^b^MV model | 1.05(1.00,1.10) | 1.06(1.01,1.11) | 1.08(1.03,1.13) | 1.06(1.01,1.11) | 1.05(1.00,1.10) | -0.28(-4.63,4.07) | -0.59 (-1.71, 0.54) | 0.3065 |
| n = 1293 | ^c^MV+ BMI | 1.05(1.01,1.10) | 1.06(1.02,1.12) | 1.09(1.04,1.14) | 1.07(1.02,1.12) | 1.05(1.00,1.11) | 0.11(-4.22,4.45) | -0.44 (-1.56, 0.68) | 0.4381 |
|  |  |  |  |  |  |  |  |  |  |
| Serum sodium (mmol/L) | ^b^MV model | 140.90(140.57,141.24) | 141.13(140.78,141.48) | 141.01(140.65,141.36) | 141(140.64,141.35) | 140.93(140.58,141.29) | 0.02(-0.2,0.24) | -0.02 (-0.08, 0.03) | 0.4029 |
| n = 4417 | ^c^MV+ BMI | 140.91(140.57,141.25) | 141.14(140.79,141.49) | 141.03(140.67,141.38) | 141.02(140.66,141.37) | 140.95(140.6,141.31) | 0.03(-0.19,0.25) | -0.02 (-0.08, 0.04) | 0.5084 |
|  |  |  |  |  |  |  |  |  |  |
| Urine sodium (mmol/L) | ^b^MV model | 113.12(102.14,124.09) | 109.82(98.5,121.15) | 104.65(93.05,116.24) | 99.49(87.63,111.35) | 96.02(84.31,107.73) | -15.11(-27.08, -5.26) | -6.2(-8.87, -3.53) | <.0001 |
| n = 1271 | ^c^MV+ BMI | 113.87(102.96,124.77) | 110.89(99.64,122.15) | 106.26(94.73,117.8) | 101.09(89.29,112.89) | 97.99(86.32,109.66) | -13.94(-25.63, -4.3) | -5.8(-8.46, -3.14) | <.0001 |
|  |  |  |  |  |  |  |  |  |  |
| Serum potassium (mmol/L) | ^b^MV model | 4.20(4.15,4.24) | 4.18(4.13,4.22) | 4.18(4.13,4.23) | 4.18(4.13,4.23) | 4.18(4.13,4.22) | -0.54(-1.52,0.45) | -0.20 (-0.45, 0.05) | 0.1212 |
| n = (4417) | ^c^MV+ BMI | 4.20(4.15,4.24) | 4.18(4.13,4.22) | 4.18(4.13,4.23) | 4.18(4.13,4.22) | 4.17(4.13,4.22) | -0.56(-1.54,0.43) | -0.21 (-0.46, 0.04) | 0.1059 |
|  |  |  |  |  |  |  |  |  |  |
| Urine potassium (mmol/L) | ^b^MV model | 48.12(42.35,54.68) | 46.27(40.54,52.81) | 44.81(39.19,51.24) | 43.82(38.17,50.31) | 44.52(38.81,51.06) | -7.79(-20.3,4.72) | -2.65 (-5.87, 0.57) | 0.1075 |
| n = 1161 | ^c^MV+ BMI | 48.39(42.59,54.98) | 46.52(40.76,53.09) | 45.24(39.57,51.74) | 44.28(38.57,50.84) | 45.05(39.27,51.68) | -7.15(-19.66,5.36) | -2.40 (-5.63, 0.82) | 0.1439 |
|  |  |  |  |  |  |  |  |  |  |
| Serum phosphate (mg/dL) | ^b^MV model | 3.11(3.05,3.16) | 3.09(3.03,3.14) | 3.11(3.05,3.17) | 3.1(3.04,3.16) | 3.1(3.05,3.16) | -0.07(-1.73,1.6) | -0.05 (-0.47, 0.38) | 0.8277 |
| n = 4421 | ^c^MV+ BMI | 3.11(3.05,3.16) | 3.09(3.03,3.15) | 3.11(3.06,3.17) | 3.1(3.04,3.16) | 3.11(3.05,3.17) | 0.05(-1.62,1.72) | -0.005(-0.43, 0.43) | 0.9986 |
|  |  |  |  |  |  |  |  |  |  |
| Urine phosphate (mg/dL) | ^b^MV model | 53.4(45.12,63.2) | 47.82(40.19,56.89) | 46.15(38.58,55.2) | 44.04(36.7,52.86) | 42.19(35.22,50.54) | -23.55(-39.84, -7.26) | -8.02 (-12.22, -3.83) | 0.0002 |
| n = 1269 | ^c^MV+ BMI | 54.37(46.02,64.24) | 48.81(41.1,57.97) | 47.69(39.93,56.96) | 45.57(38.02,54.61) | 43.8(36.61,52.39) | -21.63(-37.77, -5.5) | -7.29 (-11.45, -3.13) | 0.0006 |
| **Lipid biomarkers** |  |  |  |  |  |  |  |  |  |
| High density lipoprotein (mg/dL) | ^b^MV model | 44.84(43.26,46.49) | 46.36(44.68,48.1) | 46.85(45.13,48.64) | 47.38(45.63,49.19) | 48.41(46.62,50.27) | 7.66(4.34,10.97) | 2.23 (1.39, 3.08) | <.0001 |
| n= (4421) | ^c^MV+ BMI | 44.71(43.18,46.29) | 45.89(44.27,47.56) | 46.12(44.48,47.83) | 46.53(44.87,48.26) | 47.51(45.8,49.27) | 6.07(2.86,9.29) | 1.58 (0.76, 2.41) | 0.0002 |
|  |  |  |  |  |  |  |  |  |  |
| Low density lipoprotein (mg/dl) | ^b^MV model | 115.91(111.97,119.85) | 118.77(114.72,122.82) | 115.95(111.84,120.05) | 117.26(113.15,121.38) | 116.06(111.93,120.18) | 0.13(-3.11,3.15) | -0.07(-0.88,0.74) | 0.8629 |
| n = (4420) | ^c^MV+ BMI | 115.88(111.94,119.81) | 118.66(114.61,122.71) | 115.78(111.67,119.89) | 117.07(112.94,121.19) | 115.85(111.72,119.98) | -0.02(-3.27,3.01) | -0.13(-0.95,0.68) | 0.7471 |
|  |  |  |  |  |  |  |  |  |  |
| Triglycerides (mg/dl) | ^b^MV model | 139.45(130.49,149.03) | 132.43(123.68,141.8) | 127.15(118.65,136.27) | 127.02(118.5,136.16) | 119.81(111.76,128.44) | -15.18(-21.3, -9.05) | -4.63 (-6.20, -3.07) | <.0001 |
| n = (4421) | ^c^MV+ BMI | 140.12(131.32,149.52) | 134.59(125.89,143.88) | 130.32(121.79,139.44) | 130.68(122.09,139.87) | 123.43(115.31,132.13) | -12.68(-18.68, -6.69) | -3.61 (-5.14, -2.07) | <.0001 |
|  |  |  |  |  |  |  |  |  |  |
| Total Cholesterol - calculated  (mg/dL) | ^b^MV model | 196.37(192.02,200.73) | 199.15(194.68,203.63) | 195.54(191,200.07) | 197.17(192.62,201.73) | 195.05(190.49,199.61) | -0.67(-2.78,1.34) | -0.32(-0.85,0.22) | 0.2456 |
| n = (4420) | ^c^MV+ BMI | 196.33(191.98,200.68) | 199.02(194.54,203.49) | 195.33(190.78,199.87) | 196.93(192.37,201.49) | 194.8(190.23,199.36) | -0.78(-2.89,1.24) | -0.36(-0.9,0.17) | 0.1843 |
|  |  |  |  |  |  |  |  |  |  |
| TG/HDL^d^ ratio | ^b^MV model | 3.11(2.84,3.41) | 2.86(2.60,3.14) | 2.71(2.47,2.99) | 2.68(2.44,2.95) | 2.47(2.25,2.72) | -22.83(-31.28, -14.39) | -6.87 (-9.03, -4.71) | <.0001 |
| n= (4421) | ^c^MV+ BMI | 3.13(2.87,3.42) | 2.93(2.68,3.21) | 2.83(2.58,3.10) | 2.81(2.56,3.08) | 2.60(2.37,2.85) | -18.76(-26.94, -10.57) | -5.19 (-7.29, -3.09) | <.0001 |
|  |  |  |  |  |  |  |  |  |  |
| TC/HDL^d^ ratio | ^b^MV model | 4.30(4.14,4.46) | 4.22(4.06,4.39) | 4.10(3.94,4.26) | 4.09(3.93,4.25) | 3.96(3.81,4.12) | -8.05(-11.50, -4.60) | -2.47 (-3.36, -1.59) | <.0001 |
| n = (4420) | ^c^MV+ BMI | 4.31(4.15,4.47) | 4.26(4.11,4.43) | 4.16(4.01,4.32) | 4.16(4,4.32) | 4.03(3.88,4.19) | -6.57(-9.94, -3.21) | -1.86 (-2.73, -1.00) | <.0001 |
| **Insulin response biomarkers** |  |  |  |  |  |  |  |  |  |
| Fasting glucose(mg/dL) | ^b^MV model | 98.68(97.31,100.07) | 98.48(97.07,99.91) | 98.58(97.14,100.03) | 97.79(96.37,99.24) | 98.45(97.01,99.91) | -0.23(-1.5,1.04) | -0.20 (-0.52, 0.12) | 0.2219 |
| n = 3701 | ^c^MV+ BMI | 98.69(97.36,100.03) | 98.74(97.37,100.12) | 99.11(97.71,100.53) | 98.38(96.99,99.78) | 99.06(97.66,100.48) | 0.38(-0.85,1.60) | 0.03 (-0.28, 0.34) | 0.8545 |
|  |  |  |  |  |  |  |  |  |  |
| Fasting insulin(uIU/mL) | ^b^MV model | 7.51(6.94,8.13) | 7.27(6.70,7.89) | 7.05(6.49,7.67) | 6.59(6.07,7.16) | 6.32(5.82,6.87) | -17.24(-24.41, -10.07) | -6.02 (-7.85, -4.19) | <.0001 |
| n = 3701 | ^c^MV+ BMI | 7.52(7.02,8.05) | 7.48(6.97,8.03) | 7.49(6.97,8.05) | 7.04(6.55,7.57) | 6.77(6.30,7.27) | -10.51(-16.72, -4.30) | -3.46 (-5.05, -1.87) | <.0001 |
|  |  |  |  |  |  |  |  |  |  |
| HOMA-IRd | ^b^MV model | 1.83(1.68,1.99) | 1.77(1.62,1.93) | 1.72(1.57,1.88) | 1.59(1.46,1.74) | 1.54(1.41,1.68) | -17.47(-25.17, -9.77) | -6.22 (-8.19, -4.25) | <.0001 |
| n = 3701 | ^c^MV+ BMI | 1.83(1.70,1.97) | 1.82(1.69,1.97) | 1.83(1.70,1.98) | 1.71(1.58,1.85) | 1.66(1.53,1.79) | -10.13(-16.76, -3.51) | -3.43 (-5.12, -1.73) | <.0001 |
| ^a^Values are absolute back-transformed biomarker concentrations (beta coefficients) since values were naturally log-transformed prior to analysis, and the p-value of < 0.05 represent statistically significant findings.  Beta coefficients were derived from ^b^MV (multivariable) adjusted, and ^c^multivariable + BMI (body mass index) adjusted linear regression models.  Multivariable linear regression models were adjusted for total energy intake, race/ethnicity (White, African American, Asian, Hispanic, others), marital status (married, single, divorced/separated, widowed), education (less than high school, high school, college and higher), physical activity (continuous), smoking status (never, past, current), pack-years of smoking (continuous), number of nutritional supplements used (continuous), total alcohol intake (continuous, servings/day), aspirin/other NSAID use (yes vs no), baseline comorbidity score conditions (continuous).  ^d^TG, triglycerides; HDL, high-density lipoprotein; TC, total cholesterol; HOMA-IR, homeostatic model assessment of insulin resistance. | | | | | | | | | |

| **Supplementary Table 9. Associations of the Healthy Eating Index (HEI)-2020 score with circulating biomarkers of renal function, insulin response and lipids** | | | | | | | | | |
| --- | --- | --- | --- | --- | --- | --- | --- | --- | --- |
|  |  | Lowest dietary quality | HEI-2020 Quintiles |  |  | Highest dietary quality |  |  |  |
| Biomarkers | Statistical model | Quintile 1 | Quintile 2 | Quintile 3 | Quintile 4 | Quintile 5 | Percentage difference: Q5-Q1 | Percent difference per 1 SD increment in dietary score | P-value |
| **Renal function biomarkers** |  |  |  |  |  |  |  |  |  |
| Serum creatinine (mg/dL) | ^b^MV model | 0.99(0.96,1.02) | 0.98(0.95,1.01) | 0.97(0.95,1) | 0.98(0.96,1.01) | 0.97(0.94,0.99) | -2.38(-4.88,0.12) | -0.85 (-1.49, -0.21) | 0.0094 |
| n = 4421 | ^c^MV+ BMI | 0.99(0.96,1.02) | 0.98(0.95,1.01) | 0.97(0.95,1) | 0.99(0.96,1.01) | 0.97(0.94,1) | -2.06(-4.57,0.44) | -0.74 (-1.38, -0.09) | 0.0249 |
|  |  |  |  |  |  |  |  |  |  |
| Urine Creatinine (mg/dL) | ^b^MV model | 122.26(105.26,142.01) | 107.91(92.75,125.54) | 112.21(96.16,130.94) | 104.35(89.51,121.66) | 93.39(79.95,109.08) | -26.94(-40.63, -13.24) | -10.97 (-14.47, -7.46) | <.0001 |
| n = 1276 | ^c^MV+ BMI | 123.95(106.73,143.94) | 108.60(93.38,126.30) | 113.11(96.97,131.94) | 105.49(90.52,122.95) | 95.47(81.71,111.54) | -26.11(-39.77, -12.44) | -10.61 (-14.12, -7.11) | <.0001 |
|  |  |  |  |  |  |  |  |  |  |
| Serum cystatin C (mg/L) | ^b^MV model | 1.08(1.03,1.13) | 1.04(1,1.09) | 1.05(1,1.10) | 1.05(1.01,1.11) | 1.04(0.99,1.09) | -3.26(-7.44,0.91) | -0.85 (-1.92, 0.23) | 0.1224 |
| n = 1293 | ^c^MV+ BMI | 1.08(1.04,1.13) | 1.05(1,1.10) | 1.05(1,1.10) | 1.06(1.01,1.11) | 1.05(1.00,1.10) | -2.93(-7.09,1.22) | -0.71 (-1.78, 0.36) | 0.1918 |
|  |  |  |  |  |  |  |  |  |  |
| Serum sodium (mmol/L) | ^b^MV model | 141.02(140.68,141.37) | 141.09(140.75,141.44) | 141.09(140.75,141.44) | 140.92(140.57,141.27) | 140.75(140.4,141.10) | -0.19(-0.41,0.03) | -0.08 (-0.14, -0.03) | 0.0042 |
| n = 4417 | ^c^MV+ BMI | 141.03(140.68,141.37) | 141.10(140.75,141.44) | 141.10(140.75,141.45) | 140.93(140.58,141.28) | 140.77(140.42,141.13) | -0.18(-0.40,0.04) | -0.08 (-0.13, -0.02) | 0.0075 |
|  |  |  |  |  |  |  |  |  |  |
| Urine sodium (mmol/L) | ^b^MV model | 112.52(101.38,123.66) | 112.04(100.84,123.24) | 110.82(99.38,122.25) | 101.69(90.31,113.07) | 92.91(81.41,104.42) | -17.42(-29.29, -7.70) | -7.60(-10.16, -5.05) | <.0001 |
| n = 1271 | ^c^MV+ BMI | 113.87(102.78,124.96) | 112.65(101.52,123.78) | 111.6(100.24,122.97) | 102.8(91.48,114.12) | 95.13(83.65,106.62) | -16.46(-28.00, -6.97) | -7.22(-9.76, -4.67) | <.0001 |
|  |  |  |  |  |  |  |  |  |  |
| Serum potassium (mmol/L) | ^b^MV model | 4.18(4.13,4.22) | 4.19(4.14,4.23) | 4.18(4.14,4.23) | 4.19(4.15,4.24) | 4.18(4.14,4.23) | 0.15(-0.82,1.12) | 0.07 (-0.18, 0.32) | 0.5933 |
| n = 4417 | ^c^MV+ BMI | 4.18(4.13,4.22) | 4.19(4.14,4.23) | 4.18(4.14,4.23) | 4.19(4.15,4.24) | 4.18(4.14,4.23) | 0.13(-0.85,1.1) | 0.06 (-0.19, 0.31) | 0.6397 |
|  |  |  |  |  |  |  |  |  |  |
| Urine potassium (mmol/L) | ^b^MV model | 46.46(40.80,52.91) | 44.39(38.89,50.66) | 47.16(41.27,53.88) | 48.41(42.38,55.30) | 44.79(39.13,51.27) | -3.66(-15.71,8.38) | -1.74 (-4.84, 1.37) | 0.2737 |
| n = 1161 | ^c^MV+ BMI | 46.86(41.15,53.36) | 44.60(39.09,50.89) | 47.39(41.48,54.14) | 48.76(42.69,55.70) | 45.45(39.69,52.05) | -3.05(-15.09,9.00) | -1.48 (-4.59, 1.63) | 0.3503 |
|  |  |  |  |  |  |  |  |  |  |
| Serum phosphate (mg/dL) | ^b^MV model | 3.10(3.04,3.15) | 3.10(3.04,3.15) | 3.11(3.05,3.17) | 3.12(3.06,3.18) | 3.09(3.03,3.14) | -0.36(-2.01,1.29) | -0.00 (-0.43, 0.42) | 0.9880 |
| n = 4421 | ^c^MV+ BMI | 3.10(3.04,3.16) | 3.10(3.04,3.15) | 3.11(3.06,3.17) | 3.12(3.06,3.18) | 3.09(3.03,3.15) | -0.22(-1.88,1.44) | 0.05 (-0.38, 0.48) | 0.8183 |
|  |  |  |  |  |  |  |  |  |  |
| Urine phosphate (mg/dL) | ^b^MV model | 54.48(45.92,64.63) | 49.20(41.39,58.48) | 48.79(40.93,58.17) | 46.52(39.07,55.39) | 39.41(33.03,47.04) | -32.36(-47.88, -16.84) | -12.63 (-16.61, -8.65) | <.0001 |
| n = 1269 | ^c^MV+ BMI | 56.08(47.34,66.44) | 49.83(41.99,59.13) | 49.61(41.68,59.03) | 47.60(40.03,56.58) | 41.17(34.53,49.07) | -30.93(-46.3,-15.55) | -11.93 (-15.87, -7.98) | <.0001 |
| **Lipid biomarkers** |  |  |  |  |  |  |  |  |  |
| High density lipoprotein (mg/dL) | ^b^MV model | 46.19(44.52,47.92) | 46.06(44.39,47.79) | 45.96(44.28,47.71) | 46.83(45.12,48.61) | 47.02(45.3,48.82) | 1.79(-1.5,5.08) | 0.83 (-0.01, 1.68) | 0.0533 |
| n= 4421 | ^c^MV+ BMI | 45.82(44.22,47.48) | 45.91(44.30,47.58) | 45.74(44.12,47.41) | 46.09(44.46,47.78) | 45.72(44.09,47.41) | -0.23(-3.42,2.97) | 0.09 (-0.73, 0.91) | 0.8309 |
|  |  |  |  |  |  |  |  |  |  |
| Low density lipoprotein (mg/dl) | ^b^MV model | 119.31(115.30,123.33) | 116.12(112.1,120.15) | 116.57(112.51,120.64) | 115.52(111.46,119.59) | 115.21(111.12,119.29) | -3.44(-6.68,-0.42) | -0.99(-1.80,-0.19) | 0.0159 |
| n = 4420 | ^c^MV+ BMI | 119.22(115.21,123.23) | 116.09(112.06,120.11) | 116.52(112.45,120.58) | 115.33(111.27,119.40) | 114.87(110.77,118.97) | -3.65(-6.91,-0.60) | -1.07(-1.88,-0.26) | 0.0096 |
|  |  |  |  |  |  |  |  |  |  |
| Triglycerides (mg/dl) | ^b^MV model | 135.53(126.62,145.06) | 130.91(122.28,140.15) | 133.29(124.44,142.78) | 127.09(118.65,136.14) | 126.7(118.24,135.77) | -6.73(-12.81,-0.65) | -2.50 (-4.06, -0.94) | 0.0017 |
| n = 4421 | ^c^MV+ BMI | 137.23(128.42,146.65) | 131.55(123.08,140.6) | 134.33(125.61,143.66) | 130.32(121.85,139.38) | 132.45(123.78,141.73) | -3.54(-9.51,2.42) | -1.33 (-2.86, 0.20) | 0.0879 |
|  |  |  |  |  |  |  |  |  |  |
| Total Cholesterol - calculated  (mg/dL) | ^b^MV model | 200.63(196.20,205.07) | 195.60(191.15,200.05) | 196.45(191.96,200.94) | 194.9(190.41,199.38) | 194.75(190.24,199.26) | -2.93(-5.02, -0.93) | -0.85(-1.38, -0.32) | 0.0016 |
| n = 4420 | ^c^MV+ BMI | 200.52(196.08,204.95) | 195.55(191.10,200.00) | 196.38(191.89,200.86) | 194.67(190.17,199.16) | 194.35(189.82,198.87) | -3.08(-5.18, -1.07) | -0.91(-1.44, -0.38) | 0.0008 |
|  |  |  |  |  |  |  |  |  |  |
| TG/HDL^d^ ratio | ^b^MV model | 2.93(2.67,3.22) | 2.84(2.59,3.12) | 2.9(2.64,3.19) | 2.71(2.47,2.98) | 2.69(2.45,2.96) | -8.52(-16.92, -0.13) | -3.33 (-5.49, -1.18) | 0.0024 |
| n= 4421 | ^c^MV+ BMI | 2.99(2.74,3.28) | 2.87(2.62,3.14) | 2.94(2.68,3.22) | 2.83(2.58,3.10) | 2.90(2.64,3.18) | -3.32(-11.46,4.83) | -1.42 (-3.51, 0.67) | 0.1826 |
|  |  |  |  |  |  |  |  |  |  |
| TC/HDL^d^ ratio | ^b^MV model | 4.27(4.11,4.43) | 4.17(4.01,4.34) | 4.20(4.04,4.36) | 4.09(3.93,4.25) | 4.07(3.91,4.23) | -4.84(-8.27, -1.42) | -1.68 (-2.56, -0.80) | 0.0002 |
| n = 4420 | ^c^MV+ BMI | 4.3(4.14,4.46) | 4.18(4.03,4.34) | 4.22(4.06,4.38) | 4.15(4.00,4.31) | 4.17(4.02,4.34) | -2.97(-6.31,0.38) | -0.99 (-1.85, -0.13) | 0.0239 |
| **Insulin response biomarkers** |  |  |  |  |  |  |  |  |  |
| Fasting glucose(mg/dL) | ^b^MV model | 99.24(97.83,100.67) | 98.76(97.35,100.18) | 98.45(97.05,99.88) | 97.92(96.53,99.34) | 97.44(96.03,98.88) | -1.83(-3.08, -0.57) | -0.60 (-0.92, -0.28) | 0.0002 |
| n = 3701 | ^c^MV+ BMI | 99.40(98.04,100.78) | 98.80(97.45,100.18) | 98.55(97.19,99.93) | 98.44(97.08,99.82) | 98.35(96.96,99.75) | -1.07(-2.28,0.15) | -0.32 (-0.64, -0.01) | 0.0415 |
|  |  |  |  |  |  |  |  |  |  |
| Fasting insulin (uIU/mL | ^b^MV model | 7.74(7.14,8.39) | 7.52(6.94,8.15) | 7.09(6.54,7.69) | 6.53(6.02,7.08) | 6.11(5.62,6.63) | -23.69(-30.75, -16.64) | -8.73 (-10.54, -6.92) | <.0001 |
| n = 3701) | ^c^MV+ BMI | 7.88(7.35,8.45) | 7.56(7.05,8.11) | 7.17(6.68,7.69) | 6.92(6.45,7.43) | 6.77(6.3,7.27) | -15.24(-21.38, -9.10) | -5.62 (-7.20, -4.05) | <.0001 |
|  |  |  |  |  |  |  |  |  |  |
| HOMA-IRd | ^b^MV model | 1.90(1.74,2.07) | 1.83(1.68,2.00) | 1.72(1.58,1.88) | 1.58(1.45,1.72) | 1.47(1.35,1.61) | -25.52(-33.09, -17.95) | -9.33 (-11.27, -7.40) | <.0001 |
| n = 3701 | ^c^MV+ BMI | 1.93(1.80,2.08) | 1.84(1.71,1.99) | 1.74(1.62,1.88) | 1.68(1.56,1.81) | 1.64(1.52,1.77) | -16.31(-22.86, -9.75) | -5.95 (-7.63, -4.27) | <.0001 |
| ^a^Values are absolute back-transformed biomarker concentrations (beta coefficients) since values were naturally log-transformed prior to analysis, and the p-value of < 0.05 represent statistically significant findings.  Beta coefficients were derived from  ^b^MV (multivariable) adjusted, and ^c^multivariable + BMI (body mass index) adjusted linear regression models.  Multivariable linear regression models were adjusted for total energy intake, race/ethnicity (White, African American, Asian, Hispanic, others), marital status (married, single, divorced/separated, widowed), education (less than high school, high school, college and higher), physical activity (continuous), smoking status (never, past, current), pack-years of smoking (continuous), number of nutritional supplements used (continuous), total alcohol intake (continuous, servings/day), aspirin/other NSAID use (yes vs no), baseline comorbidity score conditions (continuous).  ^d^TG, triglycerides; HDL, high-density lipoprotein; TC, total cholesterol; HOMA-IR, homeostatic model assessment of insulin resistance. | | | | | | | | | |

| **Supplementary Table 10. Longitudinal associations of dietary indices with circulating biomarkers of renal function, adipokines, insulin response, and lipids** | | | | | | | | |  |
| --- | --- | --- | --- | --- | --- | --- | --- | --- | --- |
|  | **Statistical Models** | **Quintile 1** | **Quintile 2** | **Quintile 3** | **Quintile 4** | **Quintile 5** | **Percentage difference: Q5-Q1** | **PD per SD** | **p-value** |
| **Reversed Empirical Dietary Index for Hyperinsulinemia (rEDIH)** | | | | | | | | | |
| **Renal functions biomarkers** |  |  |  |  |  |  |  |  |  |
| Serum creatinine (mg/dl) | ^b^MV model | 1.13(1.05,1.23) | 1.12(1.03,1.22) | 1.08(1,1.17) | 1.09(1,1.18) | 1.06(0.98,1.15) | -6.82(-13.06, -0.57) | -2.49 (-4.08, -0.91) | **0.0022** |
| n = 691 | ^c^MV+ BMI | 1.13(1.04,1.23) | 1.12(1.03,1.21) | 1.08(1,1.17) | 1.09(1,1.18) | 1.06(0.98,1.16) | -5.94(-12.3,0.42) | -2.23 (-3.84, -0.62) | **0.0067** |
| Serum cystatin C (mg/l) | ^b^MV model | 1.12(1.02,1.23) | 1.10(1.00,1.21) | 1.08(0.98,1.18) | 1.10(1.00,1.21) | 1.04(0.95,1.15) | -6.64(-13.86,0.57) | -1.89 (-3.73, -0.06) | **0.0435** |
| n = 691 | ^c^MV+ BMI | 1.1(1.01,1.21) | 1.10(1.00,1.21) | 1.08(0.98,1.18) | 1.10(1.01,1.21) | 1.06(0.96,1.16) | -4.16(-11.43,3.11) | -1.16 (-3.00, 0.69) | 0.2192 |
| **Adipokines** |  |  |  |  |  |  |  |  |  |
| Adiponectin (microg/mL) | ^b^MV model | 10.12(8.06,12.71) | 11.28(9.02,14.1) | 10.96(8.72,13.76) | 10.72(8.53,13.46) | 11.3(9.03,14.14) | 11.06(-5.87,28.00) | 4.42 (0.13, 8.72) | **0.0437** |
| n = 815 | ^c^MV+ BMI | 10.22(8.15,12.83) | 11.31(9.05,14.12) | 10.93(8.71,13.71) | 10.63(8.47,13.33) | 11.13(8.9,13.92) | 8.54(-8.48,25.56) | 3.64 (-0.68, 7.96) | 0.0990 |
| Leptin (ng/mL) | ^b^MV model | 11.39(8.91,14.56) | 10.22(8.04,12.99) | 10.70(8.36,13.67) | 10.10(7.9,12.91) | 8.48(6.66,10.8) | -29.52(-47.76, -11.27) | -8.92 (-13.56, -4.28) | **0.0002** |
| n = 815 | ^c^MV+ BMI | 10.69(8.71,13.11) | 10.05(8.23,12.27) | 10.89(8.87,13.36) | 10.66(8.7,13.08) | 9.34(7.64,11.42) | -13.53(-28.84,1.79) | -3.73 (-7.63, 0.17) | 0.0611 |
| **Insulin response biomarkers** |  |  |  |  |  |  |  |  |  |
| Fasting glucose(mg/dL) | ^b^MV model | 92.33(86.12,98.99) | 94.25(87.91,101.05) | 92.33(86.1,99) | 91.45(85.31,98.03) | 93.15(87.00,99.75) | 0.89(-2.53,4.31) | -0.02 (-0.88, 0.85) | 0.9715 |
| n = 689 | ^c^MV+ BMI | 92.30(86.09,98.97) | 94.25(87.9,101.05) | 92.33(86.1,99.01) | 91.45(85.31,98.03) | 93.18(87.01,99.78) | 0.94(-2.51,4.4) | -0.0037 (-0.88, 0.87) | 0.9934 |
| Fasting insulin(uIU/mL) | ^b^MV model | 15.24(11.34,20.48) | 17.36(12.92,23.34) | 16.84(12.52,22.64) | 16.19(12.05,21.75) | 15.97(11.95,21.36) | 4.72(-9.81,19.26) | -1.35 (-5.04, 2.34) | 0.4735 |
| n = 689 | ^c^MV+ BMI | 15.27(11.35,20.52) | 17.36(12.91,23.35) | 16.83(12.51,22.64) | 16.19(12.05,21.75) | 15.95(11.92,21.33) | 4.37(-10.3,19.04) | -1.51 (-5.24, 2.21) | 0.4257 |
| HOMA-IR^d^ | ^b^MV model | 3.47(2.53,4.77) | 4.04(2.94,5.54) | 3.84(2.8,5.27) | 3.66(2.67,5.01) | 3.67(2.69,5.01) | 5.62(-9.92,21.16) | -1.37 (-5.31, 2.58) | 0.4977 |
| n = 816 | ^c^MV+ BMI | 3.48(2.54,4.77) | 4.04(2.94,5.55) | 3.84(2.79,5.27) | 3.65(2.67,5.01) | 3.67(2.69,5.01) | 5.31(-10.37,20.99) | -1.52 (-5.50, 2.46) | 0.4554 |
| **Reversed Empirical Dietary Inflammatory Pattern (rEDIP)** | | | | | | | | | |
| **Renal functions biomarkers** | ^b^MV model |  |  |  |  |  |  |  |  |
| Serum creatinine (mg/dl) | ^c^MV+ BMI | 1.13(1.05,1.23) | 1.09(1.00,1.18) | 1.12(1.03,1.21) | 1.08(0.99,1.17) | 1.05(0.97,1.14) | -7.74(-14.24, -1.24) | -2.43 (-4.08, -0.79) | **0.0039** |
| n = 691 | ^b^MV model | 1.13(1.05,1.22) | 1.08(1.00,1.18) | 1.12(1.03,1.22) | 1.08(1.00,1.17) | 1.05(0.97,1.14) | -7.46(-13.95, -0.96) | -2.26 (-3.91, -0.60) | **0.0076** |
| Serum cystatin C (mg/l) | ^c^MV+ BMI | 1.12(1.02,1.23) | 1.10(1,1.21) | 1.09(0.99,1.20) | 1.08(0.98,1.19) | 1.04(0.95,1.14) | -7.27(-14.79,0.26) | -2.50 (-4.41, -0.60) | **0.0101** |
| n = 691 |  | 1.12(1.02,1.22) | 1.09(1,1.20) | 1.10(1.00,1.21) | 1.09(0.99,1.20) | 1.05(0.95,1.15) | -6.59(-14.02,0.84) | -2.06 (-3.94, -0.17) | **0.0331** |
| **Adipokines** |  |  |  |  |  |  |  |  |  |
| Adiponectin (microg/mL) | ^b^MV model | 10.70(8.56,13.37) | 10.48(8.36,13.13) | 11.34(9.05,14.21) | 11.14(8.86,14.01) | 11.53(9.16,14.53) | 7.51(-9.94,24.96) | 4.16 (-0.25, 8.57) | **0.0650** |
| n = 815 | ^c^MV+ BMI | 10.70(8.57,13.36) | 10.55(8.43,13.20) | 11.22(8.96,14.05) | 11.00(8.75,13.83) | 11.48(9.12,14.45) | 7.03(-10.35,24.42) | 3.90 (-0.50, 8.29) | 0.0829 |
| Leptin (ng/mL) | ^b^MV model | 10.23(8.05,13.01) | 11.60(9.1,14.79) | 9.24(7.24,11.78) | 8.94(6.98,11.44) | 9.31(7.26,11.94) | -9.43(-28.24,9.38) | -5.21 (-10.00, -0.42) | **0.0333** |
| n = 815 | ^c^MV+ BMI | 10.24(8.38,12.50) | 11.13(9.1,13.63) | 9.89(8.07,12.10) | 9.66(7.86,11.86) | 9.60(7.81,11.8) | -6.43(-22.07,9.22) | -3.56 (-7.53, 0.41) | 0.0793 |
| **Insulin response biomarkers** |  |  |  |  |  |  |  |  |  |
| Fasting glucose(mg/dL) | ^b^MV model | 93.04(86.79,99.75) | 94.17(87.84,100.95) | 92.10(86,98.63) | 91.23(85.1,97.81) | 93.63(87.35,100.36) | 0.63(-2.92,4.18) | -0.29 (-1.20, 0.63) | 0.5403 |
| n = 816 | ^c^MV+ BMI | 93.04(86.78,99.75) | 94.17(87.83,100.96) | 92.1(85.99,98.64) | 91.23(85.09,97.82) | 93.63(87.35,100.36) | 0.63(-2.92,4.18) | -0.28 (-1.20, 0.64) | 0.5466 |
| Fasting insulin(uIU/mL) | ^b^MV model | 16.44(12.23,22.09) | 17.87(13.30,24.01) | 15.77(11.79,21.09) | 16.60(12.35,22.31) | 15.30(11.39,20.54) | -7.19(-22.26,7.88) | -3.25 (-7.13, 0.64) | 0.1020 |
| n = 816 | ^c^MV+ BMI | 16.44(12.23,22.09) | 17.89(13.31,24.04) | 15.74(11.76,21.06) | 16.58(12.33,22.28) | 15.29(11.39,20.53) | -7.26(-22.34,7.82) | -3.30 (-7.20, 0.59) | 0.0966 |
| HOMA-IR^d^ | ^b^MV model | 3.78(2.75,5.18) | 4.15(3.03,5.7) | 3.59(2.63,4.89) | 3.74(2.73,5.13) | 3.54(2.58,4.85) | -6.56(-22.68,9.56) | -3.53 (-7.69, 0.62) | 0.0962 |
| n = 816 | ^c^MV+ BMI | 3.78(2.75,5.18) | 4.16(3.03,5.71) | 3.58(2.62,4.89) | 3.73(2.72,5.12) | 3.53(2.58,4.84) | -6.63(-22.76,9.50) | -3.59 (-7.75, 0.58) | 0.0917 |
| **Healthy Eating Index (HEI-2020)** | | | | | | | | | |
| **Renal functions biomarkers** |  |  |  |  |  |  |  |  |  |
| Serum creatinine (mg/dl) | ^b^MV model | 1.10(1.01,1.19) | 1.09(1.01,1.19) | 1.11(1.02,1.21) | 1.09(1.00,1.18) | 1.07(0.98,1.16) | -2.81(-9.24,3.62) | -1.05 (-2.68, 0.58) | 0.2078 |
| n = 691 | ^c^MV+ BMI | 1.10(1.01,1.19) | 1.09(1.00,1.19) | 1.11(1.02,1.21) | 1.09(1.01,1.18) | 1.07(0.99,1.16) | -2.36(-8.80,4.07) | -0.86 (-2.50, 0.77) | 0.3016 |
| Serum cystatin C (mg/l) | ^b^MV model | 1.09(0.99,1.19) | 1.09(0.99,1.20) | 1.13(1.03,1.24) | 1.07(0.98,1.18) | 1.05(0.95,1.15) | -4.05(-11.44,3.34) | -1.35 (-3.23, 0.53) | 0.1593 |
| n = 691 | ^c^MV+ BMI | 1.09(1.00,1.19) | 1.09(0.99,1.20) | 1.13(1.03,1.23) | 1.08(0.98,1.18) | 1.06(0.96,1.16) | -2.99(-10.31,4.33) | -0.90 (-2.77, 0.96) | 0.3419 |
| **Adipokines** |  |  |  |  |  |  |  |  |  |
| Adiponectin (microg/mL) | ^b^MV model | 10.75(8.58,13.46) | 10.64(8.50,13.33) | 11.06(8.82,13.87) | 11.03(8.77,13.89) | 11.36(9.06,14.26) | 5.59(-11.84,23.02) | 2.86 (-1.59, 7.32) | 0.2083 |
| n = 815 | ^c^MV+ BMI | 10.75(8.59,13.45) | 10.64(8.50,13.31) | 11.13(8.88,13.95) | 11.00(8.74,13.83) | 11.15(8.89,13.98) | 3.67(-13.78,21.11) | 2.23 (-2.23, 6.70) | 0.3276 |
| Leptin (ng/mL) | ^b^MV model | 10.41(8.17,13.28) | 10.69(8.38,13.63) | 10.36(8.11,13.23) | 9.83(7.67,12.60) | 8.59(6.72,10.98) | -19.26(-38.09, -0.42) | -6.67 (-11.50, -1.84) | **0.0069** |
| n = 815 | ^c^MV+ BMI | 10.40(8.50,12.72) | 10.74(8.78,13.15) | 9.96(8.13,12.21) | 10.04(8.17,12.35) | 9.66(7.88,11.85) | -7.34(-23.06,8.39) | -2.66 (-6.69, 1.37) | 0.1963 |
| **Insulin response biomarkers** |  |  |  |  |  |  |  |  |  |
| Fasting glucose(mg/dL) | ^b^MV model | 93.43(87.16,100.15) | 92.41(86.21,99.04) | 92.24(86.05,98.89) | 92.93(86.7,99.61) | 92.69(86.42,99.41) | -0.80(-4.31,2.71) | -0.19 (-1.09, 0.71) | 0.6749 |
| n = 816 | ^c^MV+ BMI | 93.43(87.16,100.16) | 92.41(86.22,99.06) | 92.23(86.03,98.88) | 92.95(86.71,99.63) | 92.7(86.43,99.43) | -0.78(-4.30,2.73) | -0.19 (-1.09, 0.71) | 0.6835 |
| Fasting insulin(uIU/mL) | ^b^MV model | 17.37(12.95,23.30) | 15.44(11.51,20.70) | 16.08(11.98,21.57) | 16.59(12.37,22.25) | 15.08(11.21,20.27) | -14.14(-28.98,0.7) | -2.26 (-6.08, 1.56) | 0.2467 |
| n = 816 | ^c^MV+ BMI | 17.36(12.94,23.30) | 15.42(11.50,20.68) | 16.10(12.00,21.61) | 16.56(12.35,22.21) | 15.05(11.19,20.24) | -14.31(-29.18,0.55) | -2.33 (-6.15, 1.50) | 0.2332 |
| HOMA-IR^d^ | ^b^MV model | 4.01(2.93,5.49) | 3.52(2.57,4.82) | 3.66(2.67,5.01) | 3.81(2.78,5.21) | 3.45(2.51,4.74) | -14.94(-30.81,0.94) | -2.45 (-6.53, 1.63) | 0.2399 |
| n = 816 | ^c^MV+ BMI | 4.01(2.92,5.49) | 3.52(2.57,4.82) | 3.67(2.68,5.02) | 3.80(2.78,5.20) | 3.44(2.51,4.73) | -15.09(-30.99,0.81) | -2.52 (-6.61, 1.58) | 0.2285 |

| ^a^Values are absolute back-transformed biomarker concentrations (beta coefficients) since values were naturally log-transformed prior to analysis, and the bolded numbers represent statistically significant findings (i.e., p < 0.05).  Beta coefficients were derived from ^b^MV (multivariable) adjusted, and ^c^multivariable + BMI (body mass index) adjusted linear regression models.  Multivariable linear regression models were adjusted for total energy intake, race/ethnicity (White, African American, Asian, Hispanic, others), marital status (married, single, divorced/separated, widowed), education (less than high school, high school, college and higher), physical activity (continuous), smoking status (never, past, current), pack-years of smoking (continuous), number of nutritional supplements used (continuous), total alcohol intake (continuous, servings/day), aspirin/other NSAID use (yes vs no), baseline comorbidity score conditions (continuous).  ^d^HOMA-IR, homeostatic model assessment of insulin resistance. |
| --- |

| **Supplementary Table 11. Multivariable-adjusted associations of dietary patterns with biomarkers in body mass index categories: A subgroup analysis** | | | | | | |
| --- | --- | --- | --- | --- | --- | --- |
|  |  | **Normal weight (18.5 to <25 kg/m^2^)** | |  | **Overweight/obese (25 to 50 kg/m^2^)** | |
|  | Sample size | Percent difference per 1 SD increment in dietary score | p-value | Sample size | Percent difference per 1 SD increment in dietary score | p-value |
| **rEDIH** |  |  |  |  |  |  |
| **Renal Functions biomarkers** |  |  |  |  |  |  |
| Serum creatinine (mg/dl) | 1210 | -2.49 (-3.86, -1.13) | 0.0004 | 3207 | -0.70 (-1.40, 0.00) | 0.0505 |
| Urine Creatinine (mg/dl) | 318 | -7.76 (-15.56, 0.04) | 0.0521 | 882 | -3.90 (-7.92, 0.13) | 0.0582 |
| Serum cystatin C (mg/l) | 319 | -0.96 (-3.75, 1.84) | 0.5028 | 896 | 1.12 (-0.05, 2.29) | 0.0616 |
| Serum sodium (mmol/L) | 1209 | -0.03 (-0.15, 0.10) | 0.6789 | 3204 | 0.05 (-0.01, 0.11) | 0.1217 |
| **Urine sodium (mmol/L)** | 338 | -2.63(-7.88,2.62) | 0.3258 | 933 | -5.78(-8.75, -2.8) | 0.0001 |
| Serum potassium (mmol/L) | 1209 | -0.64 (-1.17, -0.12) | 0.0164 | 3204 | -0.11 (-0.38, 0.17) | 0.4420 |
| Urine potassium (mmol/L) | 289 | -2.08 (-8.86, 4.69) | 0.5473 | 800 | -1.05 (-4.59, 2.50) | 0.5634 |
| Serum phosphate (mg/dL) | 1210 | 0.02 (-0.83, 0.88) | 0.9550 | 3207 | -0.60 (-1.07, -0.12) | 0.0134 |
| Urine phosphate (mg/dL) | 314 | -7.37 (-16.67, 1.94) | 0.1221 | 879 | -5.54 (-10.05, -1.03) | 0.0163 |
| **Lipid biomarkers** |  |  |  |  |  |  |
| High density lipoprotein (mg/dl) | 1210 | 0.42 (-1.30, 2.14) | 0.6324 | 3207 | 0.23 (-0.68, 1.14) | 0.6232 |
| Low density lipoprotein (mg/dl) | 1210 | -2.69(-4.54, -0.84) | 0.0044 | 3206 | 0.47(-0.55,1.5) | 0.3626 |
| Triglycerides (mg/dl | 1210 | -2.77 (-5.68, 0.14) | 0.0625 | 3207 | -1.00 (-2.76, 0.75) | 0.2613 |
| Total Cholesterol - calculated  (mg/dl) | 1210 | -3.19(-5.30, -1.07) | 0.0032 | 3206 | 8.45(-102.87,119.77) | 0.8817 |
| TG/HDL ratio | 1210 | -2.97(-7.06,1.12) | 0.1542 | 3206 | -1.39(-3.74,0.97) | 0.2480 |
| TC/HDL ratio | 1210 | -1.85(-3.55, -0.15) | 0.0328 | 3206 | -0.18(-1.13,0.78) | 0.7187 |
| **Insulin-response biomarkers** |  |  |  |  |  |  |
| Fasting glucose(mg/dL) | 1081 | -0.63 (-1.20, -0.06) | 0.0308 | 2591 | -0.29 (-0.66, 0.09) | 0.1326 |
| Fasting insulin(uIU/mL) | 1081 | -6.15 (-9.13, -3.18) | 0.0001 | 2591 | -3.51 (-5.54, -1.49) | 0.0007 |
| HOMA-IR | 1081 | -7.51(-10.68, -4.34) | <.0001 | 3132 | -4.79(-6.91, -2.66) | <.0001 |
| **rEDIP** |  |  |  |  |  |  |
| **Renal Functions biomarkers** |  |  |  |  |  |  |
| Serum creatinine (mg/dl) | 1210 | -1.66 (-2.97, -0.36) | 0.0127 | 3207 | -0.82 (-1.56, -0.08) | 0.0309 |
| Urine Creatinine (mg/dl) | 318 | -8.99 (-16.73, -1.25) | 0.0235 | 882 | -6.87 (-11.26, -2.47) | 0.0023 |
| Serum cystatin C (mg/l) | 319 | -0.15 (-2.93, 2.62) | 0.9142 | 896 | -0.43 (-1.71, 0.85) | 0.5074 |
| Serum sodium (mmol/L) | 1209 | -0.02 (-0.14, 0.10) | 0.7409 | 3204 | -0.02 (-0.09, 0.04) | 0.4885 |
| Urine sodium (mmol/L) | 338 | -7.03(-12.32, -1.74) | 0.0093 | 933 | -6.45(-9.67, -3.23) | <.0001 |
| Serum potassium (mmol/L) | 1209 | -0.17 (-0.67, 0.33) | 0.5131 | 3204 | -0.21 (-0.50, 0.09) | 0.1659 |
| Urine potassium (mmol/L) | 289 | -1.25 (-7.97, 5.47) | 0.7163 | 800 | -2.27 (-6.15, 1.60) | 0.2501 |
| Serum phosphate (mg/dL) | 1210 | 0.15 (-0.66, 0.97) | 0.7108 | 3207 | -0.08 (-0.59, 0.42) | 0.7453 |
| Urine phosphate (mg/dL) | 314 | -6.28 (-15.65, 3.10) | 0.1905 | 879 | -7.36 (-12.29, -2.43) | 0.0035 |
| **Lipid biomarkers** |  |  |  |  |  |  |
| High density lipoprotein (mg/dl) | 1210 | 1.21 (-0.43, 2.84) | 0.1481 | 3207 | 2.17 (1.21, 3.13) | <.0001 |
| Low density lipoprotein (mg/dl) | 1210 | -1.65(-3.42,0.11) | 0.0666 | 3206 | 0.36(-0.74,1.46) | 0.5217 |
| Triglycerides (mg/dl) | 1210 | -3.73 (-6.49, -0.96) | 0.0084 | 3207 | -4.04 (-5.90, -2.18) | <.0001 |
| Total Cholesterol - calculated  (mg/dl) | 1210 | -1.97(-3.99,0.05) | 0.0556 | 3206 | -0.16(-1.35,1.04) | 0.7993 |
| TG/HDL ratio | 1210 | -4.96(-8.84, -1.08) | 0.0124 | 3206 | -6.24(-8.76, -3.71) | <.0001 |
| TC/HDL ratio | 1210 | -2.14(-3.76, -0.53) | 0.0094 | 3206 | -2.22(-3.24, -1.19) | <.0001 |
| **Insulin-response biomarkers** |  |  |  |  |  |  |
| Fasting glucose(mg/dL) | 1081 | -0.37 (-0.91, 0.18) | 0.1902 | 2591 | 0.03 (-0.37, 0.43) | 0.8859 |
| Fasting insulin(uIU/mL) | 1081 | -6.07 (-8.92, -3.23) | <.0001 | 2591 | -4.09 (-6.24, -1.94) | 0.0002 |
| HOMA-IR | 1081 | -6.96(-9.98, -3.94) | <.0001 | 3132 | -5.32(-7.61, -3.04) | <.0001 |
| **HEI-2020** |  |  |  |  |  |  |
| **Renal Functions biomarkers** |  |  |  |  |  |  |
| Serum creatinine (mg/dl) | 1210 | -1.77 (-3.04, -0.51) | 0.0061 | 3207 | -0.35 (-1.09, 0.40) | 0.3620 |
| Urine Creatinine (mg/dl) | 318 | -10.42 (-16.89, -3.94) | 0.0018 | 882 | -9.54 (-13.72, -5.36) | 0.0000 |
| Serum cystatin C (mg/l) | 319 | -1.44 (-3.75, 0.88) | 0.2241 | 896 | -0.05 (-1.28, 1.17) | 0.9332 |
| Serum sodium (mmol/L) | 1209 | -0.09 (-0.20, 0.03) | 0.1332 | 3204 | -0.08 (-0.14, -0.01) | 0.0226 |
| Urine sodium (mmol/L) | 338 | -9.17(-13.53, -4.82) | <.0001 | 933 | -7.1(-10.27, -3.92) | <.0001 |
| Serum potassium (mmol/L) | 1209 | -0.16 (-0.65, 0.33) | 0.5155 | 3204 | 0.16 (-0.13, 0.46) | 0.2702 |
| Urine potassium (mmol/L) | 289 | 0.17 (-5.49, 5.83) | 0.9528 | 800 | -0.69 (-4.32, 2.94) | 0.7102 |
| Serum phosphate (mg/dL) | 1210 | -0.27 (-1.06, 0.52) | 0.5039 | 3207 | 0.13 (-0.38, 0.63) | 0.6224 |
| Urine phosphate (mg/dL) | 314 | -11.76 (-19.30, -4.23) | 0.0024 | 879 | -10.95 (-15.64, -6.26) | <.0001 |
| **Lipid biomarkers** |  |  |  |  |  |  |
| High density lipoprotein (mg/dl) | 1210 | -0.36 (-1.95, 1.23) | 0.6611 | 3207 | 0.59 (-0.38, 1.56) | 0.2337 |
| Low density lipoprotein (mg/dl) | 1210 | -2.88(-4.62, -1.13) | 0.0013 | 3206 | -0.52(-1.62,0.58) | 0.3573 |
| Triglycerides (mg/dl) | 1210 | -1.54 (-4.23, 1.16) | 0.2645 | 3207 | -1.57 (-3.45, 0.30) | 0.0994 |
| Total Cholesterol - calculated  (mg/dl) | 1210 | -3.42(-5.42, -1.43) | 0.0008 | 3206 | -0.96(-2.16,0.24) | 0.1177 |
| TG/HDL ratio | 1210 | -0.39(-4.25,3.47) | 0.8426 | 3206 | -2.68(-5.21, -0.14) | 0.0387 |
| TC/HDL ratio | 1210 | -1.22(-2.83,0.39) | 0.1362 | 3206 | -1.30(-2.33, -0.27) | 0.0135 |
| **Insulin-response biomarkers** |  |  |  |  |  |  |
| Fasting glucose(mg/dL) | 1081 | -0.30 (-0.83, 0.23) | 0.2724 | 2591 | -0.49 (-0.87, -0.10) | 0.0144 |
| Fasting insulin(uIU/mL) | 1081 | -5.89 (-8.66, -3.11) | <.0001 | 2591 | -7.11 (-9.20, -5.02) | <.0001 |
| HOMA-IR | 1081 | -6.16(-9.21, -3.11) | <.0001 | 3132 | -8.64(-10.92, -6.35) | <.0001 |
| Values are percentage difference (PD) in biomarker concentrations for each 1 standard deviation increment in the dietary index score in subgroups defined by (A) Normal weight (18.5 to <25 kg/m^2^) and (B) overweight/obese (25 to 50 kg/m^2^). Biomarker concentrations were log transformed using natural logs. Values (beta coefficients) presented were obtained via multivariable-adjusted linear regression models adjusted for the following variables: total energy intake, race/ethnicity (White, African American, Asian, Hispanic, others), marital status (married, single, divorced/separated, widowed), education (less than high school, high school, college and higher), physical activity (continuous), smoking status (never, past, current), pack-years of smoking (continuous), number of nutritional supplements used (continuous), total alcohol intake (continuous, servings/day), aspirin/other NSAID use (yes vs no), baseline comorbidity score conditions (continuous). p-value of <0.05 is considered statistically significant. | | | | | | |
